# Effect of void-carbon on blue-shifted luminescence in TADF molecules by theoretical simulations

**DOI:** 10.3389/fchem.2023.1094574

**Published:** 2023-01-26

**Authors:** Boyuan Zhang, Haoyang Xu, Yumin Xia, Jin Wen, Meifang Zhu

**Affiliations:** State Key Laboratory for Modification of Chemical Fibers and Polymer Materials, College of Materials Science and Engineering, Donghua University, Shanghai, China

**Keywords:** TADF, donor-void-acceptor, void-carbon, simulations, antiaromatic, blue-emission

## Abstract

Thermally activated delayed fluorescence (TADF) molecules have a theoretical 100% photoluminescence quantum yield in comparison with traditional fluorescent materials, leading to broad application in organic light-emitting diode (OLED). However, the application of TADF molecules with conjugated donor-acceptor structures in blue OLED remains a challenge due to their generally narrow energy gap between frontier molecular orbitals. Recently, a strategy has been approved in the improvement of the performance in TADF, in which void-carbon atoms between donor and acceptor fragments (donor-void-acceptor (D-v-A)) could regulate blue light emission. In this study, we first select three reported isomers followed by two proposed D-v-A TADF isomers to verify the feasibility of the void-carbon strategy through evaluation of the electronic structures in the excited state and photophysical properties. We further proposed a series of TADF molecules by replacing different donor and acceptor fragments to assess the applicability of the void-carbon strategy from the aspect of simulations in electronic structures, different properties of donor and acceptor fragments, photophysical properties, and analysis in the molecular conjugation. The results indicate that void-carbon strategy has conditional feasibility and applicability. Donor-acceptor molecular properties could be tuned through void-carbon strategy on aromatic acceptor fragments during the selection of promising candidates of TADF molecules. However, the void-carbon strategy does not work for the molecules with antiaromatic acceptor fragments, where the steric hindrance of the molecules plays a dominant role. Our work provides insightful guidance for the design of the blue-emission TADF molecules.

## 1 Introduction

Since Adachi and his coworker discovered a green light-emitting thermally activated delayed fluorescence (TADF) molecule with a high external quantum efficiency of 19.3% (Uoyama et al., 2012), TADF emitter has attracted intensive attention in organic light-emitting diode (OLED) field (Chen and Song, 2019; Xue et al., 2020). The reduction in the singlet-triplet gap (Δ*E*_ST_) in TADF molecules facilities the reverse intersystem crossing (RISC), in which an efficient up-conversion of triplet into singlet exciton can reach photoluminescence quantum yield of 100% theoretically (Fan et al., 2018; Cui et al., 2020). Taking this advantage, TADF molecules could have a high external quantum efficiency compared with traditional fluorescent materials, showing promising applications in OLED (Zhang et al., 2014; Hu et al., 2022). However, it is still a challenge to prepare high-efficient TADF emitters with a pure blue emission, prohibiting industrial production in blue OLED (Yook and Lee, 2012; Zhang et al., 2020). Bottlenecks in blue TADF are low external quantum efficiency (Geng et al., 2017; Shi et al., 2018), roll-off (Masui et al., 2013; Lee et al., 2021), broad luminescence spectrum (Li et al., 2020; Lee et al., 2021), difficulty in achieving deep-blue emission (Yang et al., 2015; Tagare and Vaidyanathan, 2018), and short lifetime (Monkman, 2022). To conquer the difficulty in TADF, practical principle in molecular design is required to discover more TADF materials with higher stability and efficient blue emission.

In recent years, great progress has been made in the study of blue-emission TADF, especially in tuning the emission wavelength of TADF by adjusting numerous donor and acceptor fragments (Fan et al., 2016; Huang et al., 2017). Additionally, the steric hindrance effect on the emission can be achieved by adjusting the position of methyl groups, resulting in the prolongation of a lifetime in the delayed fluorescence by an order of magnitude as well as an increase in the external quantum efficiency (Stachelek et al., 2019; Shi et al., 2022). Different push-pull groups connected by covalent bonds in donor-acceptor (D-A) type TADF can be used to control molecular conjugation, enhancing intramolecular charge transfer. Executing this design strategy, deep-blue emission with high purity has been achieved in TADF, such as DMACN-B, and PXZN-B (Khan et al., 2021). Recently, Zhang et al. introduced void-carbon atoms in D-A TADF, which was denoted as donor-void-acceptor (D-v-A) for improving the photophysical properties in TADF (Zhang et al., 2022). The energy difference between the highest occupied molecular orbital (HOMO) and lowest unoccupied molecular orbital (LUMO) could be adjusted by the position of void-carbon, leading to blue-shifted emission in D-v-A TADF. Even the emission performance in D-v-A TADF has not reached industry requirements, understanding the mechanism of the blue-shifted emission could provide constructive guidelines for improving the fluorescence in TADF.

The properties of excited states in TADF can be obtained by calculations in the electronic structure, which could be used to reveal mechanism of light-emitting materials (Yook and Lee, 2012; Masui et al., 2013; Geng et al., 2017; Zhang et al., 2020). Energy difference between S_1_ and T_1_ states can be evaluated by Eq. (1)

$$\Delta E_{\text{ST}}=2\iint \Phi_{L}\left(1\right)\Phi_{H}\left(2\right)\frac{e^{2}}{r_{1}-r_{2}}\Phi_{L}\left(2\right)\Phi_{H}\left(1\right)dr_{1}\;dr_{2},$$ (1)

where *Φ_L_* (1) and *Φ_H_* (2) stand for the wave function of LUMO and HOMO for first and second electrons respectively, and the electronic coordinates are represented by vector *r*. Since the overlap of molecular orbitals can affect Δ*E*_ST_, which could be used to adjust RISC rate (Tao et al., 2014; Fan et al., 2018). In addition, the process in internal conversion and intersystem crossing could be evaluated using the thermal vibrational correlation function (TVCF) (Shuai, 2020), and structure-property relationships can be explored by analysis in vibrational modes and coupled oscillators. Shuai’s group has developed a method to evaluate photophysical properties based on the recombination energy and configurational change by using TVCF, which implies that huge steric effects would reduce the non-radiative transition (Peng et al., 2017). However, the gap between the theoretical model and experimental phenomena remains still, which makes understanding the mechanism of photoluminescence challenging (Sun et al., 2019; Khan et al., 2021; Zhang et al., 2022).

**FIGURE 1:**
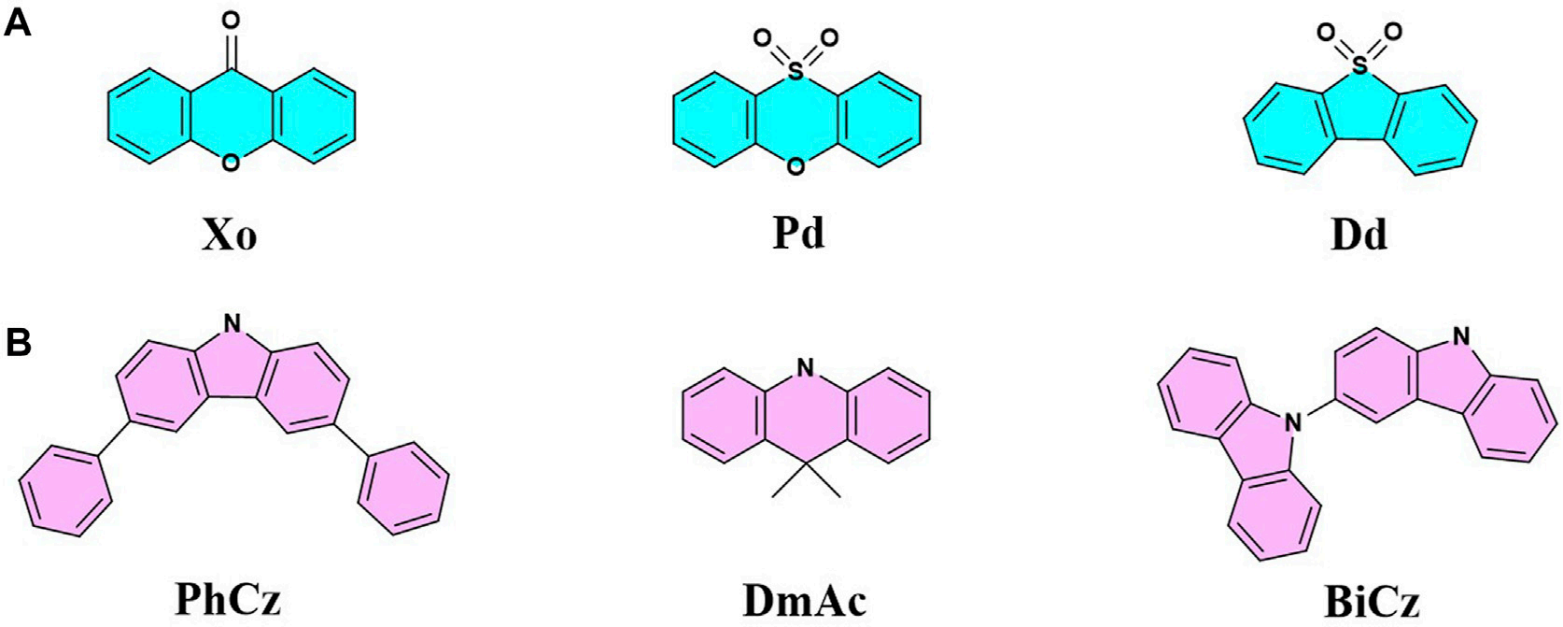
Structures of **(A)** acceptor and **(B)** donor fragments.

The role of void-carbon in the emission mechanism of D-v-A TADF is still unrevealed, which could be explored using computational simulations in the photophysical properties. The absorption and emission spectra, frontier molecular orbitals, energy difference in the excited states, and spin-orbit coupling (SOC) in different molecular configurations can be simulated by density functional theory (DFT) and time-dependent DFT (TDDFT) (Peng et al., 2017; Fan et al., 2018). In addition, the radiative decay rate (*k*_r_), non-radiative decay rate (*k*_nr_), decay rates in intersystem crossing and its reverse process (*k*_ISC_ and *k*_RISC_) could be evaluated by TVCF theory (Shuai, 2020). We first apply this method to study three isomers, since we could compare the excited-state structures and photophysical properties predicted from our simulations with experimental measurements to verify computational methods. Then the feasibility and applicability of the void-carbon strategy will be further investigated on the other D-v-A structures by alternating donor and acceptor fragments, as shown in Figure 1. Our simulations will verify the conditions for the application of void-carbon strategy and provide designs guideline for improving blue-emission in TADF materials.

## 2 Computational details

In the study of the fluorescence mechanism of the D-v-A TADF, firstly, geometries of 36PCX in the ground (S_0_) and first singlet excited (S_1_) states were obtained by using DFT and TDDFT with B3LYP functional respectively (Becke, 1988). Then, based on the B3LYP optimized geometries, the absorption and emission spectra were simulated by calculating the vertical excitation energies with different functionals (B3LYP, MN15, PBE0, M06-2X, WB97XD, and CAM-B3LYP) (Becke, 1988; Adamo and Barone, 1999; Yanai et al., 2004; Chai and Head-Gordon, 2008; Zhao and Truhlar, 2008; Yu et al., 2016). We compared theoretical simulations with experimental spectra during the selection of DFT functionals. As it showed in Supplementary Table S1, the absorption and emission spectra obtained by B3LYP functional were 440 and 560 nm, which underestimated the excitation energy compared with the experiment data, while those calculated by M06-2X, WB97XD, and CAM-B3LYP overestimated the excitation energy. The absorption and emission spectra of 36PCX calculated by MN15 and PBE0 functionals were relatively close to the experimental spectra, thus we selected MN15 and PBE0 functionals for further geometry optimization in the ground and excited states. As shown in Supplementary Table S2, compared with the experimental data, the PBE0 functional underestimated the emission energy, but the MN15 functional could reproduce the experimental absorption and emission spectra well. Therefore, we selected MN15 functional in the following calculations with the cc-pVDZ basis set for the other systems (Dunning, 1989). All DFT and TDDFT calculations were carried out using the Gaussian 16, Revision C.02 package (Frisch et al., 2016).

Besides the energy gap between S_1_ and the first triplet excited state (T_1_), which were obtained from TDDFT calculations at the MN15/cc-pVDZ level.

As SOC would affect the transition between singlet and triplet excited states as well, which was calculated at the TD-MN15/cc-pVDZ level using ORCA 5.0.1 program package (Neese, 2005; 2022). *k*_r_, *k*_nr_, *k*_ISC_, and *k*_RISC_ were evaluated by MOMAP (Molecular Materials Property Prediction Package) (Shuai and Peng, 2014; 2017; Shuai, 2020). The radiative rate constant *k*_r_ was computed as follow

$$k_{r}\left(T\right)=\int \sigma_{\text{em}}\left(\omega ,T\right)d\omega ,$$ (2)

where $\sigma_{em}\left(\omega ,T\right)=\frac{4\omega^{3}}{3\hbar c^{3}}\sum_{u,v}P_{iv}{\left|\left\langle \Theta_{fu}\left|u_{fi}\right|\Theta_{iv}\right\rangle \right|}^{2}\delta \left(\omega_{iv,fu}-\omega \right)$ . Among them, *P*_*iv*_ was the initial-state Boltzmann distribution function. The nuclear vibrational wave functions were represented by Θ, and *u*_*fi*_ was denoted as the electronic transition dipole moment (Peng et al., 2013; Shuai et al., 2014; Shuai and Peng, 2014). According to the Franck-Condon principle and the delta function Fourier transform, *k*_nr_ can be evaluated as

$$k_{\text{nr}}=\underset{kl}{\sum }\frac{1}{\hbar^{2}}R_{\text{kl}}\int_{-\infty }^{\infty }dt\left[e^{i\omega_{if}t}Z_{i}^{-1}\rho_{ic,kl}\left(t,T\right)\right].$$ (3)

Here *ℏ* represents the reduced Planck constant. The non-adiabatic electronic coupling is $R_{kl}=\left\langle \Phi_{f}\left|{\hat{P}}_{fk}\right|\Phi_{i}\right\rangle \left\langle \Phi_{i}\left|{\hat{P}}_{fl}\right|\Phi_{f}\right\rangle$ . *Z*_*i*_ and *ρ*_*ic*,*kl*_(*t*, *T*) are the partition function and TVCF, respectively (Shuai and Peng, 2014; 2017). Analogously, the *k*_ISC_ between two electronic states in different spin states can be evaluated as

$$k_{\text{ISC}}=\frac{1}{\hbar^{2}}\left\langle \Phi_{f}\left|{\hat{H}}^{\text{SO}}\right|\Phi_{i}\right\rangle \int_{-\infty }^{\infty }dt\left[e^{i\omega_{if}t}Z_{i}^{-1}\rho_{\text{ISC}}\left(t,T\right)\right].$$ (4)

The derivation of all the formulas can be found in Shuai and his collaborators’ work (Peng et al., 2013; Shuai et al., 2014; Shuai and Peng, 2014). The molecular orbital delocalization index (ODI) is written as (Lu, 2020)

$${ODI}_{i}=0.01\times \underset{A}{\sum }{\left(\Theta_{\text{A, i}}\right)}^{2},$$ (5)

where Θ_A,i_ is the composition of atom A in the molecular orbital. The ODI is ranged from 0 to 100%. Nucleus-independent chemical shift (NICS(1)) was calculated at 1 Å above the ring plane by the gauge independent atomic orbital (GIAO) method to evaluate the aromaticity (Schleyer et al., 2001).

## 3 Results

**FIGURE 2:**
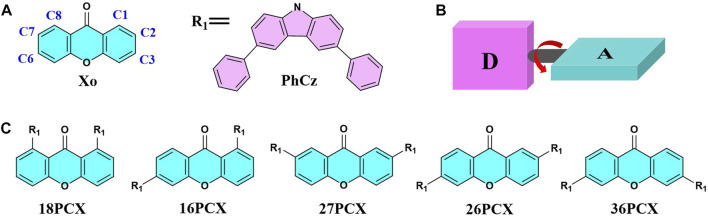
Structure diagram of Xo, PhCz **(A)**, and PCX series **(C)** investigated; Definition of the dihedral angle between different fragments in D-A type molecules **(B)**.

The energy gap between frontier orbitals will be increased by introducing the void-carbon in D-A TADF, leading to the change in the emission spectrum (Zhang et al., 2022). In this work, the feasibility and applicability of void-carbon are verified by studying the excited states and photophysical properties of D-A type molecules with different donors and acceptors. Firstly, we investigate the effect of the position of void-carbon on the electronic structures in D-A TADF, in which two moieties Xo (9H-xanthen-9-one) and PhCz (3,6-diphenyl-9H-carbazole) with different electron withdrawing and donating groups are used as acceptor and donor fragments respectively. We denote different isomers by the position of the void-carbon, illustrated in Figure 2.

### 3.1 Void-carbon effect on D-v-A TADF

Computational spectra are compared with experimental measurement in three isomers, 16PCX, 26PCX, and 36PCX with distances between void-carbon in descending order. Two more isomers, 18PCX and 27PCX are further proposed in this study in comparison with the experimentally reported isomers to verify the position effect of void-carbon on properties of PCX molecules (Figure 2). We obtain the optimized geometries in S_0_, S_1_, and T_1_ states using DFT and TDDFT calculations in the gas phase, followed by simulations in the photophysical properties.

#### 3.1.1 Excitation energies

**FIGURE 3:**
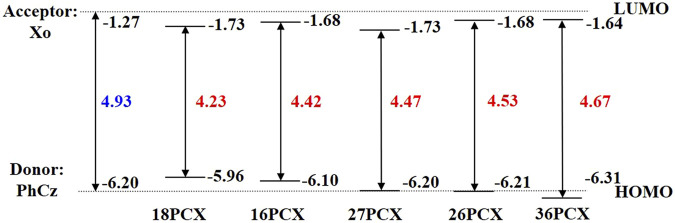
Comparison of HOMO-LUMO energy gaps of PCX series. The dotted lines represent energy levels of HOMO for PhCz and LUMO for Xo respectively.

Frontier molecular orbitals in acceptor molecules and these isomers are presented in Supplementary Figure S1 and Supplementary Figure S2 in the supplementary material, in which the orbital occupation on the C3 atom of the acceptor fragment Xo is negligible. We notice the separated HOMO and LUMO are mainly located in donor PhCz and acceptor Xo respectively, implying strong charge transfer (CT) character in D-A TADF. We compare the energy levels in D-A TADF with the isolated donor and acceptor moieties in Figure 3. The eigenvalues are -6.20 and -1.27 eV in HOMO and LUMO in PhCz and Xo respectively. In the experimentally measured isomers 16PCX, 26PCX, and 36PCX, energy levels in HOMO vary from -6.10, -6.20, to -6.31 eV respectively. In contrast, LUMOs locate closely in these isomers. In our proposed isomers 18PCX and 27PCX, the eigenvalues of LUMOs are both -1.73 eV, whereas HOMOs differ by 0.24 eV from each other. Based on the energy levels in these isomers, we find that the distance between occupied donor groups on Xo affects the location of HOMO in D-A TADF and it has limited influence on the energy level of LUMO. The DFT calculations show HOMO-LUMO gap increases from 4.23 to 4.67 eV in 18PCX and 36PCX, resulting in a blue-shifted emission in 36PCX.

Besides the position of void-carbon, the steric hindrance effect is another factor determining conjugation, electronic configuration, and photophysical properties of TADF molecules (Woo et al., 2020). When the donor fragments are changed from C1 to C3 and from C8 to C6, the dihedral angle between donor and acceptor fragments decreases, resulting in an increase of the conjugation and the oscillator strength in the excitation. Taking the experimental reported system for example firstly, when we only change one connection point, i.e. 16PCX, 26PCX, and 36PCX, changes in dihedral angles come from the steric hindrance effect. Due to the presence of void-carbon C3 only in 36PCX, the HOMO-LUMO gap is increased from 16PCX to 36PCX. On the other hand, when we change connecting positions of two donor fragments, namely 36PCX, 27PCX, and 18PCX, dihedral angles are increased with the reduction in the molecular conjugation. As it shows in Table 1, the dihedral angle reduces from 77.8° to 49.5° in 18PCX and 36PCX. Overlap between frontier molecular orbitals changes with the dihedral angles, leading to the change in the adiabatic energy gap Δ*E*_ST_ accordingly. Therefore, the largest conjugated molecule 36PCX has the largest HOMO-LUMO gap, resulting in a blue shift of emission wavelength to 410 nm among our studied systems. Moreover, we expect these PCX series should all present TADF properties since Δ*E*_ST_ in all molecules is less than 0.50 eV, which fulfills the energetic requirement in TADF (Uoyama et al., 2012).

**TABLE 1:** Excited state properties, dihedral angles, and photophysical properties of PCX series.

Emitters	Abs (nm)	Emi (nm)	Dihedral (°)	Δ*E*_ST_ (eV)	*k*_r_ (s^−1^)	*k*_nr_ (s^−1^)	*k*_ISC_ (s^−1^)	*k*_RISC_ (s^−1^)
18PCX	443	559	77.8/-50.7	0.12	3.92 × 10^2^	2.25 × 10^10^	1.68 × 10^7^	9.34 × 10^4^
16PCX	410	520	49.4/-56.7	0.08	2.02 × 10^2^	6.83 × 10^10^	9.73 × 10^4^	4.46 × 10^3^
27PCX	372	420	51.4/-51.4	0.39	1.80 × 10^6^	1.70 × 10^10^	2.82 × 10^7^	1.24 × 10^1^
26PCX	365	416	49.0/-51.5	0.36	1.78 × 10^1^	8.45 × 10^4^	3.50 × 10^3^	3.81 × 10^3^
	(388)^a^	(470)^a^		(0.12)^a^	(1.1 × 10^7^)^b^			(8.0 × 10^5^)^b^
36PCX	358	410	49.5/-49.4	0.45	4.21 × 10^3^	6.13 × 10^9^	2.00 × 10^1^	1.34 × 10^3^
	(393)^a^	(440)^a^		(0.16)^a^	(2.8 × 10^7^)^b^			(4.3 × 10^5^)^b^

a Measured in toluene (10^–5^ M);

b Values obtained from the PPF: 20 wt% dopant films (Zhang et al., 2022). The dihedral angle is chosen in the optimized structure of the ground state; The adiabatic excitation energy between S_1_ and T_1_ states is calculated by MN15/cc-pVDZ.

#### 3.1.2 Photophysical process

We further evaluate photophysical properties in PCX series to understand the void-carbon effect on their emission. The radiative decay constant *k*_r_ (1.80 × 10^6^ s^−1^) is the largest in 27PCX among PCX series, however, the rate *k*_RISC_ is the lowest. In terms of the reverse intersystem crossing process, it is expected that 27PCX molecule should have a low photoluminescence quantum yield, which can not be used as a high-efficient TADF molecule. Among the other molecules, the radiation rate in 36PCX is 4.21 × 10^3^ s^−1^, larger than the radiation rate of 1.78 × 10^1^ s^−1^ in 26PCX, nevertheless *k*_RISC_ in 36PCX (1.34 × 10^3^ s^−1^) is slightly smaller than that in 26PCX (3.81 × 10^3^ s^−1^). Comparing with the experimental data of the literature in Table 1, the *k*_r_ in 36PCX is 2.8 × 10^7^ s^−1^, greater than *k*_r_ of 1.1 × 10^7^ s^−1^ in 26PCX, whereas *k*_RISC_ in 36PCX (4.3 × 10^3^ s^−1^) is slightly smaller than that in 26PCX (8.0 × 10^3^ s^−1^) (Zhang et al., 2022). The experimental and calculated results differ by several orders of magnitude, but follow similar trends, which can be attributed to the fact that the calculations are performed in the gas state and experiments are measured in the dopant films (Hu et al., 2021). It indicates that we have provided a reliable prediction in TADF emission properties for PCX series by the comparison between our simulations and experimental spectra.

As mentioned above, it is found that the position of the void-carbon and molecular conjugation can be adjusted synergistically to tune the excited-state character and photophysical properties in D-A TADF. However, the universal applicability of the void-carbon should be tested on a wide range of systems. Considering the effect of donor and acceptor fragments on the emission, we propose to investigate a few series of molecules with 9,9-dimethylacridin-10-yl (DmAc) and 3,9′-Bi-9H-carbazole (BiCz) as donors and phenoxathiin,10,10-dioxide (Pd) and dibenzothiophene-S,S-dioxide (Dd) as acceptors respectively in the following sections. We aim at designing a universal rule for improving the emission properties in D-A TADF molecules.

### 3.2 Donor fragment effect

The overlap of frontier molecular orbitals and charge transfer is affected by the delocalization in D-A TADF (Sun et al., 2020). Delocalization of HOMO in PhCz, DmAc, and BiCz are listed in Supplementary Table S4 of the Supplementary Material, which shows that three donor fragments have a similar degree of delocalization. We select them to test the applicability of the void-carbon strategy in different donor fragments.

#### 3.2.1 DAX series

**FIGURE 4:**
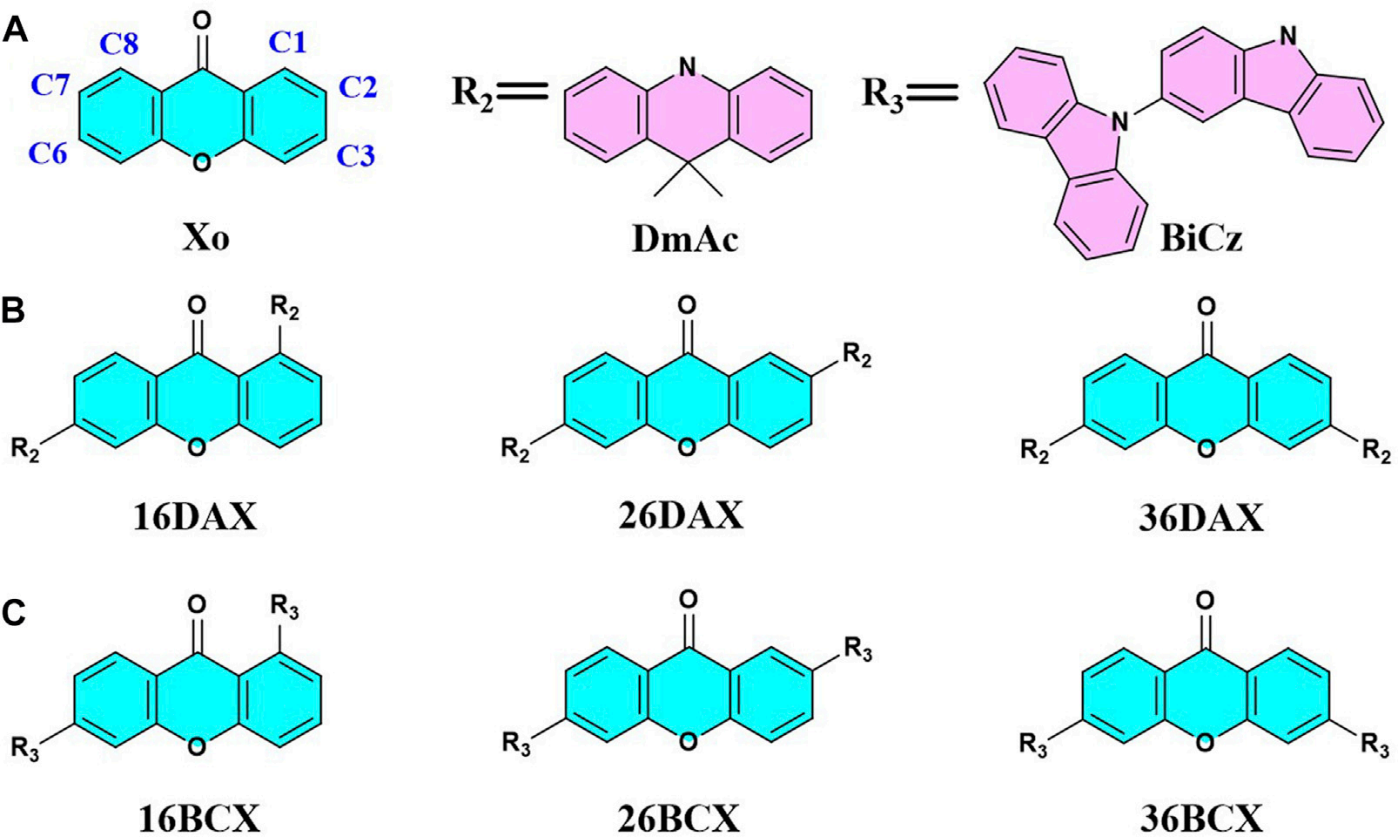
Structure diagram of the Xo, DmAc, BiCz **(A)**, DAX **(B)**, and BCX **(C)** molecules investigated.

**FIGURE 5:**
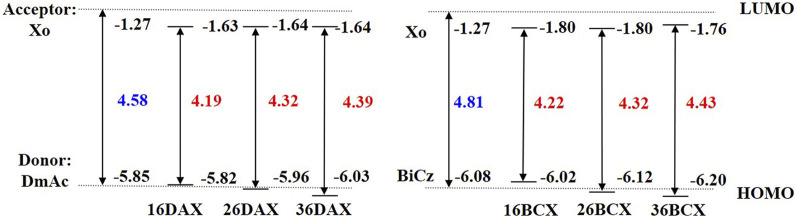
Comparison of HOMO-LUMO energy gaps between DAX and BCX series. The dotted lines represent the energy levels of LUMO for Xo and HOMO for DmAc and BiCz respectively.

We investigate the donor effect on the emission of D-A TADF in DAX series, which have DmAc donor fragments in different connection positions with the same acceptor Xo fragment. Similar to the above PCX series, three molecules are denoted as 16DAX, 26DAX, and 36DAX, presented in Figure 4B. The frontier molecular orbitals and excitation energies are evaluated using DFT and TDDFT calculations, as shown in Supplementary Figure S3, Supplementary Table S5, and Figure 5.

As the void-carbon C3 connects the donor with acceptor fragments in 36DAX, it has the largest HOMO-LUMO gap of 4.39 eV among three DAX molecules (Figure 5). Additionally, based on the adiabatic excitation energies, we observe that the wavelength of the emission spectra blue shifts from 611 to 452 nm among DAXs, in alignment with the trend in the energy gap of the frontier molecular orbitals (Supplementary Table S5). Considering the solvation effect, the emission wavelength of 36DAX is 555 nm measured in THF solution (Chen et al., 2021), which is about 100 nm red-shift compared to our computational simulations in the gas phase. It arises from another fact that the MN15 functional overestimates the excitation energy regardless of the solvation effect. Emission is red-shifted by 42 nm from 36PCX to 36DAX by replacing the donor fragment, due to the decrease in the molecular conjugation observed from dihedral angles (Supplementary Table S5). The energy difference between singlet and triplet states, Δ*E*_ST_ in 16DAX, 26DAX, and 36DAX increases from 0.02, 0.05, to 0.15 eV, indicating that 36DAX should be a promising D-A TADT candidate. Computational simulations demonstrate that similar to the PCX series, the void-carbon strategy works in the DAX series as well.

#### 3.2.2 BCX series

BiCz is applied as the donor fragment in the BCX series, with the structure of 16BCX, 26BCX, and 36BCX shown in Figure 4C. Compared to the emission spectra and frontier molecular orbitals gap (Supplementary Table S5 and Figure 5), the emission wavelengths of BCX series are blue-shifted from 526 to 431 nm due to the increase of the HOMO-LUMO gap. It demonstrates that the void-carbon strategy works in BCX series, as the presence of void-carbon in 36BCX results in a blue-shifted emission. In regard to the steric hindrance, it shows a negligible effect on the conjugation of molecules. Although Δ*E*_ST_ in all BCXs is less than 0.5 eV, the SOC of 36BCX is significantly small (0.03 cm^−1^, Supplementary Table S6), which may prohibit the delayed fluorescence in 36BCX. Accordingly, it shows that *k*_RISC_ of 36BCX is almost 0 s^−1^, when we compare it with calculated properties in other BCXs, demonstrating that 36BCX does not provide delayed fluorescence. The dipole moment in 36BCX in the ground state is 3.1 Debye, which might have a strong effect in the excited state in a polar solvent. Since we have not considered the solvation environment in the calculations, 36BCX might be applied as an effective emitter in a polar environment.

In this section, we reveal that the energy gap, Δ*E*_ST_, and photophysical properties could be tuned by the position of the donor fragment, however, different acceptors may also modify emission properties in D-A TADF. Next, we replace the acceptor Xo with two other fragments with different aromaticity to corroborate the void-carbon strategy.

### 3.3 Acceptor fragment effect

**FIGURE 6:**
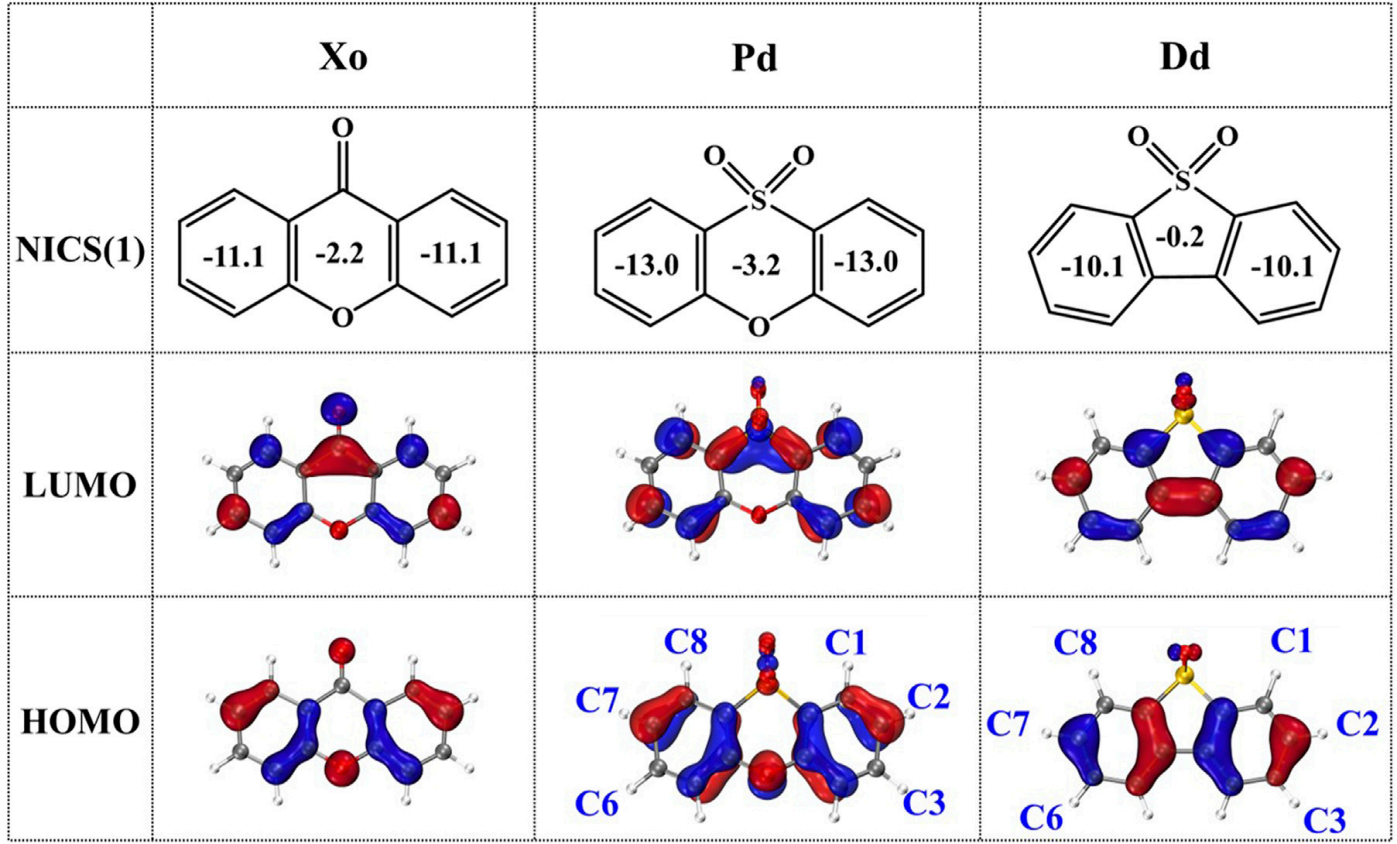
NICS(1) values and frontier molecular orbitals for the studied Xo, Pd, and Dd molecules.

NICS(1) is defined as the negative value of the magnetic shielding at 1 Å above the aromatic ring. We use NICS(1) to evaluate the aromatic properties of selected acceptors, Xo, Pd, and Dd, since it provides better interpretation in the aromaticity rather than NICS(0) (Dobrowolski and Lipinski, 2016). The NICS(1) values are depicted in Figure 6, which indicates that both Xo and Pd are aromatic, while Dd exhibits antiaromaticity. The aromaticity of molecules can affect the excited state properties, therefore we compare different acceptor fragments with different aromaticity. Moreover, the change in the void-carbon positions leads to the difference in the phase of molecular orbitals. As shown in Figure 6, the HOMOs of Xo and Pd are symmetric along their principal axes (C2 axis in the C2v point group) when C3 is the void carbon, but it is antisymmetric for Dd when the void position is at C1. On the other hand, the LUMO of Dd is symmetric along the long axis of the molecule (C2-C7 direction), while the LUMOs of Xo and Pd are antisymmetric. It indicates that the position of the void-carbon may change the molecular orbitals in D-A molecules.

#### 3.3.1 PCP series

**FIGURE 7:**
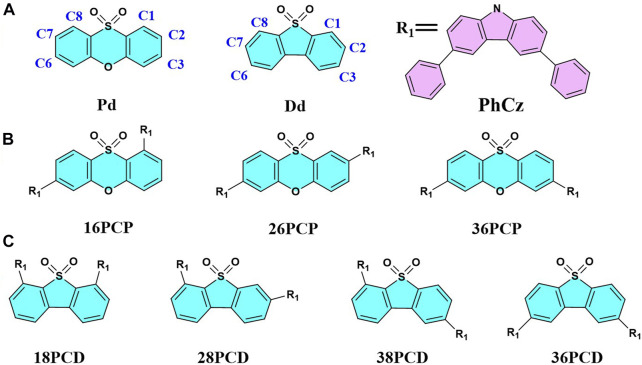
Structure diagram of the Pd, Dd, PhCz **(A)**, PCP **(B)**, and PCD **(C)** molecules investigated.

**FIGURE 8:**
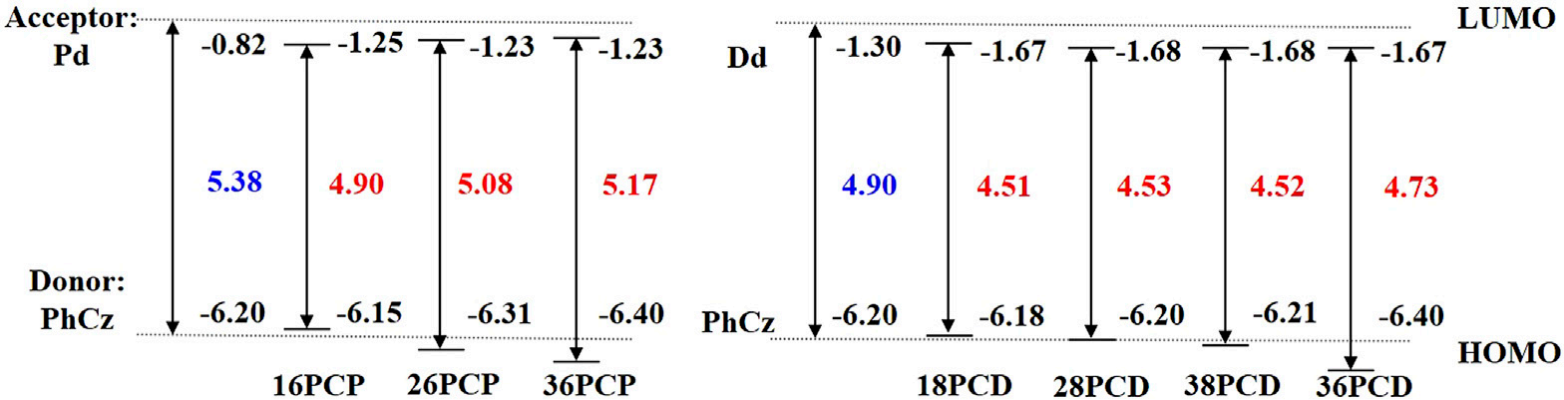
Comparison of HOMO-LUMO energy gaps between PCP and PCD series. The dotted lines represent the energy levels of HOMO for PhCz and LUMO for Pd and Dd respectively.

The distribution of HOMO in Pd is similar to that of Xo as shown in Figure 6, demonstrating that both C3 and C6 are void-carbon atoms. According to the void-carbon strategy, we propose PCP series as 16PCP, 26PCP, and 36PCP as studied systems, in which PhCz fragments connect to acceptor Pd at different positions (Figure 7B). As shown in Figure 8 and Supplementary Table S7, the comparison between PCP and PCX series reveals that the void-carbon atom has a similar effect on HOMO-LUMO gap and molecular conjugation with blue-shifted emission from 16PCP to 36PCP. 36PCP is blue-shifted by 139 nm and 100 nm relative to 16PCP and 26PCP respectively, caused by a wide energy gap (5.17 eV) in 36PCP. Even dihedral angles of 26PCP are similar to that of 36PCP, blue-shifted emission in 36PCP comes from the void-carbon atom in the C3 location. The difference in the emission spectra between 16PCP and 26PCP is mainly attributed to conjugation between donor and acceptor fragments. In the PCP series, 16PCP, and 36PCP are promising in TADF by evaluating Δ*E*_ST_, which have an energy difference between singlet and triplet states less than 0.5 eV. However, Δ*E*_ST_ in 26PCP is 0.74 eV, which is too large for a fast reverse intersystem crossing. Considering our calculations in the newly designed PCP series, both void-carbon strategy and steric hindrance influence their emission behavior.

#### 3.3.2 PCD series

Dd is antiaromatic in contrast to the aromatic acceptor fragments Xo and Pd, according to NICS(1) evaluations. To investigate the effect of the void-carbon on the antiaromaticity acceptor, 18PCD, 28PCD, 38PCD, and 36PCD are investigated in this subsection, as shown in Figure 7C. According to the void-carbon strategy, we expect that the energy gap of 18PCD should be the largest and the emission wavelength should be the smallest among PCD series, since C1 and C8 are the void positions. Conversely, 18PCD has the smallest HOMO-LUMO gap with red-shifted emission compared to the other PCDs. It shows that the energy gap and emission wavelength of 18PCD are 4.51 eV and 504 nm, respectively in Figure 8 and Supplementary Table S7. A blue-shifted emission at 417 nm is observed in 36PCD, which violates the void-carbon rule discussed above. There should be another factor that determines the emission properties in the PCD series, rather than the straightforward void-carbon factor. We notice that steric hindrance is reduced when the connecting positions are at C3 and C6 atom, leading to a conjugated isomer with a smaller dihedral angle of 50.0/-50.6° in 36PCD as shown in Supplementary Table S7. It demonstrates that the conjugation impacts the emission properties in PCDs. When the conjugation is small in 18PCD with a large dihedral angle, a small Δ*E*_ST_ is obtained in facilitating TADF. The charge distribution is different in antiaromatic acceptor Dd, resulting in different excited-state properties in comparison with aromatic acceptor-based molecules.

**FIGURE 9:**
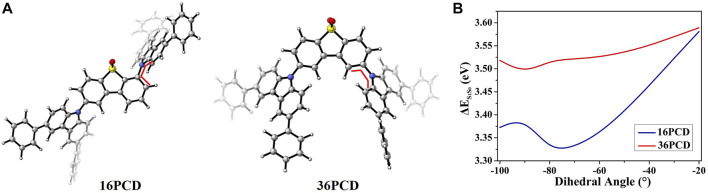
**(A)** Molecular structures of 16PCD and 36PCD and the positions of scanning dihedral angles; **(B)** Effect of dihedral angle variation on excitation energy.

The radiative constant *k*_r_ increases from 18PCD to 36PCD, reaching the maximum of 5.31 × 10^5^ s^−1^ in 36PCD, which implies that improvement in the molecular conjugation increases radiation rate constant rapidly. However, *k*_RISC_ is almost 0 s^−1^ in PCD series, because charge transfer between antiaromatic acceptor and donor fragments is hindered (Supplementary Table S6). To cast the effect of the conjugation on RISC, we carry out simulations in potential energy surface along the rotation between donor and acceptor fragment in 16PCD and 36PCD. As it shows in Figure 9, firstly, the energy difference between singlet state and ground state (Δ*E*_S1S0_) in 36PCD is larger than that in 16PCD in all conformers when dihedral angle varies from -20 to -100°. It is caused by the reduction in the overlap of molecular orbitals in the twisted conformer. Due to the wider energy gap between S_0_ and S_1_ states in 36PCD, it is expected that its emission should be blue-shifted compared to that in 16PCD. Secondly, Δ*E*_S1S0_ along the potential energy surface increases when the dihedral angle varies from -90 to -20° in 36PCD, indicating that the planar conformer should have blue-shifted emission. Comparing the emission properties in the above D-A TADFs, the void-carbon strategy only works for aromatic acceptors, while the steric hindrance effect plays an important role in antiaromatic acceptors.

## 4 Discussion and conclusion

We have investigated the feasibility and applicability of the void-carbon strategy *via* computational simulations in this study. In the PCX series, the HOMO-LUMO gap is wider in a more planar system when the void-carbon is introduced, resulting in blue-shifted emission. Among PCX series, computational simulations demonstrate that 36PCX improves photoluminescence behavior in TADF. However, the discrepancy in the radiative decay rate compared with experiments is due to the lack of consideration of the solvation environment, which should be improved by an explicit solvent model. Regarding the role of void-carbon in emission, it is found that emission properties in DAX and BCX series are consistent with that in PCXs when the donor fragment is replaced by different groups. Furthermore, we find that wavelength in the emission is determined by the eigenvalue of HOMO in donor groups. The energy level of HOMO is positively correlated with the emission wavelength of D-A type molecules when the D-A linkage is at the same position. In addition, the aromaticity of the acceptor fragment influences the properties of D-A TADF. When the acceptor is an aromatic fragment, the properties of these molecules are similar to PCX series, that is, the rule of void-carbon is justified. In contrast, the void-carbon rule does not work when the acceptor is antiaromatic. With regard to the photophysical properties of TADF, we find that the emission properties are determined mainly by the steric effect instead of the position of the void-carbon.

To demonstrate emission properties, we could compare the character between CT and locally-excited (LE) states by the natural transition orbital (NTO), as it provides insightful information for the RISC process in TADF molecules (Cai and Su, 2018). As shown in Supplementary Figures S7, S8, it is found that charge transfer dominates S_0_ → S_1_ transition in PCX series. However, S_0_ → T_1_ transition in 36PCX is different, in which the mixture between CT and LE are detected, resulting in enhancing RISC process according to El-Sayed’s Rule (El-Sayed, 1963). Additionally, in BCX series, the NTO of 36BCX is similar to that of 36PCX, which has RISC enhancement effect as well. In PCP series, the NTO of S_0_ → T_1_ transition shows that the mixed CT and LE states in 36PCP are beneficial to the generation of RISC. Finally, in PCD series, we notice that the locally-excited state dominates the S_0_ → T_1_ transition, while CT is only detected in the S_0_ → S_1_ transition, caused by the change in the aromaticity of the acceptor. According to the analysis in NTOs, we propose that 36PCX, 36BCX, and 36PCD are the emitters with the best performance in deep-blue emission among our selected series. Our simulations show that the photophysical properties of D-A TADF are determined by the void-carbon strategy when the acceptor group is aromatic. In this study, we provide an insightful guideline for the preparation of high-performance TADF molecules with blue-emission.

## Data Availability

The original contributions presented in the study are included in the article/Supplementary Material, further inquiries can be directed to the corresponding authors.
