# Supplementary material for: Effect of void-carbon on blue-shifted luminescence in TADF molecules by theoretical simulations
Source: Front Chem. 2023 Jan 26;11:1094574. doi: 10.3389/fchem.2023.1094574 (PMC9908751; doi:10.3389/fchem.2023.1094574)
Supplement: Supplementary file 1 [file DataSheet1.pdf]

1 SUPPLEMENTARY FIGURES AND TABLES

1.1 Supplementary Figures

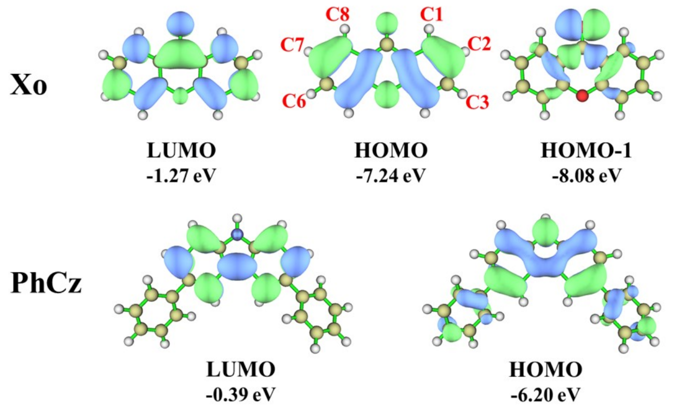

Figure S1. The HOMO, HOMO-1, and LUMO of Xo fragment; HOMO and LUMO of PhCz fragment.

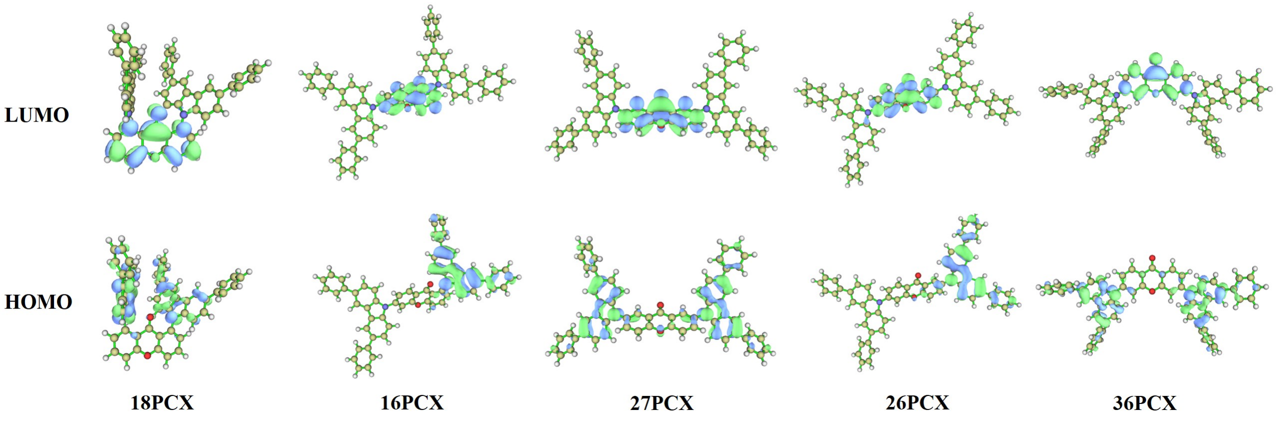

Figure S2. The HOMO and LUMO of PCX series.

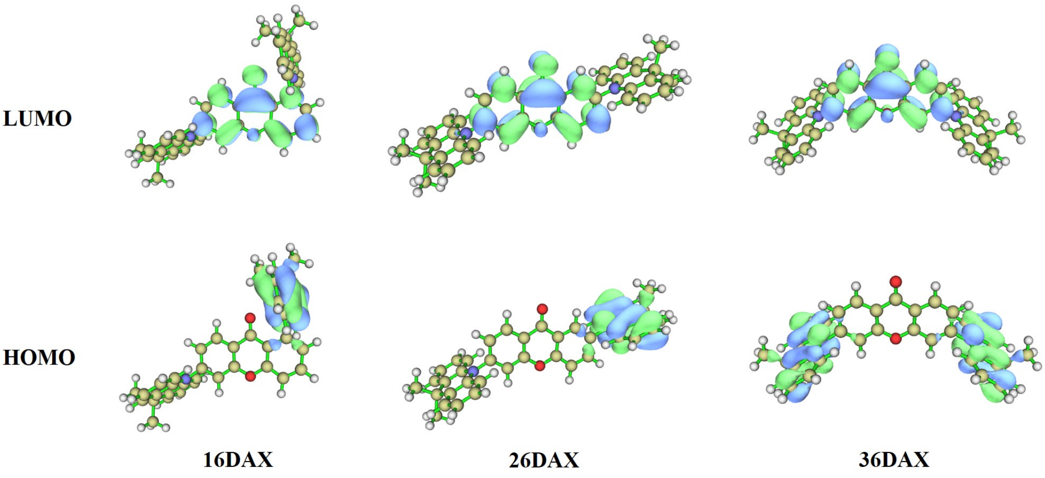

Figure S3. The HOMO and LUMO of DAX series.

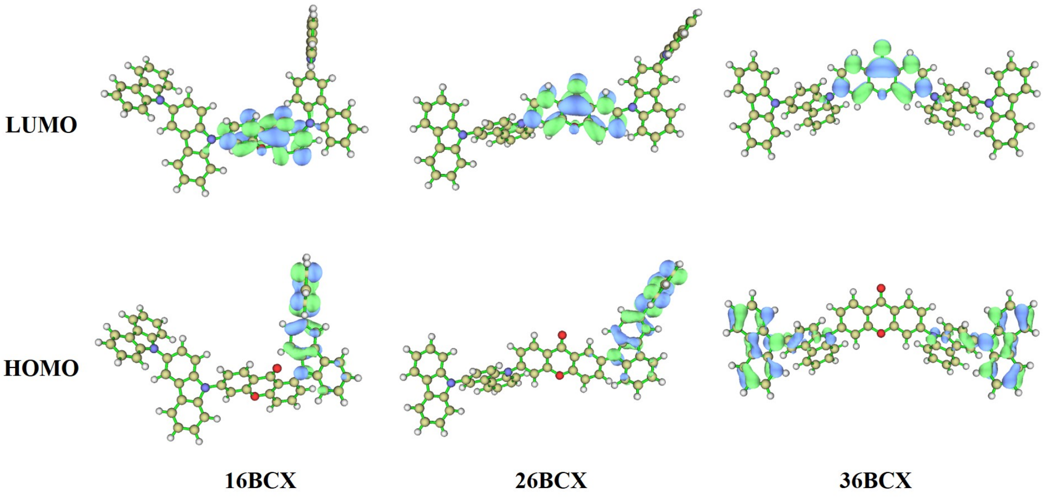

Figure S4. The HOMO and LUMO of BCX series.

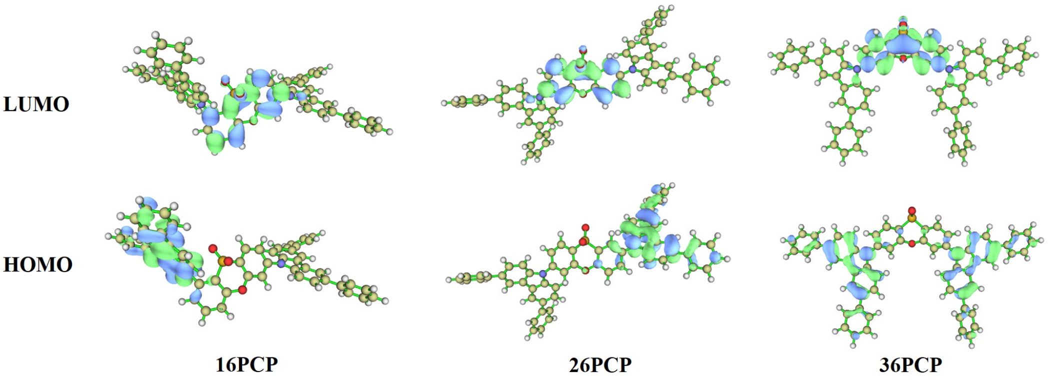

Figure S5. The HOMO and LUMO of PCP series.

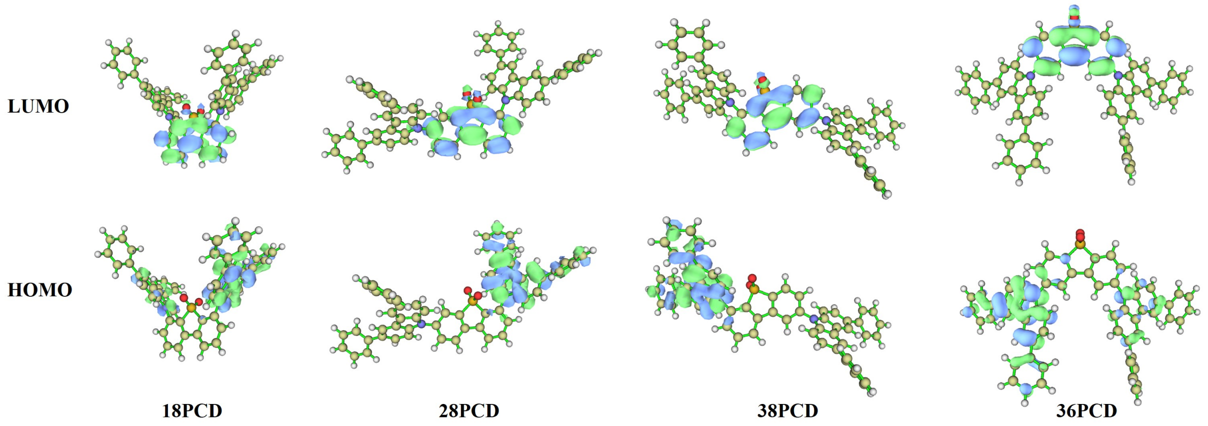

Figure S6. The HOMO and LUMO of PCD series.

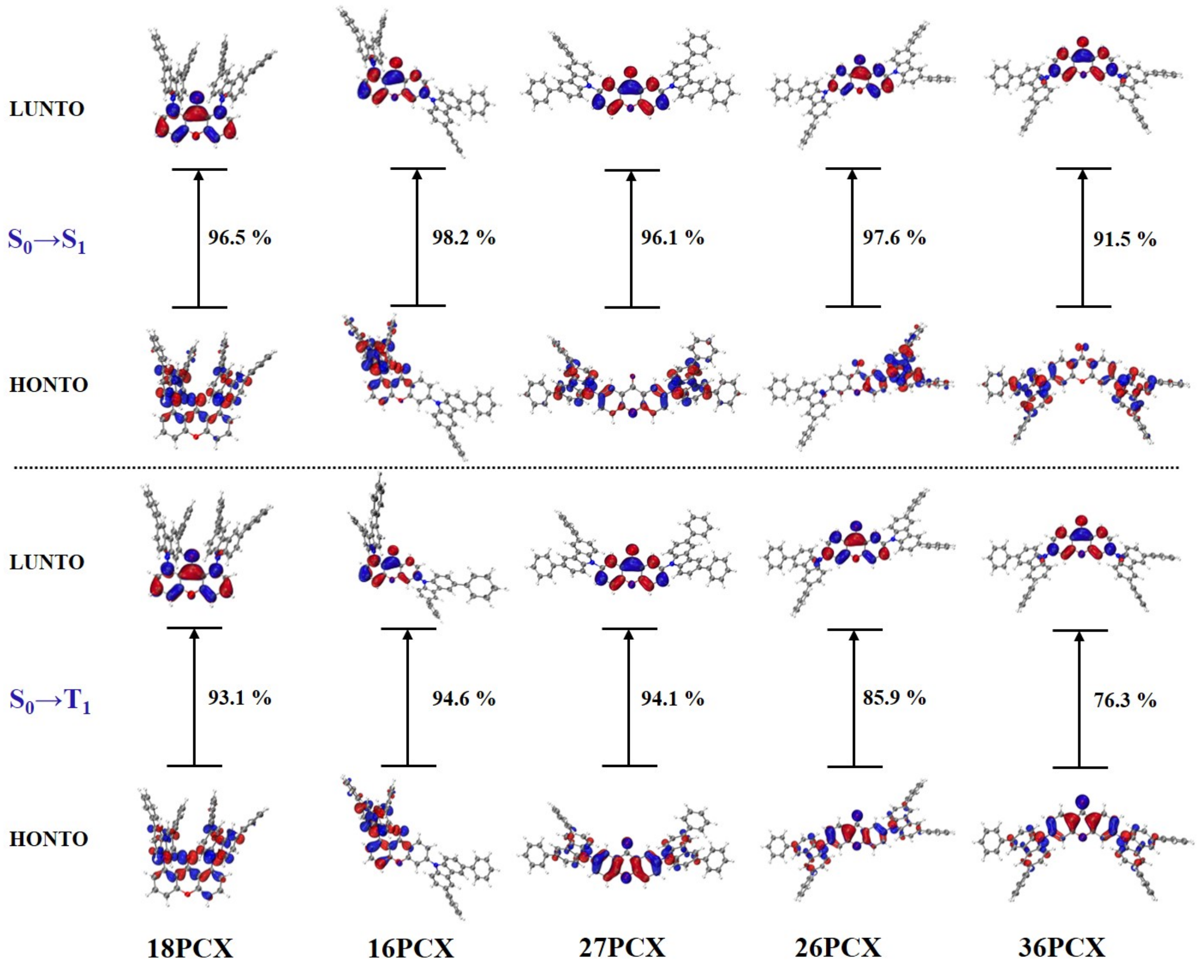

Figure S7. The NTOs of PCX series for $S_0 \rightarrow S_1$ and $S_0 \rightarrow T_1$ transitions.

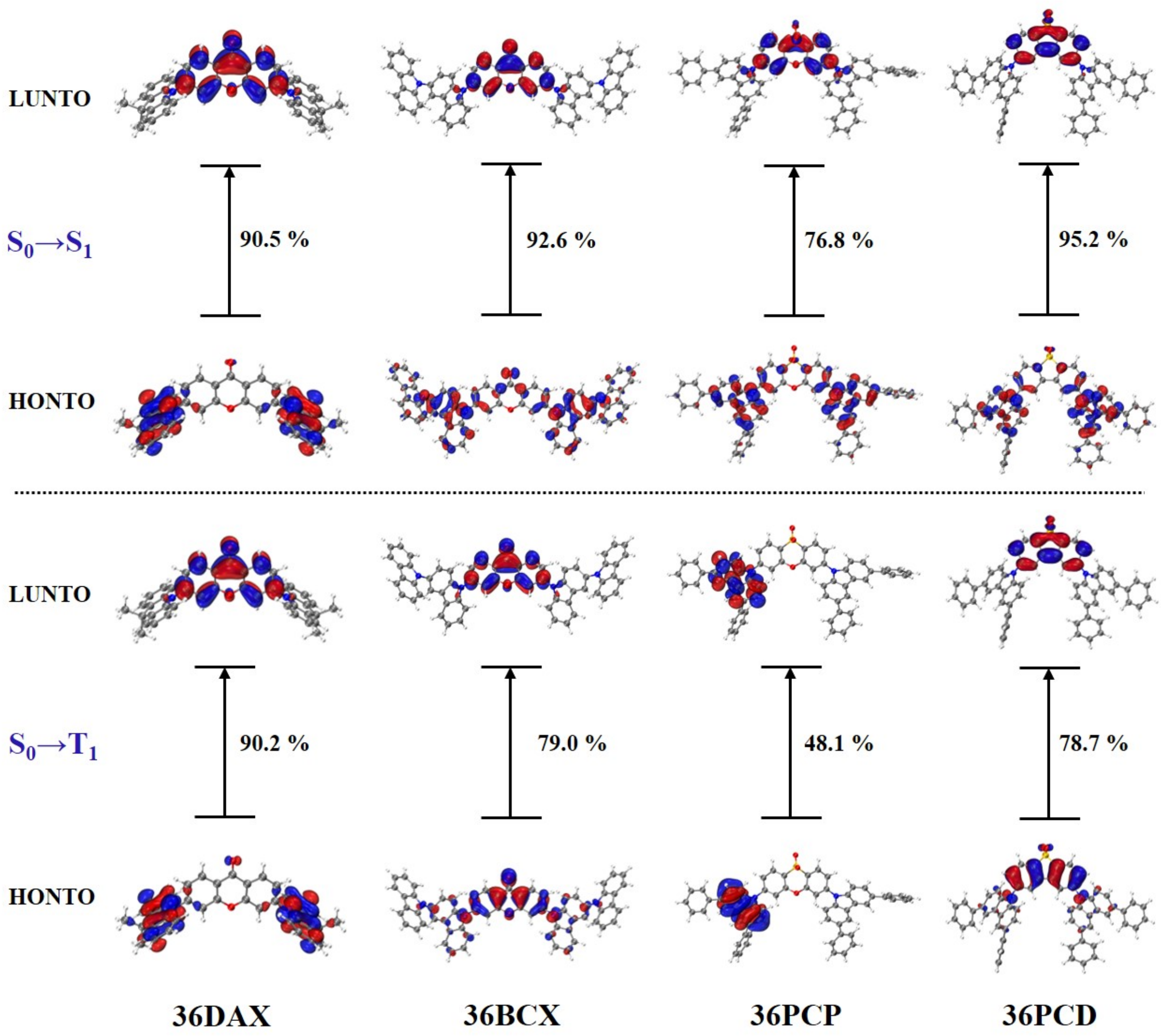

Figure S8. The NTOs of 36DAX, 36BCX, 36PCP, and 36PCD for $S_0 \rightarrow S_1$ and $S_0 \rightarrow T_1$ transitions.

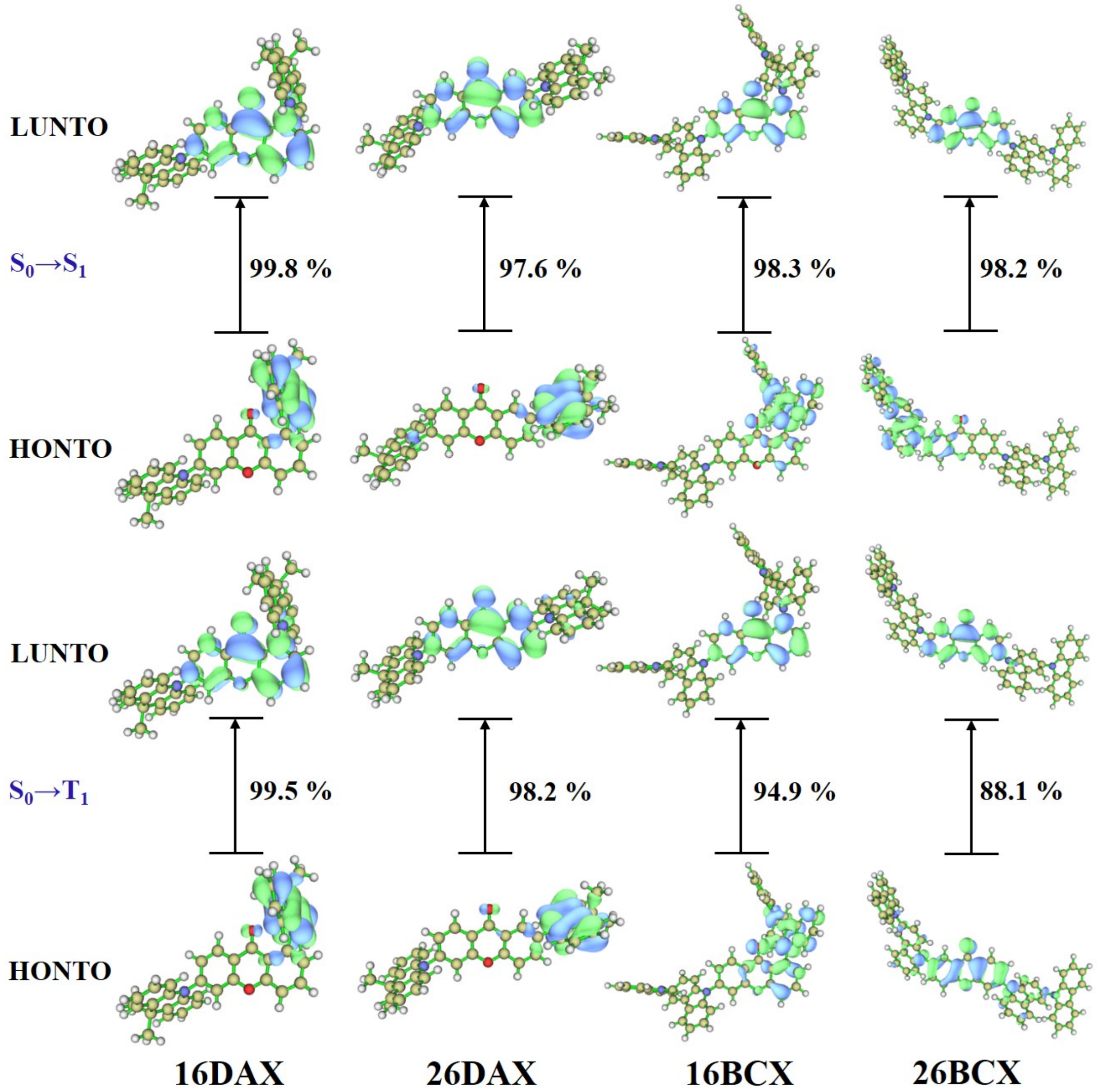

Figure S9. The NTOs of 16DAX, 26DAX, 16BCX, and 26BCX for $S_0 \rightarrow S_1$ and $S_0 \rightarrow T_1$.

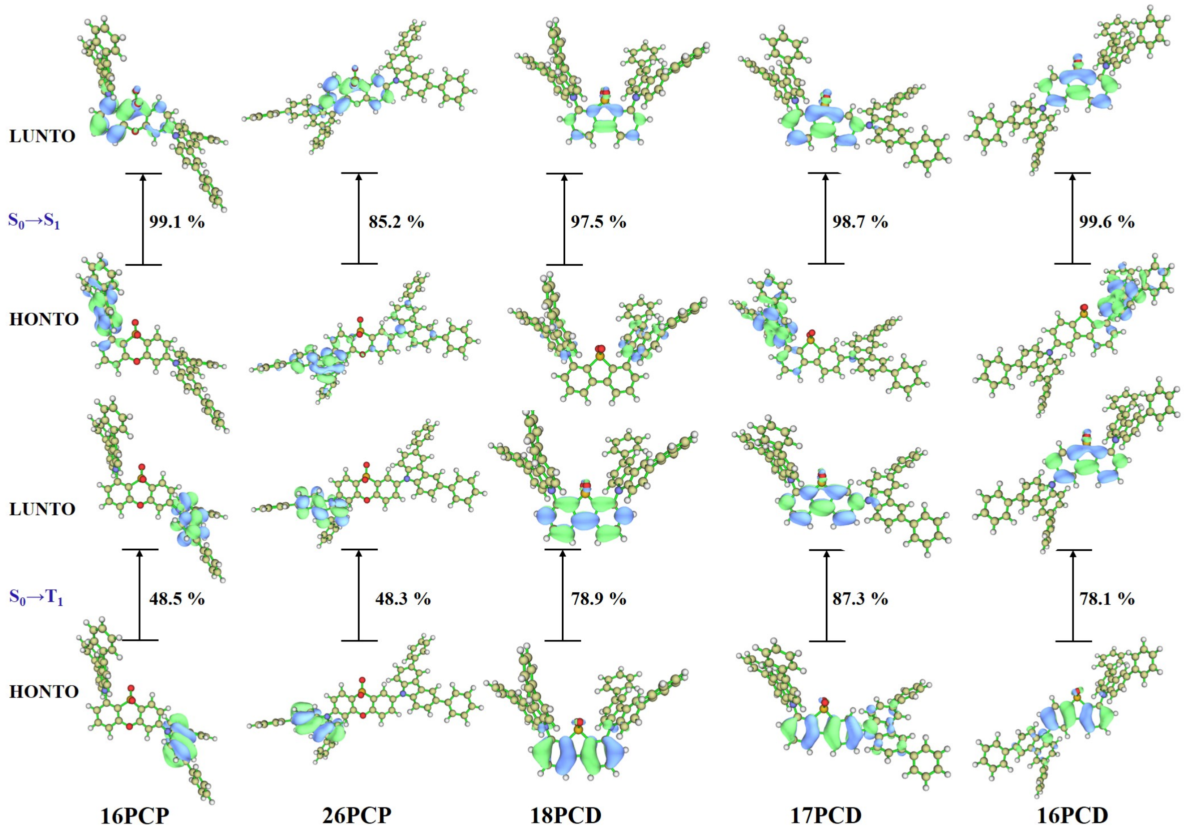

Figure S10. The NTOs of 16PCP, 26PCP, 18PCD, 17PCD, and 16PCD for $S_0 \rightarrow S_1$ and $S_0 \rightarrow T_1$.

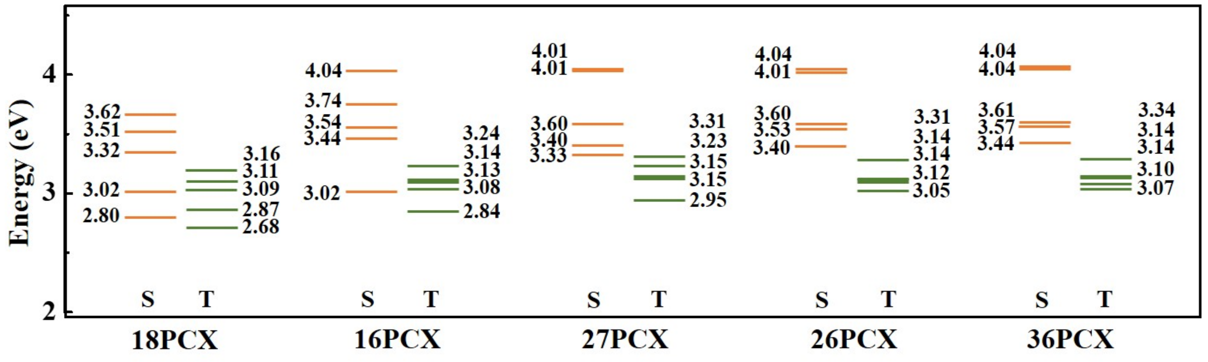

Figure S11. The energy level diagram of PCX series.

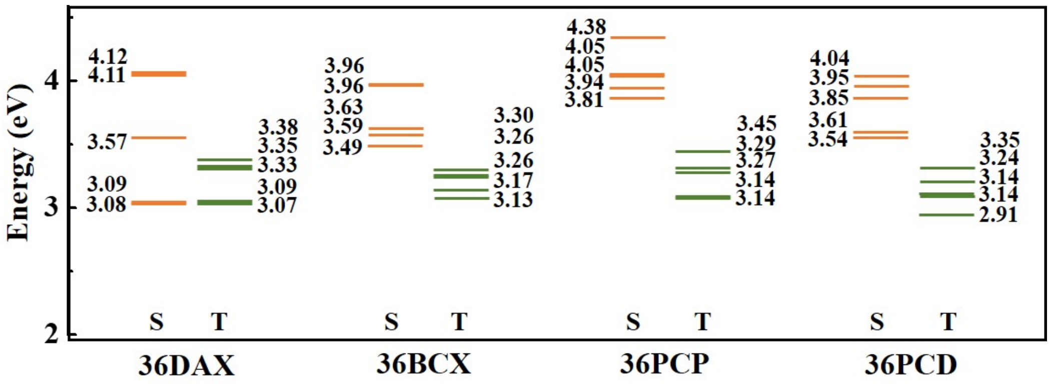

Figure S12. The energy level diagram of 36DAX, 36BCX, 36PCP, and 36PCD.

1.2 Supplementary Tables

Table S1. Hartree-Fock composition in different Functional. The absorption emission wavelength of 36PCX at different functional by the optimum structure 36PCX in B3LYP/cc-pVDZ level.

36PCX	HF (%)	$S_0 \rightarrow S_1$ (nm)	$S_1 \rightarrow S_0$ (nm)
B3LYP	20	440	560
MN15	44	360	414
PBE0	25	414	513
M06-2X	54	337	375
WB97XD	22.2 ($\omega < 0.2$)	318	340
	100 ($\omega > 0.2$)		
CAM-B3LYP	19 ($\omega < 0.33$)	328	359
	65 ($\omega > 0.33$)		
Exp	/	393	440

Table S2. The absorption emission wavelength of 36PCX at different functional.

36PCX	S0→S1 (nm)	S0→Sn (nm)	S1→S0 (nm)
B3LYP	440	/	560
PBE0	410	275	501
MN15	358	267	410
exp	393	295	440

Table S3. The SOC of PCX series molecules.

Emitters	18PCX	16PCX	27PCX	26PCX	36PCX
SOC (cm^{-1})	0.22	0.10	0.72	0.73	0.40

Table S4. The ODI values of three donors molecules.

	PhCz (%)	DmAc (%)	BiCz (%)
ODI (H)	5.91	9.55	6.45
ODI (L)	7.74	6.95	8.03

Table S5. The absorption emission wavelength, ΔE_{ST}, and dihedral angles of DAX and BCX series molecules.

	16DAX	26DAX	36DAX	16BCX	26BCX	36BCX
Abs (nm)	475	402	403	404	362	355
Emi (nm)	611	471	452	526	439	431
ΔE_{ST} (eV)	0.02	0.05	0.15	0.07	0.28	0.20
Dihedral (°)	90.8/-100.0	91.0/-91.0	91.2/-88.4	49.6/-58.0	-49.9-53.1	50.3/-50.3

Table S6. The SOC and photophysical properties of BCX and PCD series molecules.

	16BCX	26BCX	36BCX	18PCD	28PCD	38PCD	36PCD
SOC (cm^{-1})	0.19	0.40	0.03	0.11	0.09	0.11	0.63
k_{r} (s^{-1})	6.51×10^{2}	3.74×10^{5}	2.31×10^{1}	4.03×10^{3}	9.39×10^{3}	9.33×10^{3}	5.31×10^{5}
k_{nr} (s^{-1})	7.23×10^{10}	6.68×10^{10}	2.05×10^{9}	5.22×10^{10}	7.26×10^{10}	5.83×10^{10}	1.53×10^{11}
k_{ISC} (s^{-1})	6.42×10^{3}	7.34×10^{5}	3.56×10^{-4}	1.51×10^{2}	3.20×10^{3}	8.43×10^{3}	4.56×10^{6}
k_{RISC} (s^{-1})	6.77×10^{2}	3.55×10^{1}	1.94×10^{-1}	1.02×10^{-2}	4.13×10^{-5}	3.95×10^{-1}	2.82×10^{-4}

Table S7. The absorption emission wavelength, ΔE_{ST}, and dihedral angles of PCP and PCD series molecules.

	16PCP	26PCP	36PCP	18PCD	28PCD	38PCD	36PCD
Abs (nm)	347	410	372	371	468	369	351
Emi (nm)	559	520	420	504	497	496	417
ΔE_{ST} (eV)	0.13	0.74	0.39	0.28	0.48	0.33	0.70
Dihedral (°)	-49.6/-64.8	-49.8/50.3	-50.3/49.1	-57.7/-57.8	-58.0/-50.2	-59.0/-51.4	50.0/-50.6

1.3 Coordinates of all molecules

Table S8: Coordinates of the optimized minimum in the ground state of donor at the MN15/cc-pVDZ level.

PhCz				BiCz				DmAc			
C	-6.26000	-1.49900	0.65200	C	2.47800	-1.18300	-1.02200	C	-3.71300	-0.24100	-0.00100
C	-6.06000	-2.67000	-0.08300	C	2.20500	-0.17600	-0.06300	C	-2.52800	-0.97600	-0.00100
C	-4.87600	-2.83100	-0.80600	C	3.48800	0.28100	0.43700	C	-1.26600	-0.36800	-0.00000
C	-3.90400	-1.83100	-0.79600	C	4.47700	-0.47000	-0.24400	C	-1.22000	1.03800	0.00000
C	-4.09000	-0.65000	-0.05900	C	1.45000	-1.84300	-1.70400	C	-2.40900	1.78700	0.00000
C	-5.28500	-0.50200	0.66500	C	0.14000	-1.47100	-1.42100	C	-3.64700	1.15400	-0.00000
C	-3.42100	1.77600	-0.04700	C	-0.14800	-0.48000	-0.46000	C	-0.00000	-1.22900	0.00000
C	-3.05000	0.41100	-0.04400	C	0.87900	0.16600	0.22600	C	1.22000	1.03800	-0.00000
C	-1.69100	0.07800	-0.02800	C	5.84100	-0.28000	0.00200	C	1.26600	-0.36800	0.00000
C	-0.72600	1.09000	-0.01400	C	6.19900	0.68000	0.94500	C	2.52800	-0.97600	-0.00000
C	-1.12900	2.44700	-0.01700	C	5.22800	1.43500	1.63000	C	3.71400	-0.24100	-0.00000
C	-2.48200	2.80100	-0.03400	C	3.87300	1.24100	1.38200	C	3.64700	1.15400	-0.00000
N	0.00000	3.24700	-0.00000	C	-2.49700	-1.01500	0.22900	C	2.40900	1.78700	-0.00000
C	0.72600	1.09000	0.01400	C	-3.71200	-0.30500	0.37500	C	0.00000	-2.12200	-1.25500
C	1.69100	0.07800	0.02800	C	-3.43300	1.07300	0.01900	C	-0.00000	-2.12000	1.25700
C	3.05000	0.41100	0.04400	C	-2.06300	1.13300	-0.32700	N	0.00000	1.69600	0.00000
C	3.42100	1.77600	0.04700	C	-4.86300	-0.97800	0.80400	H	-4.67700	-0.75300	-0.00100
C	2.48200	2.80100	0.03400	C	-4.78400	-2.33900	1.08500	H	-2.58400	-2.06800	-0.00000
C	1.12900	2.44700	0.01700	C	-3.56500	-3.02900	0.94600	H	-2.34500	2.87900	0.00100
C	4.87600	-2.83200	0.80600	C	-2.40700	-2.38200	0.52000	H	-4.56000	1.75300	-0.00000
C	6.06000	-2.67000	0.08300	C	-1.46300	2.32600	-0.74700	H	2.58400	-2.06800	-0.00000
C	6.26000	-1.49900	-0.65200	C	-2.26300	3.46600	-0.80000	H	4.67700	-0.75300	-0.00100
C	5.28500	-0.50100	-0.66400	C	-3.62500	3.42600	-0.44800	H	4.56000	1.75300	-0.00000
C	4.09000	-0.65000	0.05900	C	-4.21600	2.23300	-0.04100	H	2.34500	2.87900	0.00000
C	3.90400	-1.83200	0.79600	N	-1.50300	-0.13700	-0.19700	H	0.88900	-2.77000	-1.27300
H	-7.17700	-1.36500	1.22900	N	3.84900	-1.34500	-1.11600	H	0.00000	-1.50800	-2.16800
H	-6.82200	-3.45200	-0.09200	H	1.66300	-2.61400	-2.44600	H	-0.88900	-2.77000	-1.27300
H	-4.71200	-3.73800	-1.39200	H	-0.69300	-1.94400	-1.94500	H	0.88900	-2.76900	1.27500
H	-2.99400	-1.95200	-1.38800	H	0.63800	0.92300	0.97600	H	-0.89000	-2.76900	1.27500
H	-5.43700	0.39800	1.26600	H	6.59800	-0.86300	-0.52700	H	-0.00000	-1.50600	2.16900
H	-4.48300	2.03000	-0.08600	H	7.25600	0.84900	1.15600	H	0.00000	2.70900	0.00000
H	-1.38600	-0.97100	-0.00000	H	5.54500	2.17900	2.36300				
H	-2.79500	3.84700	-0.04700	H	3.11900	1.82600	1.91300				
H	0.00000	4.25900	-0.00000	H	-5.80600	-0.43900	0.91900				
H	1.38600	-0.97200	0.00000	H	-5.67100	-2.87900	1.41900				
H	4.48300	2.03000	0.08700	H	-3.52200	-4.09500	1.17900				
H	2.79500	3.84700	0.04700	H	-1.46100	-2.91600	0.42100				
H	4.71100	-3.73900	1.39100	H	-0.40900	2.35800	-1.02700				
H	6.82200	-3.45200	0.09200	H	-1.82100	4.41000	-1.12500				
H	7.17800	-1.36500	-1.22800	H	-4.22100	4.33900	-0.49900				
H	5.43800	0.39900	-1.26500	H	-5.27500	2.19700	0.22600				
H	2.99300	-1.95300	1.38800	H	4.32000	-1.99800	-1.72900				

Table S9: Coordinates of the optimized minimum in the ground state of acceptor at the MN15/cc-pVDZ level.

Xo				Dd				Pd			
C	-3.66100	0.44000	0.00000	C	1.60800	-2.05000	0.00000	C	-2.28400	-1.88800	0.11100
C	-3.58000	-0.96400	0.00000	C	0.74000	-0.95600	-0.00000	C	-1.18700	-1.03300	0.24300
C	-2.35000	-1.60700	-0.00000	C	1.28300	0.33300	0.00000	C	-1.33400	0.32200	-0.06600
C	-1.17800	-0.83900	-0.00000	C	2.64800	0.58000	-0.00000	C	-2.54300	0.82900	-0.54700
C	-1.23900	0.56100	-0.00000	C	3.50700	-0.52400	-0.00000	C	-3.63300	-0.02600	-0.68300
C	-2.49500	1.19000	0.00000	C	2.98700	-1.82400	0.00000	C	-3.49900	-1.37800	-0.33900
C	0.00000	1.36400	-0.00000	S	0.00000	1.57600	-0.00000	S	-0.00000	1.41400	0.31100
C	1.17800	-0.83900	-0.00000	C	-2.64800	0.58000	0.00000	C	2.54300	0.82900	-0.54700
C	2.35000	-1.60700	-0.00000	C	-1.28300	0.33300	-0.00000	C	1.33400	0.32200	-0.06600
C	3.58000	-0.96400	0.00000	C	-0.74000	-0.95600	-0.00000	C	1.18700	-1.03300	0.24300
C	3.66100	0.44000	0.00000	C	-1.60800	-2.05000	-0.00000	C	2.28400	-1.88800	0.11100
C	2.49500	1.19000	0.00000	C	-2.98700	-1.82400	0.00000	C	3.49900	-1.37800	-0.33900
C	1.23900	0.56100	-0.00000	C	-3.50700	-0.52400	0.00000	C	3.63300	-0.02600	-0.68300
O	-0.00000	-1.52300	-0.00000	O	-0.00000	2.31800	1.26900	O	0.00000	2.55300	-0.62200
O	0.00000	2.58900	-0.00000	O	0.00000	2.31800	-1.26900	O	-0.00000	1.65000	1.76200
H	-4.63500	0.93200	0.00000	H	1.21600	-3.06900	0.00000	O	-0.00000	-1.58500	0.64400
H	-4.49400	-1.56100	-0.00000	H	3.03000	1.60200	0.00000	H	-2.15400	-2.94300	0.35200
H	-2.26300	-2.69400	-0.00000	H	4.58700	-0.37100	-0.00000	H	-2.60000	1.89000	-0.79800
H	-2.50100	2.28200	0.00000	H	3.66900	-2.67500	0.00000	H	-4.58600	0.35600	-1.05200
H	2.26300	-2.69400	-0.00000	H	-3.03000	1.60200	0.00000	H	-4.35200	-2.05100	-0.44200
H	4.49400	-1.56100	0.00000	H	-1.21600	-3.06900	-0.00000	H	2.60000	1.89000	-0.79800
H	4.63500	0.93200	0.00000	H	-3.66900	-2.67600	0.00000	H	2.15400	-2.94400	0.35200
H	2.50100	2.28200	0.00000	H	-4.58700	-0.37100	-0.00000	H	4.35200	-2.05100	-0.44200
								H	4.58600	0.35600	-1.05200

Table S10: Coordinates of the optimized minimum in the S_0, S_1, and T_1 of 18PCX at the MN15/cc-pVDZ level.

S_0				S_1				T_1			
C	8.14000	1.89700	-2.93400	C	1.91800	4.58300	6.26200	C	0.74600	-5.37900	6.06800
C	8.83500	0.71400	-3.19500	C	0.93200	4.48300	7.24600	C	1.76300	-4.98700	6.94200
C	8.41800	-0.47400	-2.59100	C	-0.37700	4.16500	6.87700	C	2.81600	-4.20300	6.46300
C	7.31800	-0.47700	-1.73300	C	-0.69600	3.95000	5.53700	C	2.85100	-3.81600	5.12400
C	6.60900	0.70500	-1.46300	C	0.28500	4.04700	4.53600	C	1.83500	-4.20300	4.23400
C	7.03800	1.89200	-2.07800	C	1.59800	4.36700	4.92200	C	0.78200	-4.99100	4.72900
C	5.20600	1.77800	0.32900	C	0.55900	4.58500	2.09600	C	1.43300	-4.67400	1.79800
C	5.43500	0.69700	-0.55200	C	-0.05500	3.81500	3.11000	C	1.87100	-3.78800	2.80900
C	4.53700	-0.37700	-0.55300	C	-0.98800	2.82700	2.75200	C	2.33600	-2.51400	2.45000
C	3.44000	-0.36700	0.31300	C	-1.29200	2.62100	1.40900	C	2.36000	-2.14000	1.10700
C	3.23900	0.72500	1.18400	C	-0.65700	3.40800	0.41900	C	1.91100	-3.05000	0.12100
C	4.12000	1.81100	1.19900	C	0.27200	4.39800	0.74800	C	1.44400	-4.32400	0.45200
N	2.07100	0.51900	1.92400	N	-1.11400	3.02300	-0.83300	N	2.01200	-2.45500	-1.13000
C	2.35000	-1.29400	0.55400	C	-2.18000	1.70800	0.70200	C	2.76600	-0.93200	0.40200

C	2.06000	-2.56800	0.06000	C	-3.06900	0.71600	1.10300	C	3.29100	0.29400	0.80500
C	0.94900	-3.26700	0.54300	C	-3.82300	0.01600	0.14100	C	3.61500	1.26600	-0.15600
C	0.11900	-2.64200	1.50300	C	-3.66000	0.34800	-1.22300	C	3.41900	0.96700	-1.52300
C	0.38800	-1.37400	2.01000	C	-2.77500	1.33200	-1.65100	C	2.88900	-0.24700	-1.95100
C	1.52400	-0.71000	1.54000	C	-2.03900	2.00500	-0.67300	C	2.55600	-1.18200	-0.97000
C	0.81300	-6.39900	-1.60700	C	-5.53100	-2.70700	2.16600	C	4.33800	4.37100	1.90700
C	0.29800	-7.33800	-0.71000	C	-6.65100	-2.99100	1.38000	C	5.22400	5.06700	1.08100
C	-0.03600	-6.93500	0.58500	C	-6.83200	-2.30700	0.17700	C	5.56900	4.52800	-0.16000
C	0.15700	-5.61200	0.98300	C	-5.91300	-1.33800	-0.22700	C	5.04600	3.29900	-0.56400
C	0.68600	-4.65900	0.09600	C	-4.78300	-1.03800	0.55400	C	4.15700	2.58600	0.25800
C	0.99800	-5.07400	-1.21000	C	-4.60400	-1.75400	1.75200	C	3.80200	3.15300	1.49500
H	8.45000	2.83100	-3.40800	H	2.94700	4.82200	6.54000	H	-0.08700	-5.98400	6.43300
H	9.69600	0.71800	-3.86600	H	1.18200	4.65200	8.29500	H	1.73500	-5.29000	7.99000
H	8.95800	-1.40300	-2.78100	H	-1.15800	4.09300	7.63700	H	3.62100	-3.89800	7.13500
H	7.01500	-1.40500	-1.24200	H	-1.72900	3.73000	5.25300	H	3.69200	-3.22700	4.75100
H	6.48200	2.81600	-1.90300	H	2.38100	4.41900	4.16200	H	-0.03200	-5.27700	4.05900
H	5.91900	2.60500	0.34200	H	1.26300	5.36800	2.38500	H	1.09700	-5.67300	2.08500
H	4.67900	-1.20900	-1.24800	H	-1.44800	2.20700	3.52400	H	2.65200	-1.80900	3.22300
H	3.96500	2.65600	1.87200	H	0.74600	4.99900	-0.02900	H	1.10700	-5.01500	-0.32200
H	2.73400	-3.03600	-0.66200	H	-3.20600	0.50500	2.16600	H	3.47700	0.48800	1.86400
H	-0.77500	-3.16800	1.84300	H	-4.22300	-0.20900	-1.97400	H	3.65700	1.72800	-2.27000
H	-0.27600	-0.91500	2.74500	H	-2.65100	1.57100	-2.70900	H	2.72900	-0.46000	-3.00900
H	1.06600	-6.69700	-2.62700	H	-5.36400	-3.25000	3.09800	H	4.04000	4.79200	2.86900
H	0.15500	-8.37500	-1.01900	H	-7.37400	-3.74400	1.70200	H	5.63700	6.02600	1.40000
H	-0.44400	-7.65700	1.29500	H	-7.70600	-2.51300	-0.44500	H	6.26500	5.05800	-0.81400
H	-0.07800	-5.31700	2.00800	H	-6.10000	-0.77800	-1.14500	H	5.36200	2.86900	-1.51700
H	1.38000	-4.34200	-1.92700	H	-3.71100	-1.58000	2.35600	H	3.07200	2.64600	2.13000
C	-1.84200	4.10000	-6.70000	C	7.79700	-2.14400	2.44500	C	-8.00100	-0.45900	3.16600
C	-2.58100	3.29400	-7.56900	C	7.76500	-3.42000	3.01300	C	-8.35100	0.76800	3.73300
C	-3.40200	2.29300	-7.04400	C	6.73000	-4.29600	2.67600	C	-7.68400	1.92700	3.33100
C	-3.48400	2.10300	-5.66500	C	5.73700	-3.89900	1.78200	C	-6.67600	1.85900	2.36900
C	-2.74600	2.90500	-4.77900	C	5.75600	-2.61900	1.20300	C	-6.31300	0.63200	1.79000
C	-1.92300	3.90700	-5.32100	C	6.80300	-1.74800	1.55100	C	-6.99200	-0.52600	2.20400
C	-2.82100	3.80400	-2.43100	C	5.01600	-1.37500	-0.85200	C	-5.34600	-0.33400	-0.32100
C	-2.83100	2.69700	-3.31100	C	4.69700	-2.19600	0.25400	C	-5.23800	0.56100	0.76800
C	-2.91800	1.40600	-2.77700	C	3.36600	-2.60600	0.44200	C	-4.10200	1.37900	0.87000
C	-2.99500	1.22900	-1.39300	C	2.38500	-2.19600	-0.45600	C	-3.10300	1.29700	-0.09800
C	-2.99400	2.35800	-0.54300	C	2.73600	-1.37000	-1.54900	C	-3.23700	0.38800	-1.17200
C	-2.90200	3.65600	-1.05000	C	4.05200	-0.95400	-1.76400	C	-4.36100	-0.43400	-1.29900
N	-3.09700	1.93100	0.77800	N	1.60200	-1.09100	-2.30000	N	-2.11900	0.47300	-1.99700
C	-3.07300	0.06100	-0.53300	C	0.95100	-2.42500	-0.56300	C	-1.83500	1.98300	-0.29700
C	-3.12400	-1.31400	-0.77300	C	0.04000	-3.16800	0.18000	C	-1.18000	3.00200	0.39200
C	-3.21700	-2.20800	0.30100	C	-1.31100	-3.21200	-0.21100	C	0.04600	3.49100	-0.08600
C	-3.26300	-1.69200	1.61700	C	-1.71800	-2.47200	-1.34500	C	0.60000	2.91600	-1.25300
C	-3.21600	-0.32800	1.88300	C	-0.82300	-1.72500	-2.10700	C	-0.04000	1.90100	-1.96000
C	-3.12000	0.54100	0.79300	C	0.51700	-1.72500	-1.71200	C	-1.26900	1.45200	-1.47600
C	-2.68900	-5.61200	-1.29800	C	-2.97300	-5.18400	2.57900	C	1.17200	5.90800	2.62200
C	-3.41900	-6.44600	-0.44900	C	-4.03600	-5.79100	1.90500	C	1.89900	6.84400	1.88400
C	-4.06400	-5.89700	0.66100	C	-4.22900	-5.52000	0.54900	C	2.05100	6.66100	0.50800

C	-3.99500	-4.52500	0.90800	C	-3.35900	-4.66900	-0.13100	C	1.46700	5.56500	-0.12600
C	-3.26800	-3.67300	0.06000	C	-2.27400	-4.06500	0.52800	C	0.71900	4.62300	0.60000
C	-2.60300	-4.24500	-1.03900	C	-2.10600	-4.32800	1.90000	C	0.59400	4.80800	1.98800
H	-1.18900	4.88000	-7.09900	H	8.59600	-1.44800	2.70800	H	-8.50900	-1.37300	3.48000
H	-2.51700	3.44400	-8.64800	H	8.54300	-3.73000	3.71300	H	-9.14000	0.82100	4.48600
H	-3.99000	1.66100	-7.71300	H	6.70000	-5.29900	3.10700	H	-7.95500	2.89300	3.76100
H	-4.15000	1.33700	-5.26000	H	4.94700	-4.60000	1.50200	H	-6.17900	2.77400	2.03800
H	-1.31900	4.52200	-4.65000	H	6.81900	-0.73800	1.13400	H	-6.70500	-1.49400	1.78600
H	-2.77300	4.81000	-2.85500	H	6.05600	-1.08400	-1.00700	H	-6.24500	-0.94800	-0.40900
H	-2.89300	0.53800	-3.44100	H	3.10100	-3.22100	1.30600	H	-3.99200	2.05900	1.71900
H	-2.89900	4.52300	-0.38700	H	4.30800	-0.32700	-2.61900	H	-4.45900	-1.12300	-2.13900
H	-3.11800	-1.68900	-1.79900	H	0.38300	-3.75100	1.03800	H	-1.64200	3.44700	1.27600
H	-3.31100	-2.39100	2.45600	H	-2.77600	-2.46600	-1.61400	H	1.57800	3.26400	-1.59400
H	-3.24800	0.05100	2.90700	H	-1.15100	-1.14400	-2.97000	H	0.40900	1.45600	-2.85000
H	-2.15600	-6.03100	-2.15400	H	-2.81800	-5.37500	3.64400	H	1.05600	6.03100	3.70200
H	-3.47600	-7.51800	-0.64600	H	-4.71200	-6.46400	2.43500	H	2.34900	7.70700	2.37900
H	-4.64200	-6.53700	1.33100	H	-5.05900	-5.98300	0.01100	H	2.62200	7.38300	-0.08100
H	-4.54300	-4.10300	1.75300	H	-3.50100	-4.49900	-1.20000	H	1.56300	5.45600	-1.20900
H	-1.98700	-3.61300	-1.68200	H	-1.29400	-3.84300	2.44500	H	0.04700	4.07000	2.58000
C	-4.02400	3.55100	2.30100	C	2.17400	-1.18400	-4.66300	C	-2.98600	0.42900	-4.27200
C	-3.96100	4.30500	3.48200	C	2.23300	-0.60000	-5.92500	C	-2.98000	-0.08200	-5.56700
C	-2.85100	4.21700	4.30600	C	1.73700	0.70100	-6.11800	C	-2.06200	-1.08400	-5.92000
C	-1.77800	3.40200	3.92200	C	1.19600	1.39900	-5.04900	C	-1.16800	-1.56400	-4.97500
C	-1.78100	2.67900	2.72100	C	1.12400	0.83800	-3.75200	C	-1.15000	-1.06600	-3.65200
C	-2.95800	2.74000	1.93100	C	1.62700	-0.47400	-3.58800	C	-2.07900	-0.05500	-3.32100
C	-0.54700	1.96600	2.28500	C	0.58000	1.59200	-2.64600	C	-0.23000	-1.61500	-2.68500
C	0.38400	2.64300	4.50200	C	0.10600	3.37500	-4.31700	C	0.62600	-3.04900	-4.51500
C	1.38200	2.70000	5.48000	C	-0.38600	4.62700	-4.65300	C	1.46600	-4.04800	-4.98400
C	2.57300	2.02600	5.26000	C	-1.07200	5.39200	-3.69300	C	2.46000	-4.58000	-4.14500
C	2.76400	1.30400	4.07900	C	-1.29100	4.87300	-2.41900	C	2.62900	-4.07700	-2.85700
C	1.76900	1.24900	3.10200	C	-0.78900	3.61100	-2.08900	C	1.78300	-3.06500	-2.39300
C	0.53500	1.92100	3.30300	C	-0.04000	2.84600	-3.01500	C	0.73200	-2.56100	-3.19400
O	-0.73000	3.37100	4.78400	O	0.76200	2.67400	-5.29200	O	-0.32700	-2.57000	-5.37000
O	-0.42300	1.48800	1.16800	O	0.61600	1.17000	-1.44700	O	-0.24900	-1.26200	-1.45200
H	-4.91100	3.56200	1.66700	H	2.55000	-2.19400	-4.48800	H	-3.68900	1.21000	-3.97400
H	-2.77600	4.76200	5.24800	H	1.77500	1.19400	-7.09000	H	-2.03700	-1.51500	-6.92100
H	1.19100	3.28000	6.38200	H	-0.22100	4.99200	-5.66800	H	1.32400	-4.40600	-6.00500
H	3.36200	2.05300	6.01400	H	-1.45400	6.37800	-3.95700	H	3.11400	-5.37000	-4.51400
H	3.69600	0.76600	3.89600	H	-1.86600	5.42400	-1.67300	H	3.42600	-4.43800	-2.20500
H	-4.80100	4.94000	3.76800	H	2.66000	-1.14900	-6.76500	H	-3.68500	0.29500	-6.30800

Table S11: Coordinates of the optimized minimum in the S_0, S_1, and T_1 of 16PCX at the MN15/cc-pVDZ level.

S_0				S_1				T_1			
C	8.32400	6.31000	-1.80300	C	7.99700	6.29100	-2.78600	C	-8.04600	6.30500	2.40800
C	9.56700	6.25600	-1.16900	C	9.26200	6.35700	-2.19700	C	-9.30200	6.34400	1.80000

C	9.88000	5.15800	-0.36400	C	9.62500	5.40000	-1.24600	C	-9.66200	5.33400	0.90400
C	8.95800	4.12600	-0.19400	C	8.73200	4.39000	-0.88800	C	-8.77300	4.29800	0.61900
C	7.70500	4.16700	-0.82700	C	7.45800	4.31000	-1.47400	C	-7.50700	4.24500	1.22500
C	7.40300	5.27600	-1.63400	C	7.10700	5.27900	-2.43000	C	-7.15900	5.26600	2.12500
C	5.34400	3.34400	-0.54400	C	5.12500	3.50000	-0.98900	C	-5.17400	3.39100	0.82300
C	6.72500	3.06400	-0.64800	C	6.51000	3.23100	-1.09600	C	-6.56200	3.13800	0.92700
C	7.15100	1.73300	-0.57100	C	6.96900	1.93600	-0.83400	C	-7.02600	1.83100	0.74200
C	6.21200	0.71100	-0.40600	C	6.06000	0.93300	-0.48300	C	-6.11800	0.80200	0.47200
C	4.83600	1.01900	-0.32700	C	4.67900	1.22900	-0.39700	C	-4.73400	1.08300	0.39100
C	4.39000	2.34400	-0.38000	C	4.20000	2.52200	-0.64100	C	-4.25100	2.38700	0.55500
N	4.11300	-0.16500	-0.14700	N	3.98400	0.08000	-0.02600	N	-4.04200	-0.09300	0.10800
C	6.32900	-0.72900	-0.27200	C	6.20400	-0.47000	-0.14600	C	-6.26800	-0.61900	0.22600
C	7.41800	-1.60400	-0.29500	C	7.30200	-1.33300	-0.07100	C	-7.37200	-1.47600	0.19200
C	7.20700	-2.98200	-0.16900	C	7.10900	-2.67600	0.26600	C	-7.18600	-2.84200	-0.04900
C	5.88300	-3.45800	-0.03100	C	5.79600	-3.13600	0.52100	C	-5.87200	-3.32800	-0.24800
C	4.78300	-2.60700	-0.00200	C	4.68700	-2.29900	0.45600	C	-4.75800	-2.49600	-0.22200
C	5.01800	-1.23200	-0.11200	C	4.90300	-0.95500	0.12700	C	-4.96800	-1.13200	0.00900
C	10.64500	-4.48700	0.41000	C	10.58300	-4.05800	0.93900	C	-10.65900	-4.24100	-0.68800
C	10.52700	-5.72200	-0.23200	C	10.45600	-5.37900	0.50200	C	-10.55100	-5.52800	-0.15600
C	9.32300	-6.06200	-0.85200	C	9.23200	-5.81500	-0.01100	C	-9.34100	-5.93700	0.40900
C	8.24600	-5.17400	-0.83100	C	8.14900	-4.94000	-0.08700	C	-8.25100	-5.06700	0.44400
C	8.35100	-3.93000	-0.18900	C	8.26200	-3.61000	0.35000	C	-8.34500	-3.77000	-0.08900
C	9.56700	-3.60200	0.43200	C	9.49700	-3.18500	0.86600	C	-9.56700	-3.37300	-0.65600
H	8.07100	7.15900	-2.44200	H	7.70500	7.02900	-3.53700	H	-7.75700	7.08400	3.11700
H	10.28800	7.06500	-1.30200	H	9.96000	7.14900	-2.47600	H	-9.99700	7.15600	2.02200
H	10.84600	5.11000	0.14400	H	10.60800	5.44600	-0.77100	H	-10.63800	5.35800	0.41500
H	9.19900	3.28500	0.46100	H	9.01300	3.66300	-0.12200	H	-9.05000	3.52800	-0.10500
H	6.44400	5.31200	-2.15600	H	6.13000	5.21800	-2.91600	H	-6.18900	5.22800	2.62700
H	5.01700	4.38600	-0.56400	H	4.77500	4.52100	-1.15900	H	-4.82000	4.41900	0.93100
H	8.21400	1.49200	-0.66000	H	8.03300	1.70200	-0.92700	H	-8.09300	1.61000	0.83300
H	3.33300	2.59500	-0.27600	H	3.13900	2.75700	-0.54800	H	-3.18700	2.61100	0.46100
H	8.42900	-1.21700	-0.44300	H	8.30400	-0.96500	-0.30700	H	-8.37500	-1.08600	0.38500
H	5.72100	-4.53200	0.08000	H	5.65100	-4.18200	0.80300	H	-5.73200	-4.39200	-0.45300
H	3.77400	-3.00700	0.10000	H	3.68400	-2.67600	0.65900	H	-3.75500	-2.89600	-0.37800
H	11.57900	-4.21400	0.90500	H	11.53200	-3.70700	1.35100	H	-11.59700	-3.91300	-1.14100
H	11.37000	-6.41600	-0.24900	H	11.30400	-6.06300	0.56100	H	-11.40500	-6.20800	-0.18300
H	9.22200	-7.02100	-1.36500	H	9.12300	-6.84300	-0.36400	H	-9.24700	-6.93800	0.83600
H	7.31700	-5.43600	-1.34300	H	7.20400	-5.28300	-0.51700	H	-7.31800	-5.38500	0.91400
H	9.65700	-2.64900	0.96100	H	9.59600	-2.16300	1.23800	H	-9.65000	-2.37800	-1.10000
C	1.87500	0.38000	-0.96200	C	1.70000	0.36400	-0.85900	C	-1.77400	0.25500	0.95500
C	2.71000	-0.26400	-0.02300	C	2.57900	-0.01800	0.16200	C	-2.63700	-0.21300	-0.04400
C	2.15700	-1.00100	1.02300	C	2.07700	-0.49800	1.38100	C	-2.11300	-0.80500	-1.20300
C	0.76700	-1.10000	1.12300	C	0.70300	-0.60400	1.56100	C	-0.73800	-0.93300	-1.34800
C	-0.07700	-0.45900	0.21200	C	-0.20000	-0.21500	0.55200	C	0.15000	-0.45300	-0.36100
C	0.50000	0.28100	-0.83400	C	0.32600	0.27400	-0.65600	C	-0.39700	0.14500	0.78900
C	-1.54600	-0.55700	0.33100	C	-1.64000	-0.32500	0.77000	C	1.58100	-0.59700	-0.56000
C	-1.06500	-1.96200	2.33700	C	-1.05800	-1.21400	3.02800	C	1.04300	-1.67000	-2.73200
C	-1.43800	-2.79400	3.39700	C	-1.42700	-1.71100	4.26500	C	1.44300	-2.30200	-3.89700
C	-2.78600	-2.99300	3.65800	C	-2.79600	-1.86400	4.59000	C	2.81700	-2.47000	-4.17300

C	-3.75700	-2.34400	2.88900	C	-3.76900	-1.51300	3.66300	C	3.77200	-1.98700	-3.28500
C	-3.38500	-1.50700	1.83900	C	-3.38300	-1.01000	2.41400	C	3.35800	-1.34200	-2.11300
C	-2.01400	-1.31600	1.52000	C	-2.01800	-0.84200	2.05100	C	1.98800	-1.18800	-1.79700
O	0.28000	-1.84300	2.15100	O	0.27800	-1.08900	2.76900	O	-0.29600	-1.53900	-2.49600
O	-2.28700	-0.05500	-0.50200	O	-2.49300	0.01200	-0.10800	O	2.44400	-0.21100	0.30600
H	2.32700	0.92900	-1.78800	H	2.10700	0.72200	-1.80700	H	-2.19500	0.69500	1.86100
H	2.78600	-1.48600	1.76900	H	2.74700	-0.78400	2.19300	H	-2.76800	-1.16200	-1.99900
H	-0.18100	0.75700	-1.54100	H	-0.38800	0.56400	-1.42800	H	0.29400	0.50400	1.55200
H	-0.65100	-3.26700	3.98600	H	-0.63800	-1.97700	4.96900	H	0.67400	-2.67100	-4.57700
H	-3.08900	-3.64100	4.48200	H	-3.08300	-2.25600	5.56600	H	3.12700	-2.97300	-5.08900
H	-4.82000	-2.46200	3.10700	H	-4.83100	-1.62200	3.88900	H	4.84000	-2.08500	-3.49000
C	-6.27200	6.80500	1.45400	C	-8.44100	6.08000	1.39600	C	7.13400	6.55800	-1.68700
C	-6.87600	7.21400	0.26200	C	-8.85500	6.38000	0.09600	C	7.64700	6.96000	-0.45200
C	-7.03900	6.29300	-0.77500	C	-8.50800	5.52600	-0.95400	C	7.58300	6.08700	0.63700
C	-6.60200	4.97700	-0.62200	C	-7.75600	4.37900	-0.70600	C	7.01100	4.82400	0.49200
C	-5.99600	4.55100	0.57200	C	-7.33000	4.06700	0.59800	C	6.49200	4.40700	-0.74500
C	-5.83900	5.48800	1.60700	C	-7.68400	4.93600	1.64400	C	6.56200	5.29500	-1.83200
C	-4.34200	2.86200	1.43400	C	-5.52400	2.85600	1.85300	C	4.76000	2.87700	-1.73100
C	-5.53900	3.14800	0.73600	C	-6.52900	2.85000	0.86100	C	5.88600	3.06200	-0.90000
C	-6.28200	2.08800	0.20600	C	-6.76600	1.66900	0.11800	C	6.43200	1.95400	-0.22300
C	-5.83600	0.77400	0.37400	C	-6.01200	0.53800	0.38000	C	5.85400	0.70000	-0.38500
C	-4.63200	0.52300	1.06700	C	-5.01000	0.58100	1.37900	C	4.72400	0.55500	-1.21700
C	-3.87500	1.56500	1.60800	C	-4.75200	1.73100	2.12800	C	4.16300	1.63000	-1.90300
N	-4.41400	-0.85400	1.12100	N	-4.38800	-0.64900	1.46000	N	4.31900	-0.78200	-1.21800
C	-6.37600	-0.51900	-0.00600	C	-5.99100	-0.82300	-0.15800	C	6.16900	-0.63400	0.11800
C	-7.51800	-0.91500	-0.70700	C	-6.70900	-1.48900	-1.13600	C	7.15300	-1.14200	0.95600
C	-7.76300	-2.27600	-0.92900	C	-6.41000	-2.84300	-1.41900	C	7.17700	-2.52200	1.24400
C	-6.83800	-3.22600	-0.43900	C	-5.38800	-3.49400	-0.69400	C	6.20000	-3.36700	0.67100
C	-5.69300	-2.85700	0.26100	C	-4.65500	-2.84400	0.29500	C	5.20400	-2.88000	-0.17000
C	-5.47400	-1.49300	0.47700	C	-4.97100	-1.50700	0.54700	C	5.20200	-1.50800	-0.43300
C	-11.33700	-2.46700	-2.19800	C	-9.23200	-3.99500	-3.67400	C	10.50800	-3.09600	2.96600
C	-11.27000	-3.54800	-3.08000	C	-8.58500	-4.94300	-4.47100	C	10.19700	-4.13700	3.84300
C	-10.05500	-4.21400	-3.25800	C	-7.22700	-5.20400	-4.26900	C	8.89900	-4.65200	3.86900
C	-8.91900	-3.80200	-2.56100	C	-6.52200	-4.52400	-3.27800	C	7.92000	-4.13000	3.02500
C	-8.97300	-2.71700	-1.67000	C	-7.16000	-3.56700	-2.47000	C	8.21900	-3.08100	2.13900
C	-10.20200	-2.05800	-1.50000	C	-8.52600	-3.31100	-2.68600	C	9.52900	-2.57200	2.12400
H	-6.14600	7.51400	2.27500	H	-8.71500	6.73700	2.22300	H	7.18700	7.22900	-2.54700
H	-7.21600	8.24400	0.14200	H	-9.44500	7.27700	-0.09900	H	8.09400	7.94900	-0.33800
H	-7.50100	6.60300	-1.71500	H	-8.81700	5.75900	-1.97500	H	7.97200	6.39600	1.61000
H	-6.70600	4.26900	-1.44800	H	-7.46400	3.73300	-1.53700	H	6.93800	4.15800	1.35500
H	-5.39400	5.17000	2.55300	H	-7.38800	4.69500	2.66700	H	6.18800	4.97800	-2.80900
H	-3.75200	3.69400	1.82500	H	-5.32800	3.78200	2.39700	H	4.32700	3.74600	-2.23000
H	-7.22600	2.28400	-0.30800	H	-7.55900	1.64900	-0.63300	H	7.32200	2.07900	0.39800
H	-2.94200	1.36900	2.13900	H	-3.96200	1.73800	2.87900	H	3.28200	1.49300	-2.53200
H	-8.20500	-0.16400	-1.10500	H	-7.48000	-0.97100	-1.71100	H	7.89000	-0.47700	1.41300
H	-7.04200	-4.28700	-0.59700	H	-5.18500	-4.54600	-0.90100	H	6.24500	-4.43800	0.87700
H	-4.99400	-3.60900	0.63000	H	-3.86700	-3.34300	0.85800	H	4.45300	-3.53400	-0.61400
H	-12.28400	-1.94400	-2.04500	H	-10.29500	-3.79200	-3.81800	H	11.52300	-2.69300	2.93100
H	-12.15900	-3.87000	-3.62600	H	-9.13800	-5.47700	-5.24600	H	10.96400	-4.54700	4.50400

H	-9.98800	-5.05500	-3.95100	H	-6.71200	-5.93500	-4.89500	H	8.64400	-5.45900	4.55800
H	-7.96600	-4.31000	-2.72800	H	-5.45400	-4.71300	-3.14700	H	6.90000	-4.51800	3.07200
H	-10.26900	-1.23000	-0.79000	H	-9.04600	-2.59100	-2.05000	H	9.78700	-1.77600	1.42100

Table S12: Coordinates of the optimized minimum in the S_0, S_1, and T_1 of 27PCX at the MN15/cc-pVDZ level.

S_0				S_1				T_1			
C	7.29800	6.79000	-1.05400	C	7.06400	6.82300	-0.99400	C	6.97600	6.80000	-1.10500
C	8.64800	6.95000	-0.73800	C	8.45000	6.96900	-0.87900	C	8.31200	7.01900	-0.76000
C	9.35700	5.87800	-0.19100	C	9.23000	5.88000	-0.48100	C	9.05500	5.98100	-0.19300
C	8.72100	4.65900	0.03900	C	8.63100	4.65400	-0.20400	C	8.46800	4.73600	0.02600
C	7.36300	4.48300	-0.27700	C	7.23600	4.49400	-0.30900	C	7.12600	4.50300	-0.31600
C	6.66300	5.56900	-0.82700	C	6.46200	5.60100	-0.70800	C	6.38900	5.55500	-0.88400
C	5.35000	3.14600	0.42300	C	5.29500	3.14600	0.53700	C	5.15600	3.08200	0.33700
C	6.68700	3.18200	-0.03700	C	6.59900	3.19800	-0.01100	C	6.50100	3.17600	-0.08400
C	7.35800	1.97500	-0.25800	C	7.29100	1.98500	-0.26900	C	7.23600	1.99600	-0.26700
C	6.70000	0.76200	-0.03100	C	6.67400	0.77900	-0.00100	C	6.62300	0.76300	-0.05200
C	5.35900	0.75800	0.41600	C	5.35700	0.75900	0.53700	C	5.26900	0.69600	0.34300
C	4.67300	1.95400	0.65600	C	4.66000	1.94600	0.82700	C	4.52700	1.86200	0.56300
N	4.93500	-0.56300	0.57900	N	4.96000	-0.54200	0.72500	N	4.91200	-0.65000	0.52700
C	7.10400	-0.62600	-0.15200	C	7.07400	-0.62000	-0.14300	C	7.10900	-0.60600	-0.13000
C	8.29000	-1.24900	-0.55200	C	8.21300	-1.25000	-0.61200	C	8.33700	-1.15600	-0.49400
C	8.37200	-2.64500	-0.57100	C	8.27300	-2.66500	-0.62000	C	8.49900	-2.54900	-0.50600
C	7.23700	-3.40100	-0.19600	C	7.15700	-3.40400	-0.17000	C	7.39400	-3.36500	-0.17500
C	6.04500	-2.80600	0.20300	C	5.99900	-2.79000	0.29800	C	6.15800	-2.83600	0.18400
C	5.99200	-1.40800	0.23500	C	5.97700	-1.39000	0.32100	C	6.03200	-1.44300	0.23400
C	11.59800	-3.46400	-2.40200	C	11.41500	-3.47700	-2.58100	C	11.83000	-3.17100	-2.21700
C	12.00700	-4.62900	-1.74900	C	11.80700	-4.68200	-1.99300	C	12.27300	-4.32200	-1.56100
C	11.22700	-5.14500	-0.71200	C	11.04300	-5.22700	-0.95700	C	11.48100	-4.89400	-0.56300
C	10.05000	-4.50100	-0.33200	C	9.89500	-4.57300	-0.51500	C	10.25700	-4.31800	-0.22300
C	9.62500	-3.33000	-0.98100	C	9.49000	-3.35800	-1.09600	C	9.80000	-3.16000	-0.87400
C	10.41900	-2.82300	-2.02300	C	10.27000	-2.81900	-2.13500	C	10.60600	-2.59600	-1.87700
H	6.73500	7.61800	-1.49000	H	6.45000	7.66500	-1.31600	H	6.38800	7.60200	-1.55600
H	9.14600	7.90600	-0.91600	H	8.92000	7.92800	-1.10000	H	8.77100	7.99400	-0.93200
H	10.41200	5.99500	0.06900	H	10.31100	5.98900	-0.37800	H	10.09800	6.14500	0.08900
H	9.27400	3.83400	0.49400	H	9.24700	3.81800	0.13500	H	9.04800	3.93800	0.49700
H	5.61300	5.44300	-1.10300	H	5.38300	5.48900	-0.82800	H	5.35200	5.38300	-1.18100
H	4.84000	4.09000	0.62700	H	4.78500	4.08000	0.77300	H	4.59700	4.00200	0.52200
H	8.38600	1.97900	-0.62800	H	8.29100	2.01100	-0.70500	H	8.27700	2.04200	-0.59500
H	3.64700	1.95800	1.02700	H	3.66500	1.91400	1.26700	H	3.49700	1.82800	0.91600
H	9.15900	-0.64600	-0.82700	H	9.07200	-0.66700	-0.95000	H	9.17600	-0.50400	-0.75100
H	7.29100	-4.49100	-0.25200	H	7.19100	-4.49300	-0.22300	H	7.50400	-4.44900	-0.23900
H	5.17700	-3.41500	0.46200	H	5.13400	-3.36600	0.62300	H	5.31300	-3.49200	0.39200
H	12.19500	-3.05600	-3.22100	H	12.00000	-3.05100	-3.39800	H	12.43800	-2.72200	-3.00600
H	12.92900	-5.13200	-2.04700	H	12.70400	-5.19600	-2.34100	H	13.23100	-4.77300	-1.82800
H	11.54200	-6.05000	-0.18900	H	11.34700	-6.16300	-0.48500	H	11.82200	-5.78800	-0.03800

H	9.45900	-4.89600	0.49900	H	9.32000	-4.99200	0.31400	H	9.65600	-4.75500	0.57800
H	10.09000	-1.92800	-2.55800	H	9.95700	-1.89300	-2.62100	H	10.25400	-1.71200	-2.41400
C	-7.84100	6.75400	0.53100	C	-7.83000	6.74200	0.41500	C	-7.62200	6.74100	0.76900
C	-8.72500	6.92500	-0.53700	C	-8.68900	6.89700	-0.67600	C	-8.50900	6.96300	-0.28700
C	-8.94600	5.86600	-1.42100	C	-8.88400	5.82600	-1.55100	C	-8.76600	5.93300	-1.19500
C	-8.28900	4.64900	-1.23800	C	-8.22400	4.61500	-1.33900	C	-8.14100	4.69500	-1.04900
C	-7.40000	4.46300	-0.16700	C	-7.36000	4.44400	-0.24600	C	-7.24800	4.45800	0.00900
C	-7.18700	5.53500	0.71400	C	-7.17500	5.52900	0.62700	C	-6.99900	5.50200	0.91500
C	-5.36700	3.12900	0.48400	C	-5.34000	3.13000	0.48300	C	-5.25100	3.04900	0.61200
C	-6.70600	3.16300	0.03000	C	-6.66500	3.15100	-0.01500	C	-6.59000	3.13600	0.16600
C	-7.36600	1.95500	-0.22000	C	-7.30800	1.93700	-0.27700	C	-7.28500	1.95400	-0.11700
C	-6.69600	0.74400	-0.02400	C	-6.63800	0.73200	-0.04600	C	-6.64700	0.72100	0.03500
C	-5.35400	0.74200	0.42000	C	-5.31100	0.74300	0.44600	C	-5.30200	0.66400	0.46300
C	-4.67800	1.93800	0.68500	C	-4.65100	1.94700	0.71800	C	-4.59400	1.83300	0.76900
N	-4.92100	-0.57900	0.55900	N	-4.87100	-0.56700	0.61200	N	-4.90900	-0.67500	0.56200
C	-7.09300	-0.64500	-0.16000	C	-7.01700	-0.66100	-0.18200	C	-7.09100	-0.65000	-0.13700
C	-8.27800	-1.27000	-0.56100	C	-8.18000	-1.30500	-0.61300	C	-8.29800	-1.21700	-0.55200
C	-8.35000	-2.66700	-0.60000	C	-8.23600	-2.70300	-0.63300	C	-8.41800	-2.60900	-0.64400
C	-7.20800	-3.42000	-0.24300	C	-7.09800	-3.43900	-0.22500	C	-7.29600	-3.41100	-0.33100
C	-6.01700	-2.82300	0.15800	C	-5.92900	-2.82300	0.20600	C	-6.08400	-2.86900	0.08200
C	-5.97400	-1.42500	0.20900	C	-5.89900	-1.42400	0.23600	C	-5.99500	-1.47700	0.19800
C	-11.58300	-3.48100	-2.42200	C	-11.40600	-3.57800	-2.53700	C	-11.70000	-3.23100	-2.45100
C	-11.98000	-4.65800	-1.78300	C	-11.80700	-4.75400	-1.89900	C	-12.12400	-4.42700	-1.86700
C	-11.18900	-5.18400	-0.75900	C	-11.03700	-5.25900	-0.84800	C	-11.33400	-5.02900	-0.88500
C	-10.01400	-4.53700	-0.37700	C	-9.88200	-4.59300	-0.43900	C	-10.13300	-4.43900	-0.49000
C	-9.60200	-3.35400	-1.01100	C	-9.46600	-3.41000	-1.07300	C	-9.69500	-3.23700	-1.06900
C	-10.40500	-2.83700	-2.04000	C	-10.24800	-2.91600	-2.13000	C	-10.49800	-2.64300	-2.05700
H	-7.66600	7.57200	1.23300	H	-7.67600	7.56900	1.11200	H	-7.42000	7.53600	1.49000
H	-9.23700	7.87800	-0.68000	H	-9.20400	7.84500	-0.84200	H	-8.99700	7.93200	-0.40200
H	-9.62800	5.99100	-2.26400	H	-9.54600	5.93700	-2.41200	H	-9.45000	6.09800	-2.03000
H	-8.44500	3.83500	-1.95000	H	-8.35900	3.79200	-2.04600	H	-8.32400	3.90400	-1.78000
H	-6.51900	5.40100	1.56800	H	-6.52700	5.40800	1.49900	H	-6.32800	5.32800	1.76000
H	-4.84800	4.07400	0.65900	H	-4.83300	4.08100	0.66300	H	-4.70500	3.97200	0.81700
H	-8.41100	1.95800	-0.53900	H	-8.34200	1.93000	-0.63100	H	-8.33400	1.99500	-0.42300
H	-3.64400	1.94400	1.03200	H	-3.62700	1.95400	1.09300	H	-3.56200	1.80000	1.11600
H	-9.15300	-0.66900	-0.82100	H	-9.05400	-0.71800	-0.90900	H	-9.15300	-0.57600	-0.78300
H	-7.25500	-4.50900	-0.31300	H	-7.13200	-4.52900	-0.27900	H	-7.37700	-4.49400	-0.44900
H	-5.14300	-3.42900	0.40300	H	-5.05700	-3.41000	0.50000	H	-5.22700	-3.51200	0.28600
H	-12.18800	-3.06500	-3.23000	H	-11.99400	-3.17800	-3.36600	H	-12.30400	-2.75700	-3.22700
H	-12.90000	-5.16300	-2.08300	H	-12.71200	-5.27400	-2.21900	H	-13.06400	-4.88800	-2.17600
H	-11.49400	-6.09900	-0.24700	H	-11.34400	-6.17300	-0.33500	H	-11.65900	-5.95900	-0.41500
H	-9.41500	-4.94000	0.44400	H	-9.30200	-4.97900	0.40200	H	-9.53500	-4.90100	0.30000
H	-10.08600	-1.93200	-2.56400	H	-9.92600	-2.01200	-2.65300	H	-10.15900	-1.72400	-2.54000
C	-3.65300	-0.99100	1.03700	C	-3.60700	-0.96100	1.13900	C	-3.64700	-1.13800	0.99100
C	-3.57300	-1.89000	2.12200	C	-3.55700	-1.80500	2.25500	C	-3.57300	-2.16700	1.94800
C	-2.34500	-2.30900	2.60600	C	-2.31500	-2.19700	2.77100	C	-2.32200	-2.62300	2.37100
C	-1.17000	-1.81400	2.02500	C	-1.14600	-1.72700	2.18500	C	-1.16900	-2.04300	1.84900
C	-1.23200	-0.90900	0.95800	C	-1.17700	-0.86800	1.07000	C	-1.21800	-1.00100	0.89400
C	-2.48400	-0.51300	0.46100	C	-2.42800	-0.50200	0.54900	C	-2.48000	-0.56500	0.46700

C	0.00800	-0.38500	0.34700	C	0.06300	-0.37600	0.45700	C	0.01100	-0.41900	0.36600
C	1.18300	-1.80800	2.03100	C	1.22400	-1.72900	2.22200	C	1.20400	-2.04500	1.84600
C	2.35600	-2.29700	2.62000	C	2.37700	-2.19200	2.84000	C	2.36700	-2.61900	2.35400
C	3.58600	-1.87200	2.14100	C	3.64900	-1.80800	2.35100	C	3.61400	-2.16700	1.91500
C	3.66800	-0.97700	1.05400	C	3.68500	-0.95200	1.24500	C	3.66400	-1.12000	0.95400
C	2.49800	-0.50500	0.47100	C	2.53800	-0.47800	0.61500	C	2.49400	-0.54300	0.44700
C	1.24600	-0.90500	0.96300	C	1.26100	-0.86200	1.09800	C	1.23100	-0.98600	0.88100
O	0.00600	-2.25100	2.55200	O	0.03700	-2.14200	2.74700	O	0.02600	-2.53100	2.30100
O	0.00800	0.40900	-0.58300	O	0.07400	0.39600	-0.52900	O	0.00000	0.52300	-0.47700
H	-2.26500	-3.00400	3.44300	H	-2.24000	-2.85600	3.63700	H	-2.22200	-3.41100	3.11700
H	-2.50100	0.17500	-0.38700	H	-2.43500	0.14600	-0.33000	H	-2.51300	0.21100	-0.29900
H	2.27600	-2.99000	3.45800	H	2.27500	-2.84000	3.71000	H	2.27400	-3.40100	3.10800
H	4.50800	-2.22300	2.60700	H	4.56600	-2.12000	2.84900	H	4.53500	-2.54700	2.35400
H	2.51600	0.18100	-0.37900	H	2.56800	0.15500	-0.27400	H	2.51800	0.22000	-0.33000
H	-4.49700	-2.24600	2.58100	H	-4.48500	-2.14200	2.72000	H	-4.48800	-2.57400	2.37700

Table S13: Coordinates of the optimized minimum in the S_0, S_1, and T_1 of 26PCX at the MN15/cc-pVDZ level.

S_0				S_1				T_1			
C	10.76200	-5.68300	0.34000	C	-10.49100	4.59400	3.77000	C	10.71800	-5.70800	-0.31800
C	11.75300	-5.32900	-0.57800	C	-11.50700	4.88300	2.85600	C	11.72300	-5.26600	-1.18200
C	11.61400	-4.15400	-1.32000	C	-11.42600	4.37200	1.55800	C	11.60200	-4.01400	-1.78900
C	10.49400	-3.34100	-1.14400	C	-10.34000	3.58300	1.18000	C	10.48700	-3.21400	-1.53700
C	9.49200	-3.68400	-0.22200	C	-9.31300	3.28200	2.08900	C	9.47100	-3.64500	-0.66900
C	9.64400	-4.86800	0.51700	C	-9.40800	3.80100	3.39100	C	9.60600	-4.90600	-0.06400
C	7.02500	-3.38200	0.18000	C	-6.86000	2.72300	2.16800	C	7.00500	-3.37100	-0.24800
C	8.30200	-2.81300	-0.03500	C	-8.16000	2.43500	1.68700	C	8.28700	-2.79100	-0.39400
C	8.42100	-1.42000	-0.07900	C	-8.33800	1.34400	0.83000	C	8.41800	-1.40300	-0.27800
C	7.28900	-0.62000	0.09900	C	-7.24000	0.55600	0.47100	C	7.29200	-0.61800	-0.01000
C	6.02900	-1.21500	0.33200	C	-5.95400	0.86000	0.97800	C	6.02600	-1.22900	0.14900
C	5.88400	-2.60700	0.35900	C	-5.75200	1.95600	1.82600	C	5.87000	-2.61300	0.01900
N	5.06400	-0.20900	0.45600	N	-5.03300	-0.05600	0.47900	N	5.07000	-0.24500	0.39900
C	7.07800	0.81600	0.08400	C	-7.08200	-0.60700	-0.38100	C	7.08800	0.80900	0.15500
C	7.93900	1.90500	-0.07600	C	-7.97300	-1.37400	-1.13800	C	7.94900	1.91000	0.12900
C	7.43400	3.20800	-0.01000	C	-7.50600	-2.48200	-1.85300	C	7.44300	3.19400	0.35900
C	6.05300	3.39100	0.23100	C	-6.13000	-2.80600	-1.78800	C	6.06200	3.34800	0.62200
C	5.17500	2.32300	0.39200	C	-5.22200	-2.06000	-1.04500	C	5.18500	2.26900	0.65100
C	5.69900	1.02900	0.30400	C	-5.70900	-0.95000	-0.34400	C	5.70900	0.99500	0.40600
C	10.22800	5.44800	-1.27200	C	-10.37200	-3.49700	-4.13100	C	10.24200	5.57500	-0.60400
C	10.03900	6.60000	-0.50500	C	-10.21200	-4.88300	-4.19200	C	10.05500	6.61800	0.30500
C	8.99700	6.64300	0.42300	C	-9.16500	-5.48300	-3.48900	C	9.01200	6.54300	1.23100
C	8.15200	5.54400	0.58400	C	-8.28900	-4.70500	-2.73200	C	8.16500	5.43400	1.24700
C	8.33000	4.38000	-0.18100	C	-8.43600	-3.31000	-2.66200	C	8.34100	4.37800	0.33800
C	9.38100	4.35100	-1.11200	C	-9.49200	-2.72000	-3.37700	C	9.39300	4.46800	-0.58800
H	10.86400	-6.59600	0.93100	H	-10.54700	4.98100	4.79000	H	10.80500	-6.68200	0.16900
H	12.62800	-5.96700	-0.71600	H	-12.35600	5.50300	3.15200	H	12.59400	-5.89300	-1.38000

H	12.37800	-3.87200	-2.04700	H	-12.20900	4.59800	0.83100	H	12.37700	-3.66100	-2.47300
H	10.37800	-2.43800	-1.74900	H	-10.27000	3.21100	0.15400	H	10.38600	-2.24900	-2.03800
H	8.88700	-5.13800	1.25700	H	-8.63200	3.55700	4.12100	H	8.83800	-5.24800	0.63500
H	6.92800	-4.47000	0.17300	H	-6.72000	3.59700	2.80800	H	6.90100	-4.45100	-0.37600
H	9.39900	-0.95800	-0.23300	H	-9.33600	1.09400	0.46200	H	9.40100	-0.93500	-0.37200
H	4.90900	-3.07600	0.49500	H	-4.75600	2.20400	2.19500	H	4.89000	-3.08300	0.10800
H	9.01000	1.74000	-0.22100	H	-9.03800	-1.12700	-1.14600	H	9.01900	1.76500	-0.03900
H	5.65700	4.40900	0.27000	H	-5.76500	-3.66100	-2.36300	H	5.66800	4.35400	0.78500
H	4.11600	2.49900	0.58200	H	-4.16500	-2.32600	-1.01100	H	4.12300	2.41500	0.85700
H	11.03500	5.40600	-2.00700	H	-11.18100	-3.01400	-4.68300	H	11.04900	5.62600	-1.33800
H	10.70000	7.45900	-0.63100	H	-10.89900	-5.49100	-4.78400	H	10.71800	7.48500	0.29200
H	8.84500	7.53500	1.03500	H	-9.03500	-6.56700	-3.52100	H	8.86100	7.34900	1.95200
H	7.35700	5.57600	1.33300	H	-7.49100	-5.18700	-2.16200	H	7.37000	5.37000	1.99400
H	9.51800	3.46400	-1.73600	H	-9.60600	-1.63300	-3.35800	H	9.52800	3.66900	-1.32100
C	3.26500	-1.27000	1.72300	C	-3.19000	-0.13800	2.08900	C	3.26400	-1.37400	1.60000
C	3.68600	-0.41300	0.68300	C	-3.63900	-0.07400	0.76600	C	3.68700	-0.47000	0.61700
C	2.75000	0.23500	-0.12000	C	-2.71600	-0.02000	-0.29200	C	2.74200	0.21600	-0.16000
C	1.38700	0.03500	0.12000	C	-1.35600	-0.04300	-0.00800	C	1.39000	-0.01000	0.07100
C	0.95100	-0.82200	1.14000	C	-0.87900	-0.09400	1.31500	C	0.94000	-0.92000	1.04100
C	1.91200	-1.47000	1.93400	C	-1.82200	-0.13700	2.35400	C	1.90200	-1.59900	1.79800
C	-0.48800	-1.03900	1.37600	C	0.56200	-0.10300	1.60000	C	-0.50100	-1.14200	1.24400
C	-0.81700	0.53700	-0.53600	C	0.84300	0.00300	-0.89100	C	-0.82400	0.55500	-0.58400
C	-1.64700	1.24200	-1.41700	C	1.64000	0.05100	-2.02500	C	-1.64200	1.32000	-1.40300
C	-3.02300	1.10600	-1.31100	C	3.05000	0.06100	-1.90400	C	-3.03400	1.22400	-1.29300
C	-3.59300	0.28900	-0.31300	C	3.58800	0.00900	-0.61300	C	-3.58400	0.30300	-0.32900
C	-2.76100	-0.39700	0.56400	C	2.80300	-0.04200	0.53500	C	-2.76500	-0.47400	0.48500
C	-1.36700	-0.29000	0.45100	C	1.38900	-0.04800	0.41900	C	-1.36100	-0.37800	0.39600
O	0.52600	0.69900	-0.69700	O	-0.50600	0.00100	-1.08700	O	0.51100	0.70100	-0.71700
O	-0.92000	-1.77400	2.25300	O	1.02600	-0.15400	2.76200	O	-0.93300	-1.96200	2.10000
H	4.01300	-1.74600	2.35700	H	-3.92200	-0.19500	2.89800	H	4.01200	-1.88600	2.20800
H	3.05900	0.88000	-0.94300	H	-3.04900	0.04700	-1.32900	H	3.05000	0.91100	-0.94200
H	1.54300	-2.12100	2.72900	H	-1.42700	-0.18300	3.37000	H	1.53600	-2.29800	2.55200
H	-1.19000	1.87000	-2.18200	H	1.15400	0.06900	-3.00100	H	-1.17200	1.97300	-2.13900
H	-3.68200	1.62700	-2.00900	H	3.69600	0.04900	-2.78200	H	-3.69300	1.72700	-1.99800
H	-3.16100	-1.03200	1.35700	H	3.22300	-0.03800	1.54200	H	-3.17200	-1.12300	1.26100
C	-9.26300	-6.42500	-1.08500	C	10.07800	5.01200	-3.80400	C	-9.25600	-6.46200	-0.29700
C	-10.41800	-6.36400	-0.30300	C	11.19400	5.19400	-2.98300	C	-10.42700	-6.28400	0.44400
C	-10.64800	-5.24200	0.49600	C	11.26100	4.53700	-1.75100	C	-10.66500	-5.06100	1.07500
C	-9.73100	-4.19200	0.51400	C	10.22200	3.70200	-1.34500	C	-9.73900	-4.02400	0.96500
C	-8.56500	-4.23900	-0.26900	C	9.09100	3.51300	-2.16000	C	-8.55800	-4.18900	0.22300
C	-8.34700	-5.37300	-1.06900	C	9.03500	4.18300	-3.39600	C	-8.33000	-5.42500	-0.40600
C	-6.20300	-3.37200	-0.32100	C	6.65000	2.93300	-2.07600	C	-6.19100	-3.34900	0.08100
C	-7.59200	-3.11600	-0.25600	C	7.98500	2.63100	-1.73000	C	-7.57600	-3.08300	0.10700
C	-8.03200	-1.79000	-0.18600	C	8.25400	1.47200	-0.96200	C	-8.01000	-1.75100	0.00600
C	-7.10000	-0.74800	-0.17700	C	7.20800	0.64900	-0.58200	C	-7.07100	-0.72800	-0.09700
C	-5.71700	-1.03600	-0.23000	C	5.88000	0.97100	-0.96000	C	-5.69000	-1.01900	-0.08900
C	-5.25600	-2.35400	-0.31000	C	5.58300	2.12400	-1.69800	C	-5.23600	-2.34100	-0.02300
N	-5.00000	0.16400	-0.22600	N	5.01600	0.01400	-0.45400	N	-4.96900	0.17900	-0.25100
C	-7.22500	0.69700	-0.14600	C	7.12000	-0.58300	0.19900	C	-7.20200	0.71200	-0.26100

C	-8.32100	1.56300	-0.08200	C	8.04700	-1.38700	0.83200	C	-8.30400	1.56200	-0.29500
C	-8.11400	2.94600	-0.03600	C	7.61000	-2.54200	1.53200	C	-8.10900	2.94900	-0.39300
C	-6.78900	3.43900	-0.04500	C	6.23100	-2.86000	1.54800	C	-6.79000	3.44900	-0.42800
C	-5.68200	2.59900	-0.11000	C	5.28200	-2.07500	0.90800	C	-5.67400	2.61600	-0.38900
C	-5.91200	1.22000	-0.17300	C	5.73800	-0.92200	0.24500	C	-5.89400	1.23500	-0.34200
C	-11.52600	4.50000	-0.64100	C	10.81500	-4.36200	2.40200	C	-11.53600	4.38700	-1.15200
C	-11.45600	5.65600	0.13900	C	10.44600	-5.03100	3.57200	C	-11.47700	5.62100	-0.50000
C	-10.29200	5.92600	0.86300	C	9.14900	-4.88600	4.07400	C	-10.31500	5.98200	0.18700
C	-9.21000	5.04900	0.80700	C	8.22700	-4.08300	3.40900	C	-9.22200	5.11700	0.22100
C	-9.26500	3.88300	0.02500	C	8.58600	-3.39900	2.23200	C	-9.26900	3.87400	-0.43100
C	-10.44100	3.62500	-0.69900	C	9.89700	-3.54900	1.74200	C	-10.44300	3.52200	-1.11700
H	-9.07600	-7.29400	-1.71900	H	10.02200	5.51400	-4.77100	H	-9.06600	-7.41100	-0.80100
H	-11.13500	-7.18700	-0.31500	H	12.00800	5.84600	-3.30100	H	-11.15100	-7.09700	0.52900
H	-11.54400	-5.18800	1.11900	H	12.12400	4.68300	-1.09900	H	-11.57200	-4.91600	1.66400
H	-9.90300	-3.33000	1.16300	H	10.27100	3.21600	-0.36800	H	-9.91800	-3.08000	1.48500
H	-7.46000	-5.41600	-1.70500	H	8.18000	4.02500	-4.05600	H	-7.43000	-5.56300	-1.00900
H	-5.86200	-4.40900	-0.35500	H	6.44400	3.85100	-2.62800	H	-5.85100	-4.38300	0.17000
H	-9.10200	-1.56800	-0.17000	H	9.28300	1.22200	-0.69500	H	-9.07700	-1.52300	-0.02900
H	-4.19000	-2.58300	-0.35700	H	4.55300	2.37500	-1.94700	H	-4.17600	-2.58700	-0.05100
H	-9.33600	1.16000	-0.03800	H	9.10700	-1.12300	0.82500	H	-9.31400	1.15600	-0.20400
H	-6.63100	4.52000	-0.02200	H	5.90600	-3.77100	2.05200	H	-6.63700	4.52800	-0.50000
H	-4.67000	3.00800	-0.11000	H	4.22500	-2.33500	0.90900	H	-4.66800	3.03400	-0.38400
H	-12.42700	4.28200	-1.21800	H	11.82200	-4.47900	1.99900	H	-12.43600	4.10000	-1.69900
H	-12.30400	6.34200	0.18400	H	11.16700	-5.66400	4.09200	H	-12.33200	6.29900	-0.52700
H	-10.23000	6.82300	1.48400	H	8.85700	-5.39700	4.99300	H	-10.26300	6.94000	0.70700
H	-8.31400	5.25400	1.39800	H	7.22500	-3.95400	3.82400	H	-8.32800	5.39600	0.78400
H	-10.49200	2.73800	-1.33500	H	10.18800	-3.05400	0.81300	H	-10.48700	2.57000	-1.65200

Table S14: Coordinates of the optimized minimum in the S_0, S_1, and T_1 of 36PCX at the MN15/cc-pVDZ level.

S_0				S_1				T_1			
C	11.29300	-4.60500	-1.39500	C	-11.19000	4.54300	0.23100	C	-7.22700	6.78600	0.91600
C	11.91500	-3.98200	-2.48000	C	-11.77200	4.37800	-1.02800	C	-7.96100	7.09300	-0.23100
C	11.33900	-2.84000	-3.03900	C	-11.17500	3.51400	-1.95000	C	-8.13400	6.11900	-1.21600
C	10.15100	-2.32600	-2.51900	C	-10.00900	2.82600	-1.61600	C	-7.57800	4.85000	-1.05500
C	9.51600	-2.94000	-1.42700	C	-9.41500	2.97900	-0.35300	C	-6.83900	4.52600	0.09500
C	10.10700	-4.08800	-0.87400	C	-10.02600	3.85000	0.56500	C	-6.67400	5.51500	1.07800
C	7.23200	-3.26000	-0.42100	C	-7.17600	2.85400	0.79200	C	-4.98600	3.01000	0.87200
C	8.25200	-2.39100	-0.87100	C	-8.17700	2.23800	0.00300	C	-6.25400	3.17100	0.26700
C	8.04600	-1.01000	-0.79000	C	-7.97900	0.92500	-0.43600	C	-6.94900	2.03300	-0.15800
C	6.85200	-0.51600	-0.25600	C	-6.80800	0.24400	-0.08700	C	-6.38200	0.76700	0.01200
C	5.86000	-1.40800	0.20700	C	-5.83100	0.88200	0.71300	C	-5.10400	0.63800	0.60500
C	6.03600	-2.79300	0.11300	C	-6.00400	2.20000	1.15300	C	-4.39900	1.76100	1.05000
N	4.76500	-0.67300	0.67300	N	-4.76400	0.01300	0.91800	N	-4.76900	-0.71500	0.68000
C	6.33500	0.82600	-0.05700	C	-6.30200	-1.08300	-0.37800	C	-6.84200	-0.57600	-0.28400
C	6.86400	2.09700	-0.29700	C	-6.80800	-2.17000	-1.09700	C	-8.00600	-1.08400	-0.86800

C	6.12100	3.23400	0.03900	C	-6.06900	-3.35500	-1.17800	C	-8.15700	-2.46500	-1.03300
C	4.84700	3.06600	0.62700	C	-4.81900	-3.42900	-0.51900	C	-7.11100	-3.32000	-0.61500
C	4.29800	1.81200	0.87300	C	-4.29200	-2.36200	0.20000	C	-5.94300	-2.84100	-0.03200
C	5.05000	0.68800	0.51400	C	-5.04200	-1.18100	0.26100	C	-5.82200	-1.45800	0.14300
C	7.93000	6.14700	-1.59300	C	-7.79200	-5.43900	-3.84700	C	-11.20000	-2.90800	-3.26400
C	7.70100	7.17500	-0.67500	C	-7.58700	-6.73700	-3.37400	C	-11.73800	-4.10200	-2.77700
C	6.95400	6.91500	0.47500	C	-6.88300	-6.92800	-2.18300	C	-11.10300	-4.76000	-1.72200
C	6.44200	5.63800	0.70700	C	-6.39200	-5.83400	-1.47200	C	-9.94300	-4.22900	-1.15800
C	6.66300	4.59500	-0.20800	C	-6.58900	-4.52200	-1.93600	C	-9.39000	-3.03000	-1.63800
C	7.41500	4.87200	-1.36200	C	-7.29600	-4.34500	-3.13700	C	-10.03800	-2.37900	-2.70100
H	11.73900	-5.49400	-0.94400	H	-11.65100	5.20800	0.96400	H	-7.09200	7.53700	1.69800
H	12.84400	-4.38600	-2.88700	H	-12.68300	4.91900	-1.29000	H	-8.39500	8.08700	-0.35700
H	11.81100	-2.35000	-3.89300	H	-11.61500	3.38400	-2.94100	H	-8.69900	6.35000	-2.12200
H	9.69000	-1.44800	-2.98000	H	-9.53200	2.17500	-2.35400	H	-7.69300	4.10100	-1.84300
H	9.64100	-4.56500	-0.00800	H	-9.59500	3.96300	1.56200	H	-6.12600	5.27400	1.99200
H	7.37900	-4.33800	-0.52400	H	-7.32000	3.89100	1.10400	H	-4.43900	3.90000	1.18900
H	8.82400	-0.31800	-1.12200	H	-8.74700	0.42400	-1.03000	H	-7.94700	2.13200	-0.59300
H	5.25700	-3.49000	0.42600	H	-5.23800	2.69900	1.74900	H	-3.42000	1.66600	1.52200
H	7.86800	2.20300	-0.71500	H	-7.79100	-2.10400	-1.56900	H	-8.80700	-0.40500	-1.17200
H	4.26200	3.95500	0.87600	H	-4.23700	-4.35000	-0.60000	H	-7.21500	-4.39500	-0.78200
H	3.31400	1.71700	1.33300	H	-3.32400	-2.43800	0.69700	H	-5.14300	-3.52200	0.26200
H	8.50500	6.34100	-2.50000	H	-8.33400	-5.27600	-4.78100	H	-11.68300	-2.38900	-4.09400
H	8.10300	8.17400	-0.85600	H	-7.97300	-7.59300	-3.93000	H	-12.64600	-4.51600	-3.21800
H	6.77600	7.70800	1.20400	H	-6.72400	-7.93800	-1.79800	H	-11.51800	-5.69000	-1.32700
H	5.88300	5.43600	1.62400	H	-5.86800	-5.99100	-0.52600	H	-9.46800	-4.73500	-0.31400
H	7.57300	4.08000	-2.09800	H	-7.43600	-3.33400	-3.52800	H	-9.60800	-1.46000	-3.10600
C	3.66400	-2.19600	2.23300	C	-3.68100	0.70300	3.00300	C	-3.62900	-2.25100	2.20100
C	3.58200	-1.21500	1.22200	C	-3.58600	0.30000	1.66500	C	-3.57300	-1.24800	1.22200
C	2.34100	-0.77400	0.76400	C	-2.33400	0.18100	1.04700	C	-2.33700	-0.76700	0.77300
C	1.17800	-1.30800	1.32600	C	-1.18400	0.45600	1.78200	C	-1.16700	-1.28900	1.31600
C	1.23900	-2.29200	2.32100	C	-1.25300	0.87200	3.12200	C	-1.20000	-2.30200	2.28700
C	2.50000	-2.72700	2.76300	C	-2.51900	0.99400	3.71400	C	-2.44700	-2.77500	2.71600
C	0.00500	-2.85900	2.89600	C	-0.02800	1.17500	3.89000	C	0.05600	-2.84500	2.82800
C	-1.17700	-1.31300	1.32700	C	1.18000	0.58400	1.78500	C	1.21000	-1.23400	1.30700
C	-2.34400	-0.78400	0.76900	C	2.34300	0.42400	1.05600	C	2.35200	-0.68800	0.78800
C	-3.58300	-1.23000	1.22900	C	3.58600	0.69400	1.69700	C	3.63900	-1.17600	1.22900
C	-3.65800	-2.21300	2.23800	C	3.63600	1.11700	3.04000	C	3.70200	-2.21200	2.21700
C	-2.49200	-2.73900	2.76500	C	2.46000	1.26800	3.74500	C	2.55800	-2.74000	2.74500
C	-1.23300	-2.29800	2.32300	C	1.18800	1.00800	3.14100	C	1.25700	-2.27800	2.30300
O	-0.00100	-0.83000	0.83900	O	0.00900	0.31200	1.12700	O	0.01100	-0.77200	0.84200
O	0.00700	-3.72000	3.76700	O	-0.07400	1.54400	5.08200	O	0.07600	-3.75800	3.69800
H	4.64400	-2.50700	2.59600	H	-4.66500	0.77600	3.47000	H	-4.60100	-2.59900	2.55500
H	2.26200	-0.03600	-0.03400	H	-2.24800	-0.11700	0.00100	H	-2.27700	-0.00300	-0.00300
H	2.51700	-3.48400	3.54800	H	-2.53900	1.31400	4.75700	H	-2.44900	-3.55600	3.47700
H	-2.26900	-0.04400	-0.02800	H	2.28600	0.09500	0.01900	H	2.27800	0.05900	-0.00300
H	-4.63700	-2.52900	2.60200	H	4.60200	1.32000	3.50700	H	4.68100	-2.52900	2.58100
H	-2.50400	-3.49800	3.55000	H	2.44600	1.59200	4.78600	H	2.56800	-3.50500	3.52000
C	-6.97800	6.89000	0.48600	C	9.60800	-5.66000	1.22600	C	11.38600	-4.53800	-1.26200
C	-7.72400	7.14900	-0.66700	C	10.26300	-5.81800	0.00100	C	12.01600	-3.91800	-2.34400

C	-7.94700	6.12100	-1.58500	C	9.99000	-4.93800	-1.04900	C	11.43900	-2.78200	-2.91700
C	-7.42900	4.84700	-1.35300	C	9.07200	-3.90500	-0.87600	C	10.24300	-2.27200	-2.41300
C	-6.67900	4.57200	-0.19800	C	8.40100	-3.73700	0.35000	C	9.59900	-2.88600	-1.32600
C	-6.46300	5.61500	0.71800	C	8.68300	-4.63300	1.39800	C	10.19000	-4.02700	-0.75900
C	-4.86100	3.04800	0.64100	C	6.30200	-2.82000	1.37800	C	7.31800	-3.21500	-0.32300
C	-6.13400	3.21200	0.05000	C	7.42200	-2.64600	0.53200	C	8.32700	-2.34300	-0.79000
C	-6.87300	2.07400	-0.28800	C	7.60400	-1.40800	-0.14100	C	8.09700	-0.95700	-0.75400
C	-6.34200	0.80300	-0.04800	C	6.68800	-0.39200	0.04800	C	6.89900	-0.47000	-0.24000
C	-5.05700	0.66900	0.52500	C	5.57400	-0.60100	0.90500	C	5.92000	-1.36500	0.24900
C	-4.31000	1.79400	0.88800	C	5.36500	-1.81300	1.57700	C	6.10900	-2.75000	0.18600
N	-4.76800	-0.69100	0.68300	N	4.79900	0.53600	0.94700	N	4.80500	-0.63800	0.68800
C	-6.85600	-0.53900	-0.24600	C	6.55700	0.98100	-0.44800	C	6.35000	0.87000	-0.09400
C	-8.04500	-1.03500	-0.78700	C	7.29300	1.77800	-1.30200	C	6.84700	2.13900	-0.37800
C	-8.24300	-2.41700	-0.88300	C	6.84600	3.09700	-1.58500	C	6.06700	3.27200	-0.08800
C	-7.22000	-3.28400	-0.43600	C	5.65900	3.57400	-0.98200	C	4.80200	3.09500	0.51500
C	-6.02800	-2.81500	0.10700	C	4.90700	2.79100	-0.11400	C	4.29100	1.83700	0.81900
C	-5.86000	-1.43000	0.21300	C	5.36900	1.49300	0.14200	C	5.06600	0.72200	0.47900
C	-11.91700	-2.86000	-1.73200	C	9.73900	4.66400	-3.46100	C	8.40500	6.22800	-0.54000
C	-11.88800	-3.99900	-2.53900	C	9.07700	5.59100	-4.27000	C	7.52400	7.22100	-0.97500
C	-10.66700	-4.62300	-2.80300	C	7.68600	5.70200	-4.20100	C	6.16700	6.92200	-1.11900
C	-9.48600	-4.11100	-2.26600	C	6.96100	4.89600	-3.32700	C	5.69500	5.64200	-0.83000
C	-9.50000	-2.96600	-1.45200	C	7.61400	3.95500	-2.50900	C	6.57000	4.63400	-0.39200
C	-10.73600	-2.35000	-1.19300	C	9.01600	3.85000	-2.59300	C	7.93300	4.94800	-0.25100
H	-6.80400	7.68400	1.21500	H	9.82400	-6.33600	2.05400	H	11.83300	-5.42100	-0.80100
H	-8.12800	8.14700	-0.84800	H	10.98500	-6.62600	-0.13300	H	12.95100	-4.31800	-2.73900
H	-8.52100	6.31400	-2.49400	H	10.48900	-5.06300	-2.01200	H	11.91700	-2.29600	-3.77000
H	-7.58300	4.05500	-2.09000	H	8.84400	-3.24100	-1.71300	H	9.78400	-1.40100	-2.88700
H	-5.90500	5.41400	1.63600	H	8.19700	-4.50000	2.36700	H	9.71900	-4.50300	0.10500
H	-4.28000	3.93700	0.89300	H	6.15400	-3.78500	1.86300	H	7.47600	-4.29200	-0.40300
H	-7.87600	2.17700	-0.71000	H	8.48100	-1.25800	-0.77400	H	8.86200	-0.26500	-1.11500
H	-3.32600	1.70200	1.35000	H	4.49300	-1.94100	2.21800	H	5.32800	-3.44600	0.49500
H	-8.80500	-0.34600	-1.16300	H	8.19400	1.39500	-1.78600	H	7.83000	2.25300	-0.84000
H	-7.38000	-4.36300	-0.50100	H	5.34200	4.59900	-1.18000	H	4.22300	3.97900	0.79000
H	-5.25500	-3.51000	0.43700	H	3.99700	3.15300	0.36400	H	3.33300	1.73000	1.32800
H	-12.86800	-2.36900	-1.51100	H	10.82700	4.57900	-3.50100	H	9.46500	6.45400	-0.41400
H	-12.81300	-4.39900	-2.96000	H	9.64500	6.22600	-4.95300	H	7.89300	8.22300	-1.20100
H	-10.63100	-5.50900	-3.44000	H	7.16100	6.41600	-4.83800	H	5.47100	7.68800	-1.46700
H	-8.53100	-4.58800	-2.50100	H	5.87200	4.97100	-3.30100	H	4.63700	5.41000	-0.97200
H	-10.77000	-1.47600	-0.53900	H	9.54600	3.14900	-1.94500	H	8.62400	4.18600	0.11700

Table S15: Coordinates of the optimized minimum in the S_0, S_1, and T_1 of 16DAX at the MN15/cc-pVDZ level.

S_0				S_1				T_1			
C	6.12400	-3.71200	-0.39000	C	-10.16600	10.02100	5.74300	C	6.19900	-3.71100	-0.36800
C	6.83500	-2.51500	-0.42900	C	-10.74600	9.06300	6.57200	C	6.91000	-2.51400	-0.40900
C	6.21000	-1.26200	-0.37700	C	-10.51200	7.69000	6.41900	C	6.28400	-1.26100	-0.37400

C	4.80600	-1.22800	-0.27800	C	-9.65400	7.27300	5.38200	C	4.87800	-1.22300	-0.29500
C	4.08100	-2.43400	-0.24000	C	-9.06400	8.23900	4.54200	C	4.15400	-2.43200	-0.25400
C	4.73300	-3.66200	-0.29600	C	-9.31700	9.59400	4.72100	C	4.80700	-3.65900	-0.29000
C	7.06800	0.00000	-0.42500	C	-11.19000	6.71100	7.37500	C	7.14300	-0.00000	-0.42300
C	4.80600	1.22800	-0.27800	C	-9.94700	4.92300	5.99500	C	4.87800	1.22300	-0.29500
C	6.21000	1.26200	-0.37700	C	-10.81400	5.26700	7.05100	C	6.28400	1.26100	-0.37400
C	6.83400	2.51500	-0.42900	C	-11.34700	4.23200	7.83100	C	6.91000	2.51400	-0.40800
C	6.12300	3.71200	-0.39000	C	-11.05400	2.89000	7.60200	C	6.19900	3.71100	-0.36800
C	4.73200	3.66200	-0.29600	C	-10.19300	2.56400	6.55300	C	4.80700	3.65900	-0.28900
C	4.08000	2.43400	-0.24000	C	-9.64700	3.56600	5.75900	C	4.15400	2.43200	-0.25400
C	7.89000	0.00000	-1.72900	C	-12.71800	6.87100	7.25900	C	7.96200	0.00000	-1.72900
C	8.02700	0.00000	0.78200	C	-10.75400	7.03300	8.81800	C	8.10700	-0.00000	0.78000
N	4.12600	-0.00000	-0.21600	N	-9.38300	5.91300	5.17800	N	4.19400	-0.00000	-0.25800
H	6.64900	-4.66700	-0.43300	H	-10.37300	11.08100	5.89600	H	6.72500	-4.66700	-0.39700
H	7.92500	-2.55100	-0.50300	H	-11.41100	9.39200	7.37400	H	8.00100	-2.55000	-0.47000
H	2.99300	-2.40700	-0.16700	H	-8.40100	7.91200	3.74100	H	3.06600	-2.39900	-0.19300
H	4.14400	-4.58100	-0.26500	H	-8.84500	10.31700	4.05300	H	4.21800	-4.57800	-0.25600
H	7.92400	2.55100	-0.50300	H	-12.02000	4.49300	8.65200	H	8.00100	2.55000	-0.47000
H	6.64800	4.66700	-0.43400	H	-11.49000	2.11400	8.23300	H	6.72500	4.66700	-0.39700
H	4.14300	4.58100	-0.26600	H	-9.94100	1.52200	6.34400	H	4.21800	4.57800	-0.25600
H	2.99300	2.40700	-0.16700	H	-8.97700	3.30400	4.94000	H	3.06600	2.39900	-0.19300
H	8.53700	0.88900	-1.78400	H	-13.23700	6.19000	7.95100	H	8.60800	0.89000	-1.78600
H	7.22600	0.00000	-2.60500	H	-13.05400	6.64600	6.23600	H	7.29400	0.00000	-2.60400
H	8.53700	-0.88800	-1.78400	H	-13.02400	7.89900	7.50600	H	8.60800	-0.89000	-1.78700
H	8.67600	0.89000	0.76600	H	-11.24100	6.35500	9.53500	H	8.75500	0.88900	0.76200
H	8.67600	-0.88900	0.76600	H	-11.02900	8.06400	9.08900	H	8.75500	-0.89000	0.76200
H	7.46300	0.00000	1.72600	H	-9.66400	6.92600	8.92500	H	7.54500	-0.00000	1.72500
C	1.92300	-0.00000	-1.29400	C	-8.98800	5.26100	2.84500	C	1.98900	0.00000	-1.33500
C	2.70100	-0.00000	-0.12200	C	-8.49300	5.52700	4.12500	C	2.76500	-0.00000	-0.17300
C	2.09900	-0.00000	1.13100	C	-7.12100	5.41800	4.38800	C	2.15000	-0.00000	1.08600
C	0.70200	-0.00000	1.21300	C	-6.25800	5.04200	3.36500	C	0.76200	-0.00000	1.16900
C	-0.09000	-0.00000	0.06100	C	-6.73100	4.77000	2.06800	C	-0.04200	0.00000	0.01200
C	0.54000	-0.00100	-1.19600	C	-8.11100	4.88700	1.82900	C	0.59900	0.00000	-1.23900
C	-1.56700	-0.00000	0.14900	C	-5.80900	4.37600	0.99900	C	-1.49900	0.00000	0.12100
C	-1.19100	0.00000	2.61700	C	-4.01700	4.58500	2.73200	C	-1.13700	-0.00000	2.59300
C	-1.63000	0.00000	3.94800	C	-2.69500	4.51300	3.13000	C	-1.61800	-0.00000	3.89000
C	-2.99000	0.00000	4.21500	C	-1.68800	4.14200	2.20500	C	-3.01200	-0.00000	4.13300
C	-3.91600	0.00000	3.16100	C	-2.04100	3.85200	0.89400	C	-3.89500	-0.00000	3.06100
C	-3.48000	0.00000	1.84400	C	-3.38300	3.93000	0.50800	C	-3.39400	-0.00000	1.75600
C	-2.09400	-0.00000	1.53800	C	-4.43200	4.29800	1.40000	C	-2.00200	-0.00000	1.46500
O	0.16100	0.00000	2.46000	O	-4.92800	4.95000	3.68200	O	0.21900	-0.00000	2.42800
O	-2.26200	-0.00000	-0.85800	O	-6.21400	4.12700	-0.17600	O	-2.25600	0.00000	-0.89900
H	2.42700	-0.00000	-2.26200	H	-10.06100	5.35300	2.66300	H	2.49000	0.00000	-2.30500
H	-0.10400	-0.00000	-2.07600	H	-8.45600	4.67100	0.81700	H	-0.04000	0.00000	-2.12300
H	-4.99100	0.00000	3.34700	H	-1.29100	3.56400	0.15400	H	-4.97700	-0.00000	3.21700
H	2.69900	-0.00000	2.04200	H	-6.72400	5.62300	5.38300	H	2.74600	-0.00000	2.00000
H	-0.87900	0.00100	4.73800	H	-2.45900	4.74900	4.16800	H	-0.89400	-0.00000	4.70600
H	-3.33900	0.00100	5.24900	H	-0.64700	4.08600	2.52200	H	-3.38800	-0.00000	5.15600
C	-5.34600	-3.71100	-0.99500	C	-3.75500	6.75400	-3.62300	C	-5.65600	-3.68200	-0.80400

C	-5.65200	-2.51500	-1.64000	C	-4.24500	5.49900	-3.98600	C	-5.99500	-2.50300	-1.46900
C	-5.36500	-1.26100	-1.08400	C	-4.26400	4.42800	-3.09200	C	-5.56400	-1.25200	-1.02300
C	-4.74800	-1.22600	0.17800	C	-3.77100	4.65600	-1.78400	C	-4.75500	-1.21500	0.13700
C	-4.43600	-2.43000	0.83600	C	-3.28900	5.93300	-1.40800	C	-4.39600	-2.40800	0.80500
C	-4.73000	-3.66000	0.25600	C	-3.27800	6.96900	-2.32500	C	-4.84800	-3.63000	0.33800
C	-5.69300	-0.00000	-1.87800	C	-4.91700	3.11300	-3.47100	C	-5.83400	0.00000	-1.83600
C	-4.74800	1.22600	0.17800	C	-4.07800	2.32500	-1.18700	C	-4.75400	1.21500	0.13700
C	-5.36500	1.26100	-1.08400	C	-4.58000	2.02000	-2.47600	C	-5.56300	1.25200	-1.02300
C	-5.65200	2.51400	-1.64000	C	-4.87800	0.68500	-2.75400	C	-5.99500	2.50300	-1.46900
C	-5.34600	3.71100	-0.99500	C	-4.68700	-0.32500	-1.81000	C	-5.65500	3.68200	-0.80400
C	-4.73000	3.66000	0.25500	C	-4.19700	-0.00900	-0.53800	C	-4.84800	3.63000	0.33800
C	-4.43600	2.43100	0.83600	C	-3.89900	1.30500	-0.22300	C	-4.39500	2.40800	0.80500
C	-7.19100	-0.00000	-2.23700	C	-4.53300	2.69700	-4.89900	C	-7.26400	0.00000	-2.39500
C	-4.84900	-0.00000	-3.16700	C	-6.45000	3.33900	-3.37800	C	-4.81300	-0.00000	-3.00400
N	-4.45300	0.00000	0.80200	N	-3.74700	3.62900	-0.85400	N	-4.30700	0.00000	0.64100
H	-5.58200	-4.66700	-1.46500	H	-3.75700	7.56700	-4.35100	H	-6.01400	-4.64000	-1.18400
H	-6.12200	-2.55200	-2.62600	H	-4.64000	5.36000	-4.99300	H	-6.60100	-2.56700	-2.37300
H	-3.96100	-2.39900	1.81700	H	-2.93000	6.08300	-0.39300	H	-3.76100	-2.35100	1.68700
H	-4.47700	-4.57900	0.78900	H	-2.90300	7.94900	-2.02800	H	-4.56900	-4.54600	0.86000
H	-6.12200	2.55200	-2.62600	H	-5.28900	0.42300	-3.72900	H	-6.60100	2.56700	-2.37400
H	-5.58200	4.66600	-1.46500	H	-4.93100	-1.35700	-2.06700	H	-6.01300	4.64000	-1.18400
H	-4.47600	4.57900	0.78800	H	-4.05400	-0.79000	0.21000	H	-4.56800	4.54600	0.86000
H	-3.96100	2.40000	1.81700	H	-3.52500	1.57000	0.76300	H	-3.76100	2.35100	1.68700
H	-7.45300	0.88700	-2.83400	H	-5.05700	1.77700	-5.19300	H	-7.43500	0.87700	-3.03300
H	-7.81000	-0.00000	-1.32700	H	-3.45000	2.53100	-4.99200	H	-8.01200	0.00000	-1.58800
H	-7.45300	-0.88700	-2.83400	H	-4.83400	3.46500	-5.62400	H	-7.43500	-0.87700	-3.03300
H	-5.07000	0.89100	-3.77600	H	-6.97400	2.40700	-3.64300	H	-4.96500	0.89700	-3.62500
H	-5.07000	-0.89100	-3.77600	H	-6.74800	4.13000	-4.08400	H	-4.96500	-0.89700	-3.62500
H	-3.78000	-0.00000	-2.91200	H	-6.72500	3.63600	-2.35600	H	-3.78700	-0.00000	-2.60900

Table S16: Coordinates of the optimized minimum in the S_0, S_1, and T_1 of 26DAX at the MN15/cc-pVDZ level.

S_0				S_1				T_1			
C	7.03400	-3.71200	-0.22100	C	7.01300	-3.71100	-0.22200	C	7.04300	-3.45600	-1.32000
C	7.72500	-2.51500	-0.39500	C	7.70600	-2.51500	-0.38700	C	7.72300	-2.25300	-1.14700
C	7.11300	-1.26200	-0.25900	C	7.09700	-1.26100	-0.24000	C	7.10800	-1.10400	-0.63400
C	5.74300	-1.22800	0.06400	C	5.72800	-1.22200	0.09000	C	5.74700	-1.18100	-0.28000
C	5.03900	-2.43400	0.24200	C	5.02200	-2.43100	0.25900	C	5.05400	-2.39600	-0.45300
C	5.67800	-3.66200	0.10200	C	5.65700	-3.65800	0.10500	C	5.69500	-3.51900	-0.96600
C	7.94800	-0.00000	-0.46700	C	7.93500	-0.00000	-0.43900	C	7.93100	0.17300	-0.47800
C	5.74300	1.22800	0.06400	C	5.72800	1.22200	0.09000	C	5.72800	1.16300	0.42400
C	7.11300	1.26200	-0.25900	C	7.09700	1.26100	-0.24000	C	7.08900	1.31200	0.09200
C	7.72500	2.51500	-0.39500	C	7.70600	2.51400	-0.38800	C	7.68500	2.56400	0.30000
C	7.03400	3.71200	-0.22100	C	7.01300	3.71100	-0.22200	C	6.98700	3.65200	0.81700
C	5.67800	3.66200	0.10200	C	5.65800	3.65800	0.10400	C	5.63900	3.49000	1.14100
C	5.03900	2.43400	0.24300	C	5.02200	2.43100	0.25800	C	5.01700	2.26100	0.94700

C	8.50700	0.00000	-1.90300	C	8.51400	-0.00000	-1.86700	C	8.47600	0.59200	-1.85700
C	9.11800	-0.00000	0.53600	C	9.09300	-0.00000	0.57800	C	9.11300	-0.10300	0.47300
N	5.07500	-0.00000	0.20600	N	5.06200	0.00000	0.25200	N	5.07600	-0.06500	0.24100
H	7.54800	-4.66700	-0.33700	H	7.52500	-4.66700	-0.34500	H	7.55900	-4.32900	-1.72300
H	8.78700	-2.55100	-0.64900	H	8.76900	-2.55000	-0.64200	H	8.78000	-2.20000	-1.42300
H	3.97900	-2.40700	0.49500	H	3.96300	-2.39700	0.51500	H	4.00100	-2.45300	-0.17700
H	5.10600	-4.58100	0.24600	H	5.08300	-4.57700	0.24300	H	5.13100	-4.44600	-1.08700
H	8.78700	2.55100	-0.64800	H	8.76900	2.55000	-0.64200	H	8.74100	2.68600	0.04500
H	7.54800	4.66700	-0.33600	H	7.52500	4.66700	-0.34500	H	7.48800	4.61000	0.96300
H	5.10600	4.58100	0.24700	H	5.08300	4.57700	0.24300	H	5.06100	4.32200	1.54900
H	3.97900	2.40700	0.49500	H	3.96300	2.39700	0.51500	H	3.96500	2.14100	1.20500
H	9.13100	-0.88900	-2.08000	H	9.14000	-0.89000	-2.03600	H	9.10500	-0.20200	-2.28800
H	9.13100	0.89000	-2.07900	H	9.14000	0.88900	-2.03600	H	9.09200	1.50100	-1.77600
H	7.68900	0.00000	-2.63900	H	7.70500	-0.00000	-2.61200	H	7.64900	0.79500	-2.55400
H	9.75200	0.88800	0.39700	H	9.72800	0.89000	0.44800	H	9.73800	0.79600	0.59100
H	9.75200	-0.88900	0.39700	H	9.72800	-0.89000	0.44800	H	9.75200	-0.90800	0.07900
H	8.74200	-0.00000	1.56900	H	8.70300	-0.00000	1.60700	H	8.74800	-0.40500	1.46500
C	3.31400	-0.00000	1.91200	C	3.26500	0.00000	1.92400	C	3.30300	-0.56500	1.86200
C	3.69100	-0.00000	0.55600	C	3.66800	0.00000	0.58500	C	3.68900	-0.17700	0.57600
C	2.73200	0.00000	-0.45000	C	2.71600	0.00000	-0.44100	C	2.72200	0.10700	-0.39700
C	1.37700	-0.00000	-0.10100	C	1.36200	0.00000	-0.11700	C	1.37500	-0.00300	-0.06600
C	0.98200	0.00000	1.24400	C	0.93200	0.00000	1.22200	C	0.96200	-0.39100	1.22000
C	1.96900	0.00000	2.24500	C	1.90600	0.00000	2.23300	C	1.94800	-0.66900	2.17700
C	-0.45000	0.00000	1.60500	C	-0.50300	0.00000	1.55600	C	-0.47100	-0.50300	1.54800
C	-0.85200	0.00000	-0.86200	C	-0.86600	0.00000	-0.92400	C	-0.86200	0.22500	-0.82500
C	-1.71200	-0.00000	-1.96900	C	-1.69800	0.00000	-2.03100	C	-1.70600	0.56400	-1.87100
C	-3.08300	-0.00000	-1.76000	C	-3.10900	0.00000	-1.86100	C	-3.10400	0.53100	-1.68800
C	-3.61300	0.00000	-0.45500	C	-3.59200	0.00000	-0.55400	C	-3.59700	0.11300	-0.42600
C	-2.75500	0.00000	0.63400	C	-2.78400	0.00000	0.57000	C	-2.76100	-0.24000	0.62400
C	-1.36300	0.00000	0.44300	C	-1.36700	0.00000	0.40600	C	-1.35300	-0.18000	0.46300
O	0.48300	-0.00000	-1.12800	O	0.47900	0.00000	-1.16500	O	0.47200	0.29000	-1.06100
O	-0.84600	0.00100	2.76300	O	-0.92000	0.00000	2.73600	O	-0.87200	-0.85900	2.68200
H	4.09300	-0.00000	2.67600	H	4.02700	0.00000	2.70600	H	4.07600	-0.78200	2.60200
H	1.62600	0.00000	3.28100	H	1.54100	0.00000	3.26200	H	1.60100	-0.97000	3.16700
H	3.02300	-0.00000	-1.50100	H	3.02200	0.00000	-1.48800	H	3.01200	0.41100	-1.40300
H	-1.27900	-0.00000	-2.97000	H	-1.24400	0.00000	-3.02200	H	-1.26100	0.84600	-2.82600
H	-3.77300	-0.00000	-2.60500	H	-3.78700	0.00000	-2.71400	H	-3.79500	0.76900	-2.49600
C	-7.03100	-3.71200	-0.10100	C	-6.96200	-3.69100	-0.06000	C	-6.89000	-3.60900	0.56800
C	-7.73700	-2.51400	-0.01800	C	-7.69900	-2.51100	0.04400	C	-7.64200	-2.44400	0.44000
C	-7.11200	-1.26200	-0.07300	C	-7.09500	-1.25600	-0.04600	C	-7.05700	-1.20600	0.15400
C	-5.71100	-1.22700	-0.21400	C	-5.69100	-1.21200	-0.25200	C	-5.65100	-1.15500	0.01500
C	-4.99200	-2.43500	-0.29800	C	-4.94000	-2.41200	-0.35700	C	-4.89400	-2.34800	0.08000
C	-5.64400	-3.66200	-0.24300	C	-5.57500	-3.63500	-0.26100	C	-5.50600	-3.55600	0.36800
C	-7.96600	-0.00000	0.03000	C	-7.93500	-0.00000	0.08100	C	-7.95400	0.00100	-0.06800
C	-5.71100	1.22700	-0.21400	C	-5.69200	1.21200	-0.25200	C	-5.73000	1.25500	-0.36300
C	-7.11200	1.26100	-0.07300	C	-7.09500	1.25600	-0.04600	C	-7.14300	1.25500	-0.34000
C	-7.73700	2.51400	-0.01900	C	-7.70000	2.51000	0.04400	C	-7.80000	2.47600	-0.52300
C	-7.03100	3.71200	-0.10200	C	-6.96200	3.69100	-0.06000	C	-7.10500	3.67300	-0.68700
C	-5.64400	3.66200	-0.24400	C	-5.57500	3.63500	-0.26100	C	-5.70700	3.67400	-0.62200

C	-4.99200	2.43500	-0.29900	C	-4.94000	2.41200	-0.35700	C	-5.02400	2.48100	-0.44500
C	-8.69900	0.00000	1.38600	C	-8.62500	-0.00000	1.46300	C	-8.81400	0.23600	1.19200
C	-9.00300	-0.00000	-1.11000	C	-9.00700	-0.00000	-1.03200	C	-8.87300	-0.28300	-1.27500
N	-5.02900	0.00000	-0.26900	N	-5.03500	0.00000	-0.35100	N	-5.01100	0.06400	-0.24700
H	-7.55600	-4.66700	-0.05300	H	-7.46800	-4.65400	0.01700	H	-7.38200	-4.55500	0.79700
H	-8.82300	-2.54900	0.09600	H	-8.77700	-2.57100	0.20300	H	-8.72700	-2.50000	0.55300
H	-3.90700	-2.40900	-0.40400	H	-3.86400	-2.34800	-0.50700	H	-3.82200	-2.31100	-0.10300
H	-5.05800	-4.58100	-0.30900	H	-4.99100	-4.55300	-0.34000	H	-4.90100	-4.46200	0.42500
H	-8.82400	2.54900	0.09500	H	-8.77800	2.57100	0.20300	H	-8.89200	2.49200	-0.52700
H	-7.55600	4.66600	-0.05400	H	-7.46900	4.65300	0.01800	H	-7.65300	4.60600	-0.82800
H	-5.05900	4.58100	-0.31000	H	-4.99200	4.55300	-0.33900	H	-5.14900	4.60800	-0.68800
H	-3.90700	2.40900	-0.40500	H	-3.86500	2.34800	-0.50700	H	-3.94000	2.47800	-0.34400
H	-9.33900	-0.89000	1.48300	H	-9.26200	-0.89000	1.57400	H	-9.42700	-0.65000	1.41300
H	-9.33900	0.89000	1.48300	H	-9.26300	0.88900	1.57400	H	-9.49600	1.08600	1.04200
H	-7.97700	0.00000	2.21600	H	-7.88200	-0.00000	2.27300	H	-8.17900	0.44800	2.06400
H	-9.65000	-0.88900	-1.05200	H	-9.64900	-0.88900	-0.94700	H	-9.49800	-1.16800	-1.08600
H	-8.50200	-0.00000	-2.09000	H	-8.54100	-0.00000	-2.02700	H	-8.28000	-0.46300	-2.18400
H	-9.65000	0.88800	-1.05200	H	-9.64900	0.88900	-0.94700	H	-9.54100	0.57200	-1.45900
H	-3.13400	0.00000	1.65700	H	-3.17500	0.00000	1.58800	H	-3.14600	-0.53800	1.60100

Table S17: Coordinates of the optimized minimum in the S_0, S_1, and T_1 of 36DAX at the MN15/cc-pVDZ level.

S_0				S_1				T_1			
C	-6.48600	-3.71200	-0.95700	C	-6.43600	-3.69100	-0.97800	C	7.50600	-2.78000	1.45500
C	-7.08100	-2.51500	-1.34900	C	-7.06600	-2.51000	-1.37300	C	7.77400	-1.41400	1.52900
C	-6.54800	-1.26200	-1.01900	C	-6.55400	-1.25500	-1.04100	C	6.89300	-0.45100	1.02800
C	-5.36100	-1.22800	-0.26400	C	-5.36100	-1.21100	-0.27400	C	5.70600	-0.90000	0.40700
C	-4.75500	-2.43400	0.13700	C	-4.71800	-2.41100	0.12700	C	5.40000	-2.28000	0.39500
C	-5.31200	-3.66200	-0.20600	C	-5.25400	-3.63600	-0.22600	C	6.29800	-3.20900	0.89900
C	-7.26600	0.00000	-1.49200	C	-7.27500	-0.00000	-1.49300	C	7.20400	1.02200	1.24400
C	-5.36100	1.22800	-0.26300	C	-5.36100	1.21100	-0.27400	C	5.03400	1.39400	-0.08500
C	-6.54800	1.26200	-1.01800	C	-6.55400	1.25500	-1.04100	C	6.13600	1.91200	0.63000
C	-7.08100	2.51600	-1.34800	C	-7.06600	2.51000	-1.37300	C	6.27100	3.30100	0.70800
C	-6.48600	3.71200	-0.95500	C	-6.43600	3.69000	-0.97800	C	5.37500	4.17200	0.08500
C	-5.31200	3.66200	-0.20400	C	-5.25400	3.63600	-0.22600	C	4.33200	3.64800	-0.68100
C	-4.75500	2.43400	0.13800	C	-4.71800	2.41100	0.12700	C	4.17000	2.27300	-0.78200
C	-8.70300	0.00000	-0.93300	C	-8.70000	-0.00000	-0.89600	C	8.56100	1.35900	0.59200
C	-7.31900	0.00100	-3.03200	C	-7.36100	-0.00000	-3.03500	C	7.28100	1.29100	2.76100
N	-4.77700	-0.00000	0.09100	N	-4.79900	0.00000	0.10100	N	4.79900	0.01300	-0.15800
H	-6.93400	-4.66700	-1.23600	H	-6.86600	-4.65300	-1.25800	H	8.22100	-3.50000	1.85600
H	-8.00100	-2.55100	-1.93900	H	-7.98600	-2.57000	-1.95900	H	8.69700	-1.08200	2.01000
H	-3.83700	-2.40700	0.72400	H	-3.80200	-2.34300	0.71000	H	4.44000	-2.61100	0.00100
H	-4.82000	-4.58100	0.11900	H	-4.75300	-4.55300	0.08500	H	6.04400	-4.26900	0.87300
H	-8.00100	2.55200	-1.93700	H	-7.98600	2.57000	-1.95900	H	7.11400	3.71700	1.26300
H	-6.93300	4.66800	-1.23300	H	-6.86600	4.65300	-1.25800	H	5.51400	5.25000	0.17200
H	-4.82000	4.58100	0.12100	H	-4.75300	4.55300	0.08400	H	3.65100	4.30900	-1.21800

H	-3.83700	2.40600	0.72600	H	-3.80200	2.34300	0.71000	H	3.38900	1.85800	-1.41700
H	-9.25600	-0.88800	-1.27400	H	-9.25500	-0.89000	-1.22800	H	9.36200	0.73700	1.01900
H	-9.25600	0.88900	-1.27400	H	-9.25500	0.89000	-1.22800	H	8.82400	2.41200	0.76500
H	-8.69100	-0.00000	0.16700	H	-8.66600	-0.00000	0.20300	H	8.52500	1.18400	-0.49400
H	-7.84900	0.89100	-3.40400	H	-7.90300	0.88900	-3.39000	H	7.51000	2.35000	2.95400
H	-7.84900	-0.88900	-3.40500	H	-7.90300	-0.88900	-3.39000	H	8.07300	0.68400	3.22400
H	-6.30300	0.00100	-3.45400	H	-6.35800	-0.00000	-3.48400	H	6.32600	1.04700	3.25000
C	-3.66100	-0.00000	2.27500	C	-3.67600	0.00000	2.29800	C	3.68800	-1.33200	-1.91000
C	-3.58000	-0.00000	0.87000	C	-3.59500	0.00000	0.89300	C	3.62600	-0.45100	-0.78800
C	-2.34800	-0.00000	0.22700	C	-2.33600	0.00000	0.22800	C	2.33800	-0.03400	-0.27400
C	-1.17700	-0.00000	0.99500	C	-1.18600	0.00000	0.99700	C	1.19300	-0.51800	-0.85600
C	-1.23900	-0.00100	2.39500	C	-1.22600	0.00000	2.41700	C	1.23900	-1.39800	-1.98600
C	-2.49600	-0.00100	3.02400	C	-2.51500	0.00000	3.04300	C	2.53800	-1.77900	-2.50100
C	-0.00000	-0.00100	3.20000	C	-0.02500	0.00000	3.20900	C	0.03800	-1.89800	-2.59000
C	1.17700	-0.00000	0.99500	C	1.18100	0.00000	1.00800	C	-1.17700	-0.58400	-0.84400
C	2.34800	-0.00000	0.22700	C	2.35100	0.00000	0.25200	C	-2.35100	-0.15300	-0.22700
C	3.58000	-0.00000	0.87000	C	3.58800	0.00000	0.90500	C	-3.58200	-0.58700	-0.72400
C	3.66100	-0.00000	2.27500	C	3.65300	0.00000	2.30200	C	-3.64300	-1.44200	-1.83000
C	2.49600	-0.00100	3.02400	C	2.47300	0.00000	3.04300	C	-2.46200	-1.86300	-2.43600
C	1.23900	-0.00100	2.39500	C	1.22100	0.00000	2.41200	C	-1.21600	-1.44400	-1.95200
O	-0.00000	-0.00000	0.31100	O	-0.00000	0.00000	0.31500	O	-0.00100	-0.13000	-0.31200
O	0.00000	-0.00100	4.42300	O	-0.00700	0.00000	4.45600	O	0.04200	-2.66400	-3.57800
H	-4.64500	-0.00100	2.74600	H	-4.65500	0.00000	2.78500	H	4.66800	-1.60800	-2.30800
H	-2.50500	-0.00100	4.11500	H	-2.52600	0.00000	4.13300	H	2.54300	-2.42700	-3.37700
H	-2.28400	-0.00000	-0.86200	H	-2.25400	-0.00000	-0.86000	H	2.26700	0.61000	0.60300
H	2.28400	0.00000	-0.86200	H	2.29400	-0.00000	-0.83700	H	-2.29900	0.51200	0.63500
C	6.48600	-3.71200	-0.95700	C	6.46500	-3.71100	-0.98200	C	-6.29800	-2.48300	3.09700
C	7.08100	-2.51500	-1.34800	C	7.05900	-2.51500	-1.37600	C	-6.94600	-1.31800	2.69400
C	6.54800	-1.26200	-1.01900	C	6.53600	-1.26100	-1.03000	C	-6.47900	-0.51700	1.64300
C	5.36100	-1.22800	-0.26300	C	5.36200	-1.22300	-0.25200	C	-5.30500	-0.91800	0.97700
C	4.75500	-2.43400	0.13800	C	4.75800	-2.43100	0.15000	C	-4.64600	-2.09700	1.37900
C	5.31200	-3.66200	-0.20500	C	5.30300	-3.65800	-0.21100	C	-5.13700	-2.86900	2.42700
C	7.26600	0.00000	-1.49200	C	7.25500	-0.00000	-1.50400	C	-7.25400	0.74400	1.26700
C	5.36100	1.22800	-0.26300	C	5.36200	1.22300	-0.25200	C	-5.41200	1.02200	-0.51500
C	6.54800	1.26200	-1.01900	C	6.53600	1.26100	-1.03000	C	-6.59000	1.48100	0.10600
C	7.08100	2.51600	-1.34800	C	7.05900	2.51400	-1.37600	C	-7.16800	2.66400	-0.37200
C	6.48600	3.71200	-0.95600	C	6.46500	3.71100	-0.98200	C	-6.62500	3.39500	-1.42500
C	5.31200	3.66200	-0.20500	C	5.30300	3.65800	-0.21100	C	-5.45700	2.92900	-2.03100
C	4.75500	2.43400	0.13800	C	4.75800	2.43100	0.15000	C	-4.85800	1.75700	-1.58200
C	7.31800	0.00000	-3.03200	C	7.30800	-0.00000	-3.04400	C	-7.31400	1.68100	2.48900
C	8.70300	0.00000	-0.93300	C	8.69200	-0.00000	-0.94600	C	-8.68700	0.34900	0.85800
N	4.77700	0.00000	0.09100	N	4.79200	0.00000	0.12700	N	-4.78700	-0.15700	-0.08200
H	6.93400	-4.66700	-1.23500	H	6.90400	-4.66700	-1.27200	H	-6.69600	-3.07800	3.92100
H	8.00100	-2.55100	-1.93800	H	7.97000	-2.55000	-1.97900	H	-7.85700	-1.01200	3.21600
H	3.83700	-2.40700	0.72500	H	3.85100	-2.39700	0.75400	H	-3.73900	-2.40700	0.86000
H	4.82000	-4.58100	0.12000	H	4.81200	-4.57700	0.11700	H	-4.60500	-3.77700	2.71500
H	8.00100	2.55200	-1.93700	H	7.97000	2.55000	-1.97900	H	-8.08300	3.02500	0.10500
H	6.93300	4.66800	-1.23400	H	6.90400	4.66700	-1.27200	H	-7.10700	4.31200	-1.76700
H	4.81900	4.58100	0.12000	H	4.81200	4.57700	0.11700	H	-5.00500	3.47600	-2.86000

H	3.83700	2.40700	0.72500	H	3.85200	2.39700	0.75400	H	-3.94600	1.39900	-2.06100
H	7.84900	-0.88900	-3.40500	H	7.83900	-0.89000	-3.41800	H	-7.80600	1.18500	3.33900
H	7.84900	0.89000	-3.40500	H	7.83900	0.88900	-3.41800	H	-7.88400	2.59300	2.25500
H	6.30300	0.00000	-3.45400	H	6.29200	-0.00000	-3.46600	H	-6.30100	1.97700	2.80000
H	9.25600	-0.88800	-1.27400	H	9.24400	-0.89000	-1.28700	H	-9.20000	-0.16800	1.68300
H	8.69200	0.00000	0.16600	H	8.67900	-0.00000	0.15400	H	-8.67000	-0.32200	-0.01300
H	9.25500	0.88900	-1.27400	H	9.24400	0.89000	-1.28700	H	-9.27900	1.24000	0.59900
H	2.50500	-0.00100	4.11500	H	2.46700	0.00000	4.13500	H	-2.45700	-2.53100	-3.29900
H	4.64500	-0.00000	2.74600	H	4.63100	0.00000	2.78700	H	-4.61900	-1.76600	-2.19600

Table S18: Coordinates of the optimized minimum in the S_0, S_1, and T_1 of 16BCX at the MN15/cc-pVDZ level.

S_0				S_1				T_1			
C	-4.45000	-1.22200	0.22100	C	4.69400	-0.99800	-0.13400	C	-5.07400	-0.78000	-0.03700
C	-5.80900	-1.60900	0.18500	C	6.06500	-1.35400	-0.10700	C	-6.42100	-1.21400	-0.02200
C	-5.83400	-3.05900	0.19600	C	6.11600	-2.80200	-0.10000	C	-6.39200	-2.66300	-0.01100
C	-4.48800	-3.49000	0.23200	C	4.77400	-3.25200	-0.12300	C	-5.02900	-3.04000	-0.01500
C	-4.07500	0.12600	0.17100	C	4.28800	0.34300	-0.11800	C	-4.74700	0.58000	-0.08200
C	-5.08600	1.07900	0.11900	C	5.27600	1.32000	-0.09400	C	-5.79000	1.50000	-0.07900
C	-6.44500	0.70800	0.08400	C	6.64400	0.97900	-0.06000	C	-7.13600	1.08100	-0.05800
C	-6.81200	-0.63700	0.10800	C	7.04500	-0.35600	-0.06000	C	-7.45800	-0.27500	-0.04200
C	-4.15800	-4.84900	0.28800	C	4.45900	-4.61700	-0.14300	C	-4.64200	-4.38500	0.02800
C	-5.20500	-5.76800	0.28300	C	5.51700	-5.52200	-0.12700	C	-5.65100	-5.34500	0.05500
C	-6.54900	-5.35400	0.22900	C	6.85700	-5.09000	-0.09400	C	-7.01200	-4.98600	0.04400
C	-6.87000	-4.00100	0.19000	C	7.16300	-3.73300	-0.08300	C	-7.38900	-3.64700	0.01500
C	-7.52600	2.70200	-0.97400	C	7.70700	3.00200	0.96400	C	-8.38000	3.04200	-1.00700
C	-8.64700	3.52400	-0.71200	C	8.79500	3.85600	0.66800	C	-9.50800	3.81600	-0.64200
C	-9.27100	3.00500	0.48900	C	9.39400	3.35500	-0.55300	C	-10.00500	3.26400	0.60200
C	-8.50000	1.89300	0.90200	C	8.63900	2.22200	-0.94000	C	-9.15500	2.18200	0.93400
C	-8.94600	4.58700	-1.57400	C	9.08900	4.92800	1.52100	C	-9.92100	4.87100	-1.46600
C	-8.13100	4.80900	-2.68000	C	8.30100	5.12600	2.65100	C	-9.21100	5.13300	-2.63400
C	-7.02800	3.97400	-2.93500	C	7.22900	4.26000	2.93800	C	-8.09800	4.34700	-2.98800
C	-6.71000	2.91100	-2.09200	C	6.91700	3.18800	2.10500	C	-7.66700	3.29300	-2.18600
C	-8.81800	1.16800	2.05600	C	8.94100	1.50300	-2.10400	C	-9.34000	1.43400	2.10300
C	-9.93600	1.57100	2.78400	C	10.02300	1.93500	-2.86700	C	-10.40600	1.78200	2.92900
C	-10.72000	2.66700	2.38100	C	10.79000	3.05400	-2.49000	C	-11.26800	2.84700	2.60800
C	-10.39100	3.38900	1.23700	C	10.47900	3.76900	-1.33800	C	-11.07200	3.59300	1.45000
N	-7.44200	1.71700	0.01000	N	7.61900	2.01500	-0.01600	N	-8.17300	2.05500	-0.04600
N	-3.65100	-2.36900	0.25200	N	3.92000	-2.15200	-0.14600	N	-4.23600	-1.89200	-0.03200
H	-3.02700	0.42800	0.17500	H	3.23000	0.60900	-0.13100	H	-3.70700	0.91000	-0.11000
H	-4.83600	2.14200	0.10200	H	5.00400	2.37800	-0.09900	H	-5.57900	2.57200	-0.08900
H	-7.86800	-0.91400	0.05300	H	8.10800	-0.60500	-0.01300	H	-8.50600	-0.58400	-0.04700
H	-3.12100	-5.18100	0.34500	H	3.42100	-4.95200	-0.17500	H	-3.58900	-4.66900	0.04600
H	-4.97400	-6.83400	0.32500	H	5.29900	-6.59200	-0.14200	H	-5.37700	-6.40200	0.08700
H	-7.34300	-6.10200	0.22400	H	7.66000	-5.82900	-0.07900	H	-7.77500	-5.76600	0.06300
H	-7.91100	-3.67400	0.16200	H	8.20100	-3.39400	-0.06400	H	-8.44400	-3.36300	0.01700

H	-9.80900	5.22800	-1.37800	H	9.92600	5.59400	1.30000	H	-10.79000	5.47400	-1.19300
H	-8.35000	5.63400	-3.36000	H	8.51600	5.95700	3.32600	H	-9.52000	5.95200	-3.28600
H	-6.40700	4.15900	-3.81300	H	6.62900	4.42800	3.83500	H	-7.56100	4.56500	-3.91300
H	-5.85900	2.25900	-2.29900	H	6.09100	2.51300	2.33200	H	-6.80900	2.67900	-2.46500
H	-8.20700	0.32300	2.37800	H	8.34400	0.63900	-2.40000	H	-8.66900	0.61200	2.35700
H	-10.20600	1.02300	3.68900	H	10.28000	1.39200	-3.77900	H	-10.57400	1.21500	3.84700
H	-11.59000	2.95300	2.97400	H	11.63300	3.36200	-3.11200	H	-12.09500	3.09100	3.27800
H	-10.99300	4.24600	0.92600	H	11.06800	4.64200	-1.04600	H	-11.73500	4.42500	1.20200
C	-1.53100	-1.61200	1.20100	C	1.76800	-1.57200	-1.15900	C	-2.13700	-1.13800	0.97300
C	-2.23800	-2.38500	0.25700	C	2.49700	-2.19700	-0.14100	C	-2.81600	-1.86200	-0.01500
C	-1.55200	-3.16400	-0.67400	C	1.83200	-2.86600	0.89700	C	-2.09600	-2.56300	-0.99300
C	-0.15500	-3.16900	-0.65600	C	0.44300	-2.90800	0.89900	C	-0.70800	-2.54600	-0.96600
C	0.56400	-2.39700	0.26100	C	-0.31400	-2.27600	-0.10500	C	-0.00300	-1.81900	0.01600
C	-0.14700	-1.62000	1.19200	C	0.37600	-1.60600	-1.13000	C	-0.74600	-1.11100	0.97800
C	2.04200	-2.39300	0.26400	C	-1.77700	-2.31700	-0.08200	C	1.45000	-1.82300	0.00300
C	1.82600	-3.97200	-1.65700	C	-1.51400	-3.64500	2.02100	C	1.29800	-3.31000	-1.97800
C	2.34300	-4.84400	-2.62000	C	-2.04000	-4.33400	3.09900	C	1.90000	-4.03000	-2.99600
C	3.71800	-4.94600	-2.77100	C	-3.44100	-4.44300	3.26300	C	3.30700	-4.10600	-3.07200
C	4.57400	-4.16200	-1.99000	C	-4.28400	-3.84800	2.33300	C	4.09200	-3.47200	-2.11400
C	4.05800	-3.28900	-1.03500	C	-3.73900	-3.15300	1.24800	C	3.47500	-2.75200	-1.08400
C	2.65700	-3.19300	-0.82700	C	-2.33400	-3.02500	1.03800	C	2.06600	-2.63200	-1.00300
O	0.46600	-3.95100	-1.57700	O	-0.15000	-3.58300	1.93200	O	-0.06700	-3.25200	-1.95100
O	2.67500	-1.78000	1.11100	O	-2.47800	-1.76700	-0.98100	O	2.14900	-1.15900	0.84700
H	-2.08800	-1.03100	1.93700	H	2.30400	-1.06900	-1.96700	H	-2.71000	-0.60900	1.73700
H	-2.08300	-3.75300	-1.42300	H	2.38600	-3.34800	1.70400	H	-2.60800	-3.11600	-1.78200
H	0.43900	-1.04100	1.90700	H	-0.23000	-1.13100	-1.90200	H	-0.19500	-0.55500	1.73700
H	1.64300	-5.42200	-3.22400	H	-1.34300	-4.78300	3.80800	H	1.26000	-4.51800	-3.73200
H	5.65600	-4.20500	-2.12400	H	-5.37000	-3.91200	2.43100	H	5.18200	-3.54000	-2.13800
C	5.06800	-1.11100	-0.32600	C	-5.09800	-1.26500	0.38700	C	5.13300	-1.09600	-0.23100
C	6.17500	-0.70800	0.45300	C	-5.92100	-0.98700	-0.74800	C	5.90000	-0.91800	0.95000
C	6.78100	-1.91700	0.97700	C	-5.92900	-2.20500	-1.54900	C	5.47600	-1.96000	1.87800
C	6.01600	-2.99900	0.48500	C	-5.10500	-3.12400	-0.86700	C	4.47700	-2.70000	1.21700
C	4.27400	-0.18200	-1.00600	C	-4.85600	-0.30600	1.38900	C	5.29600	-0.27700	-1.35500
C	4.59300	1.16400	-0.87400	C	-5.45400	0.93200	1.25000	C	6.23900	0.74000	-1.27800
C	5.69600	1.58200	-0.10100	C	-6.27300	1.22500	0.12800	C	7.00800	0.93400	-0.10900
C	6.49600	0.64900	0.55800	C	-6.50600	0.25600	-0.87800	C	6.84200	0.09900	1.01100
C	6.32200	-4.32400	0.81300	C	-4.85900	-4.40900	-1.33600	C	3.85300	-3.80400	1.79200
C	7.42300	-4.54500	1.63800	C	-5.47300	-4.76900	-2.54100	C	4.25300	-4.15400	3.08300
C	8.19900	-3.48000	2.13000	C	-6.29500	-3.87200	-3.23500	C	5.23900	-3.42500	3.76500
C	7.88200	-2.16400	1.80500	C	-6.53000	-2.57800	-2.74600	C	5.86000	-2.32200	3.16900
C	6.24300	3.83500	-1.05200	C	-7.50100	3.20000	1.04100	C	8.92700	2.25300	-1.02800
C	6.48400	5.13400	-0.54300	C	-7.98100	4.42100	0.51900	C	9.68100	3.37500	-0.61300
C	6.36800	5.04300	0.90000	C	-7.61100	4.45900	-0.88600	C	9.13400	3.80400	0.66000
C	6.05900	3.69400	1.19800	C	-6.92200	3.25800	-1.16400	C	8.07200	2.92300	0.96700
C	6.77500	6.18200	-1.42500	C	-8.69000	5.30100	1.34000	C	10.73900	3.83500	-1.40500
C	6.82600	5.92100	-2.79100	C	-8.92000	4.94500	2.66800	C	11.03500	3.16700	-2.59000
C	6.59500	4.62200	-3.28000	C	-8.46500	3.71300	3.16600	C	10.29100	2.03900	-2.97800
C	6.30300	3.56300	-2.42300	C	-7.75800	2.82000	2.36200	C	9.23300	1.56300	-2.20600
C	5.85200	3.26200	2.51300	C	-6.35300	3.01500	-2.41800	C	7.28900	3.09400	2.11400

C	5.97400	4.20700	3.52900	C	-6.53900	3.97900	-3.40800	C	7.61400	4.14700	2.96700
C	6.29200	5.55000	3.25100	C	-7.25300	5.16100	-3.15600	C	8.68300	5.01600	2.68700
C	6.48700	5.97400	1.94000	C	-7.78300	5.41400	-1.89100	C	9.44200	4.85400	1.53200
N	5.98600	2.96900	0.00900	N	-6.85800	2.48800	0.01200	N	7.95000	1.98000	-0.06200
N	4.97400	-2.50000	-0.30000	N	-4.62300	-2.53200	0.30600	N	4.26000	-2.14900	-0.05700
H	3.42000	-0.50000	-1.60600	H	-4.19100	-0.53900	2.22000	H	4.68200	-0.42400	-2.24400
H	3.98500	1.92600	-1.36600	H	-5.25800	1.72300	1.97300	H	6.37800	1.42700	-2.11400
H	7.35800	0.98700	1.13900	H	-7.16800	0.49300	-1.71300	H	7.46600	0.24900	1.89400
H	5.71700	-5.15200	0.44100	H	-4.20700	-5.08700	-0.78600	H	3.08200	-4.35500	1.25200
H	7.68500	-5.57000	1.91100	H	-5.30400	-5.76700	-2.94900	H	3.78600	-5.01100	3.57200
H	9.05400	-3.69000	2.77400	H	-6.75700	-4.18300	-4.17300	H	5.52700	-3.72500	4.77400
H	8.47800	-1.33400	2.19100	H	-7.16900	-1.88400	-3.29600	H	6.63200	-1.76200	3.70100
H	6.96200	7.18800	-1.04200	H	-9.06400	6.24700	0.94200	H	11.32500	4.70200	-1.09100
H	7.05000	6.72700	-3.49100	H	-9.47000	5.62100	3.32400	H	11.85600	3.51400	-3.22000
H	6.64800	4.43600	-4.35500	H	-8.67400	3.44200	4.20200	H	10.54800	1.52000	-3.90300
H	6.13300	2.55400	-2.80400	H	-7.43700	1.85500	2.75400	H	8.67400	0.67600	-2.50700
H	5.59600	2.22400	2.73000	H	-5.76200	2.12100	-2.61700	H	6.44300	2.44000	2.32900
H	5.81700	3.89700	4.56400	H	-6.10700	3.81300	-4.39700	H	7.01800	4.30100	3.86900
H	6.38200	6.26300	4.07200	H	-7.37900	5.89500	-3.95300	H	8.91000	5.83000	3.37800
H	6.72500	7.01700	1.72000	H	-8.31300	6.34500	-1.68200	H	10.26100	5.53900	1.30100
H	4.13300	-5.62300	-3.51900	H	-3.85200	-4.98900	4.11200	H	3.77600	-4.67100	-3.87700

Table S19: Coordinates of the optimized minimum in the S_0, S_1, and T_1 of 26BCX at the MN15/cc-pVDZ level.

S_0				S_1				T_1			
C	-5.84500	0.21000	-0.76300	C	-5.80200	-0.82800	0.22200	C	-5.82400	-0.20500	0.78800
C	-6.97900	-0.01900	-1.57600	C	-6.93100	-1.41800	0.84400	C	-6.98200	0.04400	1.56200
C	-6.48800	-0.44400	-2.87200	C	-6.42000	-2.34600	1.83100	C	-6.52700	0.49000	2.86300
C	-5.07700	-0.46100	-2.79000	C	-5.00800	-2.27300	1.75900	C	-5.11300	0.49200	2.81900
C	-5.97700	0.59100	0.57800	C	-5.94300	0.14500	-0.77600	C	-5.91300	-0.62300	-0.54500
C	-7.26300	0.75600	1.08300	C	-7.23200	0.51500	-1.14500	C	-7.18200	-0.79500	-1.08800
C	-8.40000	0.56300	0.27400	C	-8.36300	-0.08200	-0.55100	C	-8.34400	-0.57700	-0.32000
C	-8.26300	0.17600	-1.05900	C	-8.22000	-1.05100	0.44100	C	-8.25000	-0.16000	1.00700
C	-4.28600	-0.79300	-3.89500	C	-4.19100	-3.06700	2.57300	C	-4.34700	0.84700	3.93600
C	-4.93900	-1.13200	-5.07800	C	-4.81800	-3.92600	3.47200	C	-5.02700	1.21800	5.09300
C	-6.34200	-1.13800	-5.16900	C	-6.22100	-4.00300	3.56300	C	-6.43400	1.23400	5.14800
C	-7.12300	-0.78900	-4.07200	C	-7.02700	-3.21900	2.74500	C	-7.18900	0.86700	4.03900
C	-10.15700	1.91500	1.44300	C	-10.16500	0.23200	-2.26500	C	-10.07500	-1.95800	-1.49500
C	-11.49100	1.71400	1.86900	C	-11.49100	0.72700	-2.28600	C	-11.39100	-1.76300	-1.97600
C	-11.84900	0.36000	1.49300	C	-11.80400	1.13000	-0.93000	C	-11.74900	-0.39300	-1.66500
C	-10.71500	-0.19900	0.85700	C	-10.65200	0.86300	-0.15200	C	-10.63100	0.18000	-1.01300
C	-12.18000	2.75200	2.50800	C	-12.21000	0.73500	-3.48900	C	-12.06700	-2.82000	-2.59800
C	-11.53400	3.96900	2.70700	C	-11.60100	0.24800	-4.64200	C	-11.42500	-4.04900	-2.72700
C	-10.21100	4.15700	2.26600	C	-10.28500	-0.25000	-4.60100	C	-10.12000	-4.22900	-2.23300
C	-9.50500	3.13900	1.62800	C	-9.55000	-0.26800	-3.41900	C	-9.42700	-3.19300	-1.61100
C	-10.71300	-1.51900	0.39000	C	-10.60500	1.15900	1.21600	C	-10.63200	1.51700	-0.59500

C	-11.87700	-2.26500	0.55800	C	-11.74200	1.71700	1.79400	C	-11.78200	2.26600	-0.83100
C	-13.01800	-1.72000	1.17700	C	-12.90000	1.98000	1.03800	C	-12.90600	1.70800	-1.46800
C	-13.00900	-0.41000	1.64900	C	-12.93600	1.69100	-0.32300	C	-12.89400	0.38200	-1.89000
N	-9.69300	0.75000	0.83000	N	-9.66400	0.31900	-0.96900	N	-9.62100	-0.77500	-0.91300
N	-4.69100	-0.06000	-1.50500	N	-4.64400	-1.35200	0.78100	N	-4.69400	0.06900	1.55700
H	-5.10600	0.73200	1.21900	H	-5.06800	0.60600	-1.23700	H	-5.01900	-0.78900	-1.14800
H	-7.40900	1.03200	2.13000	H	-7.38800	1.28300	-1.90500	H	-7.29700	-1.10000	-2.13000
H	-9.15200	0.02800	-1.67600	H	-9.10700	-1.50700	0.88800	H	-9.15900	0.00400	1.59100
H	-3.19600	-0.77700	-3.84100	H	-3.10400	-3.01100	2.49800	H	-3.25700	0.82500	3.90300
H	-4.34400	-1.39500	-5.95500	H	-4.20400	-4.55600	4.11900	H	-4.45400	1.49900	5.97800
H	-6.81900	-1.41100	-6.11200	H	-6.67700	-4.68700	4.28100	H	-6.93300	1.53200	6.07200
H	-8.21300	-0.77800	-4.14100	H	-8.11600	-3.28100	2.80700	H	-8.28000	0.86800	4.08000
H	-13.21000	2.60600	2.84200	H	-13.23400	1.11500	-3.51600	H	-13.08300	-2.67900	-2.97300
H	-12.05600	4.78800	3.20500	H	-12.14700	0.24800	-5.58700	H	-11.93700	-4.88200	-3.21100
H	-9.72500	5.12200	2.42300	H	-9.82900	-0.63400	-5.51600	H	-9.63700	-5.20300	-2.33600
H	-8.48200	3.29100	1.27800	H	-8.53200	-0.66000	-3.38600	H	-8.41800	-3.33800	-1.22100
H	-9.82800	-1.94800	-0.08100	H	-9.70600	0.96100	1.80200	H	-9.75900	1.95400	-0.10800
H	-11.90200	-3.29700	0.20300	H	-11.73300	1.95800	2.86000	H	-11.80900	3.31100	-0.51500
H	-13.91400	-2.33300	1.28900	H	-13.77400	2.41600	1.52500	H	-13.79100	2.32400	-1.63400
H	-13.88900	0.01300	2.13700	H	-13.83000	1.90000	-0.91500	H	-13.76200	-0.05200	-2.39200
C	-2.94200	1.22500	-0.38300	C	-2.86600	-1.15700	-0.89200	C	-2.87900	-1.25300	0.58700
C	-3.36500	0.04400	-1.03000	C	-3.31100	-0.99500	0.42300	C	-3.34900	-0.04700	1.12200
C	-2.48200	-1.02000	-1.20800	C	-2.44900	-0.47500	1.40300	C	-2.49000	1.05600	1.22900
C	-1.16800	-0.90200	-0.74500	C	-1.14900	-0.13500	1.05300	C	-1.17200	0.92600	0.80700
C	-0.73400	0.25900	-0.09000	C	-0.67900	-0.27700	-0.26700	C	-0.68000	-0.26900	0.25600
C	-1.64200	1.31700	0.08400	C	-1.56500	-0.79100	-1.23000	C	-1.55900	-1.35300	0.14900
C	0.65200	0.37200	0.40000	C	0.69300	0.09500	-0.62600	C	0.72100	-0.36200	-0.18500
C	0.93200	-1.93800	-0.51300	C	0.93200	0.72800	1.78800	C	0.91400	2.03600	0.54300
C	1.71100	-3.07800	-0.75200	C	1.68000	1.21400	2.85000	C	1.64200	3.20800	0.69100
C	3.03400	-3.09600	-0.34000	C	3.02300	1.61400	2.65000	C	2.97800	3.26000	0.28100
C	3.59400	-1.99300	0.33700	C	3.54600	1.50400	1.35900	C	3.57400	2.06900	-0.27600
C	2.81100	-0.87100	0.57900	C	2.80800	1.02600	0.28300	C	2.85000	0.89000	-0.41500
C	1.47800	-0.82800	0.14400	C	1.46500	0.61100	0.47800	C	1.49700	0.82600	-0.01900
O	-0.35600	-1.97400	-0.95200	O	-0.35300	0.35700	2.05900	O	-0.37600	2.04200	0.94200
O	1.08300	1.36500	0.97100	O	1.15200	-0.02500	-1.78700	O	1.19600	-1.42800	-0.66900
H	-3.64100	2.05500	-0.27800	H	-3.54600	-1.57600	-1.63700	H	-3.55600	-2.10700	0.52900
H	-2.80200	-1.94700	-1.68500	H	-2.78600	-0.33000	2.43100	H	-2.84000	2.01000	1.62700
H	-1.27200	2.21300	0.58500	H	-1.17500	-0.89600	-2.24400	H	-1.16000	-2.27600	-0.27300
H	1.26100	-3.92300	-1.27400	H	1.21200	1.26800	3.83300	H	1.15200	4.06700	1.15000
H	3.20200	-0.00000	1.11000	H	3.20500	0.97000	-0.73200	H	3.27300	0.00600	-0.89200
C	5.93100	-1.09800	0.43900	C	5.88500	1.08000	0.63500	C	5.87300	1.10700	-0.41500
C	7.15800	-1.50800	1.01100	C	7.11100	1.81400	0.51600	C	7.12700	1.54900	-0.89400
C	6.90200	-2.75500	1.70600	C	6.82300	3.16000	0.98900	C	6.92600	2.87800	-1.45000
C	5.53000	-3.04600	1.52500	C	5.45600	3.16400	1.34500	C	5.56000	3.19000	-1.28400
C	5.83800	0.06600	-0.33100	C	5.80400	-0.29000	0.28900	C	5.74700	-0.12000	0.24800
C	6.99100	0.82700	-0.49900	C	6.94900	-0.90500	-0.16800	C	6.88400	-0.91200	0.37500
C	8.22100	0.42900	0.06300	C	8.17000	-0.18600	-0.30000	C	8.13500	-0.48800	-0.11500
C	8.31200	-0.74300	0.81300	C	8.24600	1.18700	0.04400	C	8.26200	0.75800	-0.74200
C	4.93900	-4.17400	2.10400	C	4.81300	4.31000	1.81200	C	5.01300	4.36500	-1.80900

C	5.75600	-5.02000	2.85000	C	5.58800	5.46800	1.93200	C	5.88300	5.25600	-2.43900
C	7.12600	-4.75200	3.02600	C	6.94800	5.47800	1.59400	C	7.25100	4.97500	-2.57200
C	7.70400	-3.62000	2.46100	C	7.57900	4.32500	1.11500	C	7.78000	3.77700	-2.09100
C	9.90400	1.59900	-1.37700	C	9.73400	-2.12800	-0.38600	C	9.70400	-1.89600	1.23800
C	11.05200	2.40100	-1.17000	C	10.94000	-2.42400	-1.05800	C	10.88000	-2.63900	0.98800
C	11.22100	2.52300	0.26400	C	11.26200	-1.27100	-1.88500	C	11.17100	-2.50200	-0.42700
C	10.16900	1.79000	0.86200	C	10.24000	-0.31800	-1.68200	C	10.15900	-1.68100	-0.97500
C	11.76700	2.88600	-2.27300	C	11.58900	-3.63600	-0.82500	C	11.52000	-3.30500	2.04000
C	11.33400	2.56100	-3.55500	C	11.03000	-4.53200	0.08800	C	10.98400	-3.21200	3.32200
C	10.19800	1.75200	-3.74300	C	9.85100	-4.21000	0.77700	C	9.82500	-2.45200	3.55800
C	9.46900	1.25800	-2.66300	C	9.19000	-3.00100	0.56100	C	9.17000	-1.78200	2.52700
C	10.02000	1.72100	2.25200	C	10.20100	0.87800	-2.40600	C	10.12000	-1.38200	-2.34200
C	10.95800	2.39000	3.03500	C	11.24700	1.12900	-3.29400	C	11.13200	-1.90200	-3.14600
C	12.01800	3.11300	2.45700	C	12.28900	0.20700	-3.47500	C	12.15600	-2.70500	-2.61200
C	12.15300	3.18600	1.07400	C	12.29600	-1.00500	-2.78200	C	12.17700	-3.01300	-1.25500
N	9.37400	1.23400	-0.14000	N	9.30800	-0.84000	-0.76500	N	9.26900	-1.31600	0.04100
N	4.94700	-2.03700	0.75400	N	4.91600	1.89000	1.12500	N	4.91800	2.11300	-0.64100
H	4.89500	0.37900	-0.78000	H	4.85500	-0.81900	0.35600	H	4.79400	-0.46100	0.65000
H	6.95700	1.75500	-1.07300	H	6.91700	-1.94400	-0.49400	H	6.81300	-1.89000	0.85300
H	9.27600	-1.05000	1.22600	H	9.20300	1.70700	-0.01100	H	9.24200	1.10000	-1.08000
H	3.87400	-4.38100	1.98600	H	3.75400	4.29000	2.06500	H	3.94500	4.57200	-1.74900
H	5.32000	-5.90700	3.31300	H	5.11900	6.38300	2.29500	H	5.48200	6.18400	-2.85000
H	7.73700	-5.43800	3.61600	H	7.52200	6.40000	1.70200	H	7.90200	5.69200	-3.07500
H	8.76500	-3.40200	2.60400	H	8.63600	4.33900	0.84400	H	8.83700	3.53800	-2.22200
H	12.65300	3.50700	-2.12400	H	12.52300	-3.87200	-1.33900	H	12.42800	-3.88200	1.85400
H	11.88000	2.93200	-4.42400	H	11.52200	-5.48700	0.28100	H	11.47000	-3.72600	4.15300
H	9.88000	1.50100	-4.75700	H	9.44400	-4.91300	1.50500	H	9.42700	-2.38100	4.57200
H	8.59400	0.62400	-2.81100	H	8.29500	-2.75000	1.13000	H	8.28000	-1.18300	2.72300
H	9.19500	1.16800	2.70300	H	9.37400	1.58200	-2.30800	H	9.31900	-0.77600	-2.76800
H	10.86500	2.35200	4.12300	H	11.24300	2.05700	-3.86800	H	11.12400	-1.68400	-4.21600
H	12.73500	3.62300	3.10200	H	13.09000	0.43300	-4.18000	H	12.93300	-3.09400	-3.27100
H	12.96900	3.75400	0.62200	H	13.08800	-1.73800	-2.94800	H	12.96200	-3.64800	-0.83900
H	3.66200	-3.96700	-0.54100	H	3.64000	1.96500	3.47700	H	3.59800	4.13100	0.48300

Table S20: Coordinates of the optimized minimum in the S_0, S_1, and T_1 of 36BCX at the MN15/cc-pVDZ level.

S_0				S_1				T_1			
C	-5.82600	0.70500	0.28400	C	5.50000	0.93800	0.12400	C	-5.23400	-0.74400	0.58500
C	-6.82300	1.16200	-0.60800	C	6.43300	0.90100	1.19000	C	-5.99800	-0.13000	1.60500
C	-6.34700	2.41200	-1.16700	C	5.98600	1.87400	2.16500	C	-5.29800	-0.36200	2.85200
C	-5.07800	2.65800	-0.59600	C	4.80500	2.45200	1.64200	C	-4.13600	-1.10300	2.53800
C	-5.95900	-0.51200	0.96200	C	5.62700	0.09500	-0.98800	C	-5.61800	-0.66500	-0.75900
C	-7.11400	-1.25600	0.74300	C	6.70100	-0.78700	-1.01400	C	-6.78800	0.02400	-1.06100
C	-8.13000	-0.79600	-0.11800	C	7.64700	-0.82100	0.03200	C	-7.57700	0.61100	-0.05100
C	-7.99000	0.41400	-0.79600	C	7.52200	0.02400	1.13300	C	-7.18600	0.53600	1.28500
C	-4.35300	3.81700	-0.90000	C	4.11500	3.45800	2.33000	C	-3.25100	-1.53000	3.53400

C	-4.91800	4.71100	-1.80600	C	4.62700	3.86600	3.55800	C	-3.54200	-1.17700	4.85000
C	-6.17200	4.47000	-2.39600	C	5.79700	3.29500	4.09700	C	-4.68500	-0.42400	5.17600
C	-6.89400	3.32500	-2.07700	C	6.48200	2.30100	3.40400	C	-5.57000	-0.01800	4.18300
C	-10.15800	-2.01800	0.70200	C	9.69100	-1.79400	-1.04700	C	-9.84100	0.74600	-1.11500
C	-11.20200	-2.77800	0.12300	C	10.59500	-2.84900	-0.77300	C	-10.84200	1.73200	-1.27600
C	-10.95700	-2.80100	-1.30600	C	10.15700	-3.46000	0.46600	C	-10.35000	2.93400	-0.63200
C	-9.77500	-2.05600	-1.52800	C	9.00600	-2.74700	0.88300	C	-9.07300	2.62200	-0.10900
C	-12.20300	-3.31800	0.94000	C	11.65900	-3.09700	-1.65000	C	-12.02600	1.41500	-1.95400
C	-12.15100	-3.09000	2.31200	C	11.80700	-2.29300	-2.77600	C	-12.19500	0.12600	-2.45100
C	-11.11400	-2.32100	2.87000	C	10.90500	-1.24200	-3.02900	C	-11.19500	-0.84700	-2.27100
C	-10.10600	-1.77300	2.07900	C	9.83900	-0.97600	-2.17300	C	-10.00800	-0.55600	-1.60100
C	-9.23600	-1.90100	-2.81100	C	8.30900	-3.09700	2.04600	C	-8.30000	3.57800	0.56000
C	-9.91500	-2.49600	-3.87200	C	8.79400	-4.16800	2.79200	C	-8.84000	4.85400	0.71000
C	-11.09900	-3.22900	-3.67000	C	9.94300	-4.88000	2.39600	C	-10.11400	5.17700	0.20700
C	-11.62200	-3.38800	-2.39000	C	10.62500	-4.53300	1.23500	C	-10.87100	4.22400	-0.46700
N	-9.29700	-1.58400	-0.30600	N	8.73300	-1.74000	-0.03900	N	-8.77100	1.29300	-0.40600
N	-4.76700	1.61800	0.28900	N	4.51900	1.88000	0.40600	N	-4.10400	-1.33400	1.15700
H	-5.17500	-0.88600	1.62200	H	4.89600	0.12200	-1.79700	H	-5.01200	-1.10500	-1.55100
H	-7.24600	-2.22100	1.23600	H	6.82700	-1.47600	-1.85100	H	-7.11200	0.12600	-2.09900
H	-8.78400	0.76000	-1.46300	H	8.26600	-0.01300	1.93300	H	-7.80400	0.99700	2.05900
H	-3.38700	4.02100	-0.43700	H	3.21200	3.90500	1.91000	H	-2.37000	-2.12600	3.29500
H	-4.37200	5.62200	-2.06000	H	4.11000	4.65000	4.11500	H	-2.86600	-1.49700	5.64500
H	-6.58100	5.19300	-3.10400	H	6.16800	3.63900	5.06300	H	-4.87900	-0.16500	6.21800
H	-7.87400	3.13900	-2.52200	H	7.39400	1.86100	3.81500	H	-6.46600	0.55500	4.43100
H	-13.01300	-3.90700	0.50200	H	12.36100	-3.90900	-1.44700	H	-12.80500	2.17100	-2.08500
H	-12.92300	-3.50500	2.96200	H	12.63000	-2.47400	-3.47000	H	-13.11100	-0.13600	-2.98200
H	-11.09700	-2.14600	3.94800	H	11.04300	-0.61900	-3.91500	H	-11.35100	-1.85500	-2.66100
H	-9.30800	-1.17000	2.51400	H	9.14400	-0.15700	-2.36700	H	-9.23900	-1.31600	-1.45500
H	-8.31400	-1.34100	-2.97100	H	7.41600	-2.54900	2.35000	H	-7.30800	3.33400	0.94300
H	-9.51600	-2.39100	-4.88300	H	8.26900	-4.46100	3.70300	H	-8.25800	5.61900	1.22800
H	-11.60500	-3.68000	-4.52500	H	10.29600	-5.71300	3.00700	H	-10.50600	6.18600	0.34500
H	-12.53600	-3.96500	-2.22900	H	11.51300	-5.08800	0.92100	H	-11.85700	4.47400	-0.86600
C	-3.66000	1.24300	2.43300	C	3.57500	2.64200	-1.72200	C	-3.46000	-3.06700	-0.44000
C	-3.58200	1.49700	1.04800	C	3.39300	2.18300	-0.41400	C	-3.10000	-2.03900	0.45500
C	-2.34300	1.62600	0.42100	C	2.10100	2.01100	0.10600	C	-1.75900	-1.71300	0.65500
C	-1.17800	1.50700	1.18400	C	1.00200	2.31400	-0.69200	C	-0.77400	-2.42400	-0.03600
C	-1.23700	1.24200	2.55900	C	1.15800	2.76800	-2.01300	C	-1.11400	-3.44000	-0.94000
C	-2.49500	1.11100	3.16800	C	2.46100	2.92000	-2.51200	C	-2.47200	-3.74700	-1.13000
C	-0.00000	1.09800	3.35100	C	-0.01200	3.08000	-2.85500	C	-0.06700	-4.17800	-1.67200
C	1.17800	1.50700	1.18400	C	-1.35800	2.40300	-0.86100	C	1.53000	-2.70000	-0.44200
C	2.34300	1.62600	0.42100	C	-2.56900	2.19000	-0.22900	C	2.82600	-2.28100	-0.13100
C	3.58200	1.49700	1.04800	C	-3.76700	2.45200	-0.94800	C	3.90800	-2.89500	-0.76600
C	3.66000	1.24300	2.43300	C	-3.72700	2.92600	-2.27000	C	3.70100	-3.93400	-1.69900
C	2.49500	1.11100	3.16900	C	-2.50500	3.13200	-2.88300	C	2.41100	-4.34600	-1.97900
C	1.23700	1.24200	2.55900	C	-1.27700	2.87300	-2.19900	C	1.30400	-3.73700	-1.35900
O	0.00000	1.64300	0.51300	O	-0.23200	2.13500	-0.12700	O	0.51600	-2.06100	0.20600
O	-0.00000	0.87200	4.55400	O	0.10800	3.48500	-4.03100	O	-0.31600	-5.07500	-2.46900
H	-4.64000	1.17200	2.90700	H	4.58900	2.78100	-2.10300	H	-4.51400	-3.32300	-0.56100
H	-2.26700	1.79900	-0.65300	H	1.94600	1.64300	1.12100	H	-1.46600	-0.90600	1.32700

H	-2.50900	0.91700	4.24200	H	2.54900	3.27900	-3.53900	H	-2.70500	-4.55100	-1.83000
H	2.26700	1.79900	-0.65300	H	-2.58000	1.83000	0.80000	H	2.97600	-1.49800	0.61200
H	4.64000	1.17200	2.90700	H	-4.66200	3.12400	-2.80100	H	4.56000	-4.37800	-2.20200
H	2.50900	0.91800	4.24300	H	-2.42300	3.49700	-3.90700	H	2.20100	-5.14000	-2.69800
C	5.82600	0.70500	0.28400	C	-5.68300	1.02000	-0.30500	C	5.65700	-1.15500	-0.42600
C	6.82300	1.16200	-0.60800	C	-6.90800	1.13900	0.43300	C	6.99200	-1.11700	-0.01600
C	6.34700	2.41200	-1.16700	C	-6.96500	2.52100	0.89000	C	7.39900	-2.48000	0.20000
C	5.07800	2.65800	-0.59600	C	-5.78500	3.13500	0.41500	C	6.27700	-3.30500	-0.08800
C	5.95900	-0.51200	0.96200	C	-5.29100	-0.19300	-0.91600	C	4.92000	0.03000	-0.82100
C	7.11400	-1.25500	0.74300	C	-6.14100	-1.27200	-0.79900	C	5.60400	1.26700	-0.73000
C	8.13000	-0.79600	-0.11800	C	-7.36100	-1.17000	-0.07200	C	6.91400	1.34500	-0.28700
C	7.99000	0.41400	-0.79600	C	-7.74100	0.04500	0.55300	C	7.68100	0.12200	0.09100
C	4.35400	3.81700	-0.89900	C	-5.48100	4.47000	0.66300	C	6.31000	-4.69100	0.07400
C	4.91800	4.71100	-1.80600	C	-6.40900	5.20000	1.41400	C	7.51100	-5.25900	0.49800
C	6.17200	4.47000	-2.39600	C	-7.58700	4.61000	1.89400	C	8.64200	-4.46000	0.76600
C	6.89400	3.32500	-2.07700	C	-7.87700	3.26400	1.63800	C	8.59700	-3.07700	0.63000
C	10.15800	-2.01800	0.70200	C	-8.51400	-3.16700	-1.02200	C	7.61900	3.55400	-1.24400
C	11.20200	-2.77800	0.12300	C	-9.40100	-4.15000	-0.53100	C	8.49200	4.59100	-0.84400
C	10.95700	-2.80100	-1.30600	C	-9.63200	-3.85200	0.87500	C	9.02900	4.21600	0.45200
C	9.77500	-2.05600	-1.52800	C	-8.87800	-2.69900	1.18300	C	8.45500	2.96800	0.78400
C	12.20300	-3.31800	0.94000	C	-9.89800	-5.13300	-1.38600	C	8.70300	5.69100	-1.68000
C	12.15200	-3.09000	2.31200	C	-9.51000	-5.11400	-2.72700	C	8.04900	5.73800	-2.91000
C	11.11400	-2.32100	2.87000	C	-8.65800	-4.11000	-3.21200	C	7.20500	4.68800	-3.30800
C	10.10600	-1.77300	2.07900	C	-8.15500	-3.11500	-2.37300	C	6.97900	3.58300	-2.48800
C	9.23600	-1.90200	-2.81100	C	-8.80500	-2.19900	2.48700	C	8.70300	2.35200	2.01700
C	9.91500	-2.49600	-3.87200	C	-9.55900	-2.84200	3.46800	C	9.59300	2.98400	2.88500
C	11.09800	-3.22900	-3.67000	C	-10.34400	-3.96500	3.16600	C	10.19500	4.21000	2.55400
C	11.62200	-3.38800	-2.39000	C	-10.37500	-4.48600	1.87100	C	9.90800	4.83800	1.34300
N	9.29700	-1.58400	-0.30600	N	-8.19600	-2.27900	0.02400	N	7.60300	2.56100	-0.25100
N	4.76700	1.61800	0.28900	N	-5.02900	2.20200	-0.30800	N	5.22100	-2.46800	-0.47000
H	5.17500	-0.88600	1.62200	H	-4.33600	-0.25500	-1.43600	H	3.89600	-0.02500	-1.18000
H	7.24600	-2.22100	1.23600	H	-5.85900	-2.24000	-1.21300	H	5.07800	2.19000	-0.97800
H	8.78400	0.76000	-1.46300	H	-8.70300	0.11300	1.06300	H	8.76200	0.18300	0.20000
H	3.38700	4.02100	-0.43700	H	-4.55900	4.90800	0.28000	H	5.43000	-5.30700	-0.11300
H	4.37200	5.62200	-2.06000	H	-6.21000	6.25000	1.63100	H	7.57400	-6.34100	0.62700
H	6.58100	5.19300	-3.10400	H	-8.28800	5.20900	2.47600	H	9.56600	-4.93900	1.09500
H	7.87400	3.13900	-2.52200	H	-8.79700	2.81200	2.01400	H	9.46900	-2.46200	0.85900
H	13.01200	-3.90700	0.50200	H	-10.58500	-5.89500	-1.01400	H	9.37800	6.49400	-1.37400
H	12.92300	-3.50500	2.96200	H	-9.88500	-5.87600	-3.41000	H	8.20000	6.59000	-3.57400
H	11.09700	-2.14600	3.94800	H	-8.38700	-4.09700	-4.26900	H	6.71500	4.73400	-4.28200
H	9.30800	-1.17000	2.51400	H	-7.52400	-2.32100	-2.77100	H	6.33700	2.76500	-2.81600
H	8.31300	-1.34100	-2.97100	H	-8.16500	-1.35400	2.74500	H	8.20700	1.42100	2.29200
H	9.51600	-2.39100	-4.88300	H	-9.52500	-2.47000	4.49300	H	9.81100	2.52200	3.84900
H	11.60500	-3.68000	-4.52500	H	-10.92000	-4.44800	3.95700	H	10.88000	4.68100	3.26100
H	12.53600	-3.96500	-2.22900	H	-10.95800	-5.38000	1.64200	H	10.35400	5.80400	1.09400

Table S21: Coordinates of the optimized minimum in the S_0, S_1, and T_1 of 16PCP at the MN15/cc-pVDZ level.

S_0				S_1				T_1			
C	7.94300	6.80600	1.13300	C	-7.53000	6.83700	-1.15400	C	-8.81400	6.61900	-0.71800
C	9.10800	6.62700	1.88200	C	-8.68300	6.69900	-1.92900	C	-10.00200	6.35700	-1.40200
C	9.42700	5.35500	2.36300	C	-9.02900	5.44000	-2.42600	C	-10.23500	5.07600	-1.90800
C	8.58600	4.27300	2.09900	C	-8.22800	4.33200	-2.15200	C	-9.28700	4.06800	-1.73200
C	7.41200	4.44000	1.34600	C	-7.06700	4.45500	-1.37100	C	-8.08900	4.31800	-1.04400
C	7.10500	5.72400	0.86800	C	-6.73200	5.72700	-0.87700	C	-7.86800	5.61000	-0.53900
C	5.11800	3.44400	1.05900	C	-4.81000	3.39100	-1.03500	C	-5.70100	3.52900	-0.92300
C	6.52300	3.28400	1.06300	C	-6.21900	3.27100	-1.07500	C	-7.08300	3.24000	-0.85300
C	7.05800	2.02100	0.78800	C	-6.79800	2.02300	-0.82700	C	-7.48500	1.92500	-0.59400
C	6.20400	0.94500	0.53000	C	-5.98400	0.92000	-0.55300	C	-6.52400	0.92600	-0.42000
C	4.80400	1.12900	0.55700	C	-4.57700	1.06500	-0.53800	C	-5.15000	1.24000	-0.51800
C	4.24800	2.38800	0.80700	C	-3.97800	2.30900	-0.76800	C	-4.72600	2.55100	-0.75700
N	4.17800	-0.08500	0.24800	N	-3.99300	-0.16300	-0.22800	N	-4.40000	0.08100	-0.28800
C	6.43900	-0.44500	0.18300	C	-6.26300	-0.46600	-0.23000	C	-6.61000	-0.48900	-0.11000
C	7.60100	-1.20500	0.02600	C	-7.44500	-1.20300	-0.11000	C	-7.68600	-1.36100	0.08600
C	7.50500	-2.56700	-0.28300	C	-7.38700	-2.56800	0.18800	C	-7.44600	-2.71500	0.34400
C	6.22300	-3.14600	-0.41900	C	-6.12100	-3.17700	0.35700	C	-6.10900	-3.17400	0.39100
C	5.05200	-2.40900	-0.27000	C	-4.93100	-2.46600	0.24700	C	-5.02200	-2.32600	0.20100
C	5.17200	-1.04600	0.01900	C	-5.01200	-1.09800	-0.04000	C	-5.28500	-0.97300	-0.03800
C	11.01900	-3.63900	-1.24200	C	-10.95100	-3.57200	1.03400	C	-10.78300	-4.14100	1.46100
C	11.05300	-4.95500	-0.77600	C	-10.99800	-4.88500	0.56100	C	-10.72000	-5.44300	0.95800
C	9.92500	-5.49100	-0.15000	C	-9.86200	-5.44200	-0.03200	C	-9.58500	-5.85300	0.25500
C	8.77500	-4.71800	0.00900	C	-8.69200	-4.69100	-0.15300	C	-8.52400	-4.96900	0.05700
C	8.72800	-3.39300	-0.45500	C	-8.63100	-3.37000	0.32000	C	-8.57400	-3.65800	0.55800
C	9.86800	-2.86800	-1.08400	C	-9.77900	-2.82400	0.91700	C	-9.72000	-3.26000	1.26400
H	7.68900	7.79500	0.74500	H	-7.25400	7.81400	-0.75200	H	-8.62400	7.61500	-0.31000
H	9.76500	7.47400	2.09000	H	-9.30900	7.56700	-2.14500	H	-10.74300	7.14700	-1.54100
H	10.33000	5.20500	2.95700	H	-9.92300	5.32100	-3.04100	H	-11.15700	4.86200	-2.45300
H	8.82600	3.28700	2.50300	H	-8.48900	3.35600	-2.56800	H	-9.46300	3.07600	-2.15500
H	6.20900	5.86800	0.25800	H	-5.84700	5.83800	-0.24600	H	-6.95300	5.81600	0.02200
H	4.70100	4.42800	1.28500	H	-4.36000	4.36400	-1.24500	H	-5.38900	4.55300	-1.13800
H	8.14100	1.88300	0.74500	H	-7.88600	1.91600	-0.81600	H	-8.54800	1.68700	-0.49600
H	3.16900	2.54700	0.80700	H	-2.89400	2.42900	-0.74300	H	-3.66700	2.80700	-0.81200
H	8.58100	-0.74600	0.17600	H	-8.41100	-0.72000	-0.28100	H	-8.71100	-0.99200	0.00700
H	6.15000	-4.20600	-0.67200	H	-6.07900	-4.23900	0.60700	H	-5.92400	-4.22800	0.60500
H	4.07800	-2.88900	-0.37500	H	-3.96800	-2.96100	0.38100	H	-4.00300	-2.71300	0.23800
H	11.89200	-3.21300	-1.74000	H	-11.82900	-3.13000	1.50900	H	-11.66200	-3.81200	2.02000
H	11.95300	-5.56000	-0.90000	H	-11.91400	-5.47200	0.65500	H	-11.55100	-6.13400	1.11300
H	9.94300	-6.51600	0.22600	H	-9.88900	-6.46500	-0.41400	H	-9.52800	-6.86500	-0.15000
H	7.90700	-5.13600	0.52500	H	-7.81800	-5.12500	-0.64400	H	-7.65200	-5.29000	-0.51900
H	9.83900	-1.84800	-1.47700	H	-9.74100	-1.80800	1.31600	H	-9.76400	-2.25200	1.68500
C	2.25100	-0.94300	-0.94900	C	-2.09700	-1.02000	1.02900	C	-2.33200	-0.60300	0.78400
C	0.87900	-1.17500	-1.01100	C	-0.73100	-1.30100	1.12700	C	-0.94200	-0.69700	0.76800
C	0.05500	-0.76200	0.03600	C	0.11400	-0.95200	0.06800	C	-0.22100	-0.20000	-0.32000
C	0.58000	-0.08600	1.13800	C	-0.38500	-0.31400	-1.06700	C	-0.87400	0.43200	-1.38000
C	1.95000	0.13500	1.21600	C	-1.74800	-0.05900	-1.18100	C	-2.26000	0.52100	-1.37700

C	2.78700	-0.30500	0.17500	C	-2.60200	-0.42100	-0.12900	C	-2.99000	-0.00800	-0.29800
S	-1.63200	-1.27600	0.02700	S	1.82300	-1.38900	0.13800	S	1.50300	-0.55800	-0.40200
C	-3.19100	-0.76100	-2.25800	C	3.37000	-1.34200	2.40400	C	3.09200	0.25600	1.78600
C	-1.94000	-1.11600	-1.72000	C	2.02500	-1.26900	1.85300	C	1.89000	-0.42000	1.31900
C	-0.88000	-1.44200	-2.58000	C	1.00900	-1.84200	2.68500	C	0.93600	-0.81900	2.21500
C	-1.05800	-1.41900	-3.96400	C	1.26000	-2.31800	3.94800	C	1.15200	-0.69900	3.64600
C	-2.29400	-1.05600	-4.49000	C	2.59900	-2.34400	4.46200	C	2.25300	0.07200	4.10700
C	-3.35500	-0.72300	-3.64500	C	3.63200	-1.85000	3.67500	C	3.16400	0.59900	3.22800
O	-1.66000	-2.71600	0.32900	O	2.00200	-2.76400	-0.40000	O	1.61600	-1.97500	-0.79400
O	-2.41500	-0.32100	0.82100	O	2.60400	-0.32800	-0.55100	O	2.20000	0.48300	-1.17300
O	0.37900	-1.76200	-2.14000	O	-0.30700	-1.89700	2.26600	O	-0.32900	-1.21700	1.87400
H	2.88400	-1.23200	-1.78900	H	-2.75000	-1.26200	1.86800	H	-2.88500	-0.95800	1.65400
H	-0.10000	0.23000	1.93000	H	0.32000	-0.03700	-1.85400	H	-0.27500	0.81700	-2.20800
H	2.39000	0.62200	2.08700	H	-2.16400	0.40400	-2.07700	H	-2.79500	0.97300	-2.21300
H	-0.20800	-1.67300	-4.59800	H	0.41300	-2.66200	4.54100	H	0.40100	-1.11400	4.31500
C	-7.43800	6.70300	-0.31900	C	6.01000	6.87600	0.89600	C	9.25400	6.08700	-0.36300
C	-8.82500	6.60700	-0.18700	C	7.36300	7.06400	0.60400	C	10.57900	5.66200	-0.24300
C	-9.43900	5.35700	-0.28300	C	8.20900	5.95700	0.50500	C	10.84700	4.34000	0.12000
C	-8.67300	4.21400	-0.51100	C	7.70500	4.67200	0.69300	C	9.79800	3.45300	0.36200
C	-7.27700	4.29400	-0.64400	C	6.34600	4.46800	0.99200	C	8.46100	3.86800	0.24600
C	-6.67300	5.55900	-0.54400	C	5.50600	5.59100	1.09000	C	8.20600	5.19900	-0.12100
C	-5.34600	3.11800	-1.74800	C	4.78900	2.86700	2.13700	C	6.16200	3.36000	1.13000
C	-6.46300	3.07400	-0.88200	C	5.81300	3.10200	1.19700	C	7.34800	2.91900	0.50200
C	-6.78700	1.86600	-0.25200	C	6.32500	2.01900	0.43700	C	7.45200	1.57500	0.10400
C	-6.01200	0.72800	-0.48900	C	5.80900	0.75200	0.63100	C	6.39900	0.70000	0.35500
C	-4.90400	0.80500	-1.36200	C	4.78700	0.55200	1.59000	C	5.23900	1.16400	1.01000
C	-4.55800	1.99900	-1.99900	C	4.26100	1.59500	2.35100	C	5.09300	2.50300	1.38000
N	-4.29100	-0.44600	-1.42900	N	4.43100	-0.78500	1.61700	N	4.31600	0.11900	1.13100
C	-6.07100	-0.64100	-0.00900	C	6.05500	-0.56100	0.03000	C	6.16400	-0.70500	0.04600
C	-6.93900	-1.32100	0.85000	C	6.88600	-1.02900	-0.96400	C	6.93900	-1.68300	-0.56900
C	-6.73600	-2.68100	1.10600	C	6.81700	-2.40000	-1.34400	C	6.43400	-2.98800	-0.69400
C	-5.65400	-3.34300	0.48300	C	5.89800	-3.25900	-0.69700	C	5.15600	-3.28100	-0.17100
C	-4.77700	-2.69100	-0.37800	C	5.05900	-2.81300	0.31200	C	4.36900	-2.32200	0.45900
C	-4.99500	-1.33100	-0.60800	C	5.15800	-1.45900	0.67500	C	4.87700	-1.02200	0.53900
C	-9.87400	-3.85400	2.89800	C	9.79100	-2.86100	-3.64200	C	9.38300	-5.09200	-1.82700
C	-9.36300	-4.82500	3.76200	C	9.33800	-3.88200	-4.48200	C	8.75500	-6.05600	-2.62000
C	-7.99300	-5.09600	3.75600	C	8.06000	-4.41600	-4.29600	C	7.36800	-6.01500	-2.78300
C	-7.14200	-4.40200	2.89600	C	7.24300	-3.93900	-3.27500	C	6.61600	-5.02200	-2.15700
C	-7.64300	-3.42300	2.02100	C	7.68400	-2.90800	-2.42300	C	7.23500	-4.04700	-1.35700
C	-9.02300	-3.16200	2.03600	C	8.97100	-2.37400	-2.62800	C	8.63000	-4.09900	-1.20200
H	-6.94600	7.67400	-0.23700	H	5.33900	7.73400	0.96300	H	9.03400	7.11600	-0.65600
H	-9.42400	7.50300	-0.01000	H	7.75700	8.07100	0.45400	H	11.40000	6.35600	-0.43300
H	-10.52400	5.27000	-0.19000	H	9.27000	6.09600	0.28700	H	11.87800	3.99800	0.22400
H	-9.16300	3.24200	-0.61400	H	8.38000	3.81400	0.64100	H	10.01500	2.42800	0.67300
H	-5.58500	5.63800	-0.61700	H	4.44200	5.45000	1.28800	H	7.17400	5.53300	-0.24500
H	-5.11100	4.05700	-2.25300	H	4.41900	3.70400	2.73100	H	6.08800	4.40500	1.43800
H	-7.62800	1.82100	0.44400	H	7.09300	2.20100	-0.31800	H	8.33900	1.23200	-0.43400
H	-3.70100	2.05300	-2.67400	H	3.47000	1.40500	3.07700	H	4.17000	2.86500	1.83500
H	-7.75700	-0.78700	1.33900	H	7.55800	-0.35200	-1.49500	H	7.92400	-1.43400	-0.97000

H	-5.51400	-4.41000	0.66800	H	5.86100	-4.30800	-0.99100	H	4.78200	-4.30500	-0.23700
H	-3.94100	-3.22200	-0.83400	H	4.32600	-3.46000	0.79200	H	3.39100	-2.58300	0.85900
H	-10.94500	-3.63900	2.88700	H	10.79200	-2.44500	-3.77600	H	10.46600	-5.12000	-1.68600
H	-10.02900	-5.36700	4.43600	H	9.98000	-4.25900	-5.28100	H	9.34400	-6.83400	-3.11000
H	-7.58100	-5.84600	4.43300	H	7.69500	-5.20400	-4.95700	H	6.86900	-6.75800	-3.40800
H	-6.06800	-4.59900	2.91800	H	6.23400	-4.34000	-3.15700	H	5.53500	-4.98000	-2.31200
H	-9.43400	-2.42400	1.34200	H	9.34400	-1.59400	-1.96000	H	9.12600	-3.36600	-0.56100
H	-2.43400	-1.03200	-5.57100	H	2.80000	-2.74900	5.45200	H	2.34900	0.27200	5.17600
H	-4.33300	-0.44600	-4.04200	H	4.66200	-1.84700	4.04000	H	4.01100	1.18900	3.58100

Table S22: Coordinates of the optimized minimum in the S_0, S_1, and T_1 of 26PCP at the MN15/cc-pVDZ level.

S_0				S_1				T_1			
C	11.78800	-4.27300	1.19300	C	-9.46600	-5.94900	1.77100	C	-11.78000	-3.34400	-2.89000
C	12.80300	-3.71700	0.41100	C	-10.79200	-5.56800	1.99400	C	-12.80100	-3.17000	-1.95200
C	12.50900	-2.64500	-0.43500	C	-11.14100	-4.21600	1.95300	C	-12.51300	-2.56100	-0.72900
C	11.21200	-2.13600	-0.50000	C	-10.17200	-3.25100	1.68800	C	-11.21600	-2.13200	-0.44600
C	10.18200	-2.68500	0.28300	C	-8.83100	-3.61900	1.46700	C	-10.18100	-2.29800	-1.38100
C	10.49200	-3.76100	1.13000	C	-8.49400	-4.98400	1.51400	C	-10.48500	-2.91200	-2.60700
C	7.68500	-2.99900	0.28800	C	-6.46900	-2.78500	1.62000	C	-7.68300	-2.58400	-1.50500
C	8.80300	-2.13600	0.21700	C	-7.80200	-2.59600	1.19100	C	-8.80300	-1.83300	-1.07900
C	8.58700	-0.76000	0.09300	C	-8.15000	-1.41300	0.48200	C	-8.58900	-0.64200	-0.37700
C	7.28100	-0.26400	0.03200	C	-7.17600	-0.47000	0.22100	C	-7.28400	-0.22400	-0.10000
C	6.18400	-1.15200	0.08400	C	-5.84700	-0.69200	0.67000	C	-6.18500	-1.00600	-0.51900
C	6.37600	-2.53000	0.22800	C	-5.47400	-1.84700	1.37300	C	-6.37600	-2.18900	-1.24100
N	4.99600	-0.41400	0.02700	N	-5.04500	0.36400	0.30400	N	-4.99900	-0.36400	-0.14100
C	6.73200	1.07700	-0.05800	C	-7.15000	0.81900	-0.47600	C	-6.73900	0.95000	0.55600
C	7.31600	2.34300	-0.15700	C	-8.09400	1.58700	-1.12900	C	-7.32800	2.05600	1.17500
C	6.50400	3.47700	-0.27000	C	-7.71100	2.83300	-1.69400	C	-6.52200	3.03700	1.76300
C	5.10100	3.31200	-0.29100	C	-6.37100	3.26400	-1.58300	C	-5.11800	2.87500	1.72900
C	4.49600	2.06200	-0.19100	C	-5.40300	2.50300	-0.93300	C	-4.50800	1.78400	1.11600
C	5.32600	0.94400	-0.06000	C	-5.81000	1.28100	-0.38500	C	-5.33200	0.82700	0.51400
C	8.81800	6.44000	0.26400	C	-10.70600	3.86900	-3.77100	C	-8.84700	5.95100	2.47600
C	8.25000	7.40100	-0.57400	C	-10.62300	5.26200	-3.70700	C	-8.29400	6.47900	3.64500
C	7.10900	7.07900	-1.31300	C	-9.58800	5.86100	-2.98400	C	-7.16000	5.88200	4.19900
C	6.54300	5.80800	-1.21400	C	-8.64100	5.07400	-2.33300	C	-6.58500	4.76600	3.59100
C	7.10400	4.83300	-0.37400	C	-8.71400	3.67000	-2.38700	C	-7.12900	4.22600	2.41500
C	8.25000	5.17000	0.36500	C	-9.76400	3.08000	-3.11400	C	-8.26800	4.83800	1.86700
H	12.00800	-5.10500	1.86400	H	-9.18700	-7.00400	1.79000	H	-11.99600	-3.81100	-3.85300
H	13.81700	-4.11700	0.46100	H	-11.55200	-6.32500	2.19800	H	-13.81500	-3.50800	-2.17300
H	13.29200	-2.20900	-1.05800	H	-12.17200	-3.91000	2.13800	H	-13.30100	-2.42900	0.01600
H	10.98300	-1.31700	-1.18500	H	-10.45000	-2.19500	1.68800	H	-10.99200	-1.68300	0.52500
H	9.71000	-4.18400	1.76600	H	-7.46600	-5.29400	1.31500	H	-9.69900	-3.02600	-3.35800
H	7.85600	-4.07400	0.37300	H	-6.22000	-3.68200	2.18900	H	-7.85200	-3.51900	-2.04400
H	9.43500	-0.07100	0.07600	H	-9.17200	-1.27600	0.12300	H	-9.43800	-0.02500	-0.07300
H	5.53500	-3.22100	0.29200	H	-4.44500	-1.97900	1.70700	H	-5.53400	-2.78700	-1.59100

H	8.40400	2.44400	-0.17800	H	-9.13500	1.26200	-1.18800	H	-8.41700	2.13900	1.22600
H	4.46900	4.19900	-0.36700	H	-6.08100	4.20700	-2.04700	H	-4.48900	3.64600	2.17800
H	3.41000	1.96800	-0.21700	H	-4.36400	2.81900	-0.84900	H	-3.42200	1.68700	1.11200
H	9.70500	6.68300	0.85300	H	-11.50500	3.39300	-4.34300	H	-9.72800	6.41600	2.02800
H	8.69300	8.39600	-0.65200	H	-11.36200	5.88000	-4.21900	H	-8.74500	7.35200	4.12100
H	6.66100	7.82000	-1.97900	H	-9.52200	6.94800	-2.92000	H	-6.72500	6.28100	5.11800
H	5.66700	5.55600	-1.81700	H	-7.85200	5.55000	-1.74800	H	-5.71400	4.28800	4.04700
H	8.68400	4.43300	1.04500	H	-9.82200	1.99200	-3.19400	H	-8.68800	4.44800	0.93600
C	2.74300	-0.43100	0.93300	C	-2.72500	-0.21300	-0.29500	C	-2.74500	0.02200	-0.95900
C	1.45100	-0.95700	0.94300	C	-1.46100	-0.49000	0.16100	C	-1.45500	-0.44700	-1.20800
C	1.12900	-2.02000	0.09400	C	-1.03900	-0.11800	1.47000	C	-1.13600	-1.77900	-0.92700
C	2.07000	-2.52800	-0.80500	C	-1.95800	0.57300	2.32900	C	-2.07600	-2.63100	-0.34300
C	3.35100	-1.99200	-0.83600	C	-3.23500	0.83700	1.90400	C	-3.35300	-2.16200	-0.06900
C	3.69200	-0.95200	0.04900	C	-3.64200	0.47000	0.58400	C	-3.69400	-0.83700	-0.39800
S	-0.43500	-2.81100	0.29100	S	0.36100	-0.89100	2.11000	S	0.41700	-2.40900	-1.47400
C	-2.73400	-1.31800	0.35500	C	2.74600	-0.54100	0.77000	C	2.73300	-1.06300	-0.90000
C	-1.38600	-1.38000	0.70300	C	1.37400	-0.74600	0.66200	C	1.38100	-0.95000	-1.21600
C	-0.78700	-0.39100	1.48900	C	0.75900	-0.92600	-0.58200	C	0.78600	0.29000	-1.46400
C	-1.56800	0.66500	1.96300	C	1.54700	-0.92800	-1.73500	C	1.57700	1.44100	-1.42100
C	-2.91500	0.73300	1.62300	C	2.92300	-0.72800	-1.63600	C	2.92700	1.34000	-1.10900
C	-3.50800	-0.25000	0.81300	C	3.52800	-0.52700	-0.38800	C	3.51700	0.09100	-0.83800
O	-0.88700	-3.36100	-0.99600	O	0.94700	-0.08200	3.20000	O	0.87500	-3.47700	-0.57300
O	-0.36800	-3.66800	1.48200	O	0.16100	-2.33800	2.34000	O	0.33200	-2.64500	-2.92200
O	0.54800	-0.38000	1.79300	O	-0.58700	-1.09200	-0.72600	O	-0.55200	0.44700	-1.71300
H	2.99500	0.38000	1.61700	H	-2.98600	-0.45200	-1.32700	H	-2.99500	1.05300	-1.21100
H	1.76900	-3.33500	-1.47500	H	-1.60800	0.87600	3.31700	H	-1.77700	-3.65400	-0.10600
H	4.09500	-2.35600	-1.54500	H	-3.94200	1.34700	2.56200	H	-4.09600	-2.80200	0.40900
H	-3.16600	-2.11200	-0.25900	H	3.18500	-0.40000	1.76000	H	3.15900	-2.05300	-0.72500
H	-1.09200	1.43900	2.56500	H	1.05900	-1.05900	-2.70100	H	1.10500	2.40800	-1.60200
C	-10.89500	1.75200	5.19400	C	10.62800	-4.38500	-3.92300	C	10.74700	3.76000	-4.27900
C	-12.07000	1.13100	4.76300	C	11.80000	-4.36600	-3.16400	C	11.96900	3.57900	-3.57600
C	-12.03300	0.29500	3.64500	C	11.81100	-3.69600	-1.93800	C	11.96800	2.79600	-2.39700
C	-10.83300	0.08000	2.96600	C	10.66200	-3.05500	-1.47600	C	10.81400	2.21100	-1.93200
C	-9.64600	0.70000	3.38800	C	9.47700	-3.06400	-2.23100	C	9.53900	2.36800	-2.62800
C	-9.69700	1.53900	4.51300	C	9.48000	-3.74000	-3.46200	C	9.58000	3.18700	-3.83500
C	-7.15300	0.36100	3.37400	C	6.97800	-2.94300	-1.95800	C	7.08100	1.92400	-2.85000
C	-8.36900	0.47400	2.66200	C	8.25600	-2.37400	-1.74100	C	8.36000	1.76800	-2.15500
C	-8.34900	0.36400	1.26800	C	8.34700	-1.15900	-1.05400	C	8.34000	0.93900	-0.94000
C	-7.13600	0.15700	0.60400	C	7.18500	-0.52400	-0.60500	C	7.14600	0.41800	-0.48000
C	-5.93400	0.06600	1.34100	C	5.92100	-1.10800	-0.85100	C	5.92400	0.66100	-1.16300
C	-5.93300	0.15400	2.73700	C	5.80800	-2.33100	-1.52200	C	5.90600	1.39700	-2.38700
N	-4.87200	-0.15100	0.45800	N	4.92700	-0.29900	-0.30500	N	4.88600	0.01000	-0.50600
C	-6.78400	-0.01300	-0.79400	C	6.94000	0.70400	0.12700	C	6.81200	-0.43600	0.65200
C	-7.52900	-0.00200	-1.97700	C	7.77500	1.70500	0.63000	C	7.57600	-0.98900	1.68000
C	-6.88500	-0.17100	-3.20700	C	7.22100	2.80700	1.29000	C	6.95600	-1.76200	2.67100
C	-5.48200	-0.34600	-3.22400	C	5.81500	2.88300	1.43100	C	5.55900	-1.95400	2.61000
C	-4.71800	-0.36200	-2.06200	C	4.96200	1.90000	0.94200	C	4.77500	-1.41300	1.59300
C	-5.38400	-0.20100	-0.84300	C	5.53800	0.80400	0.29000	C	5.41800	-0.66200	0.60600
C	-9.65800	-0.73100	-5.74000	C	10.15400	4.59200	2.91400	C	9.79300	-3.43300	4.56100

C	-9.12600	-0.12900	-6.88300	C	9.74800	5.92700	2.85100	C	9.27300	-3.50200	5.85500
C	-7.86000	0.45700	-6.82400	C	8.51300	6.24100	2.27900	C	7.99300	-3.00300	6.10800
C	-7.13400	0.44200	-5.63300	C	7.69400	5.23000	1.77400	C	7.24200	-2.43900	5.07700
C	-7.65600	-0.16000	-4.47700	C	8.09000	3.88300	1.83200	C	7.75300	-2.36400	3.77100
C	-8.93000	-0.74800	-4.55100	C	9.33300	3.58300	2.41300	C	9.04100	-2.87100	3.53000
H	-10.91300	2.41400	6.06200	H	10.60900	-4.89900	-4.88700	H	10.73400	4.36400	-5.18800
H	-13.00800	1.29800	5.29500	H	12.69900	-4.87000	-3.52500	H	12.88900	4.03600	-3.93900
H	-12.94300	-0.20300	3.30300	H	12.71900	-3.68200	-1.33100	H	12.90000	2.65200	-1.84500
H	-10.80700	-0.59900	2.11100	H	10.67100	-2.56000	-0.50300	H	10.85900	1.62000	-1.01800
H	-8.78800	2.05000	4.84100	H	8.57600	-3.73600	-4.07500	H	8.66800	3.35600	-4.40500
H	-7.17700	0.40700	4.46500	H	6.91300	-3.91100	-2.46000	H	7.05600	2.47400	-3.78900
H	-9.27600	0.46000	0.69700	H	9.32300	-0.69500	-0.88900	H	9.26000	0.71600	-0.40500
H	-5.01500	0.04300	3.31500	H	4.83500	-2.80100	-1.68100	H	4.98300	1.52300	-2.95400
H	-8.60800	0.16900	-1.94300	H	8.85600	1.64300	0.48200	H	8.65000	-0.79300	1.73000
H	-4.98300	-0.49800	-4.18400	H	5.38700	3.73200	1.96800	H	5.07800	-2.57100	3.37200
H	-3.63700	-0.50200	-2.11000	H	3.88200	1.98000	1.07300	H	3.69800	-1.58200	1.57700
H	-10.64300	-1.20100	-5.77700	H	11.11300	4.33300	3.36800	H	10.78900	-3.82900	4.34900
H	-9.69500	-0.11800	-7.81400	H	10.38900	6.71800	3.24600	H	9.86100	-3.94300	6.66200
H	-7.43700	0.93700	-7.70900	H	8.18700	7.28100	2.21700	H	7.57900	-3.04400	7.11800
H	-6.15600	0.92800	-5.58800	H	6.74300	5.48700	1.30300	H	6.25300	-2.02500	5.29000
H	-9.34000	-1.24600	-3.66900	H	9.64500	2.53800	2.49300	H	9.44300	-2.84800	2.51400
H	-3.52200	1.57500	1.96200	H	3.54100	-0.70100	-2.53600	H	3.54000	2.24100	-1.03800

Table S23: Coordinates of the optimized minimum in the S_0, S_1, and T_1 of 36PCP at the MN15/cc-pVDZ level.

S_0				S_1				T_1			
C	12.06500	-3.11000	0.96300	C	11.70500	-3.69100	1.02400	C	-11.92900	-3.38800	-1.35800
C	12.69000	-2.24100	1.86000	C	12.28900	-3.08800	2.14000	C	-12.81500	-2.28300	-1.46900
C	11.96800	-1.17400	2.39900	C	11.60500	-2.06600	2.80300	C	-12.29600	-0.97400	-1.31800
C	10.63300	-0.97900	2.04400	C	10.35100	-1.65300	2.35500	C	-10.95900	-0.76300	-1.07400
C	9.99300	-1.84400	1.14100	C	9.75300	-2.24900	1.23200	C	-10.01600	-1.87200	-0.95800
C	10.73100	-2.91300	0.60700	C	10.45200	-3.27500	0.57400	C	-10.59000	-3.20400	-1.11300
C	7.70900	-2.73600	0.57400	C	7.49300	-2.74900	0.24600	C	-7.70100	-2.76600	-0.59600
C	8.57300	-1.63100	0.76000	C	8.41800	-1.80600	0.75100	C	-8.64900	-1.65700	-0.71200
C	8.06300	-0.34000	0.58800	C	8.05400	-0.45600	0.79300	C	-8.08600	-0.30600	-0.56700
C	6.72500	-0.16300	0.22600	C	6.79600	-0.06100	0.32800	C	-6.74800	-0.14600	-0.26300
C	5.89200	-1.28600	0.02700	C	5.89800	-1.02500	-0.18700	C	-5.89200	-1.26700	-0.09300
C	6.37400	-2.58600	0.21400	C	6.23600	-2.38300	-0.22200	C	-6.37600	-2.59400	-0.30100
N	4.60600	-0.84600	-0.30900	N	4.71800	-0.39200	-0.57500	N	-4.59900	-0.82800	0.17900
C	5.90700	1.01300	-0.00600	C	6.12200	1.22000	0.24300	C	-5.91300	1.03300	-0.07900
C	6.17800	2.38400	0.01700	C	6.49700	2.52600	0.57500	C	-6.18700	2.40100	-0.09900
C	5.16700	3.30000	-0.29400	C	5.61200	3.58500	0.34200	C	-5.16500	3.32200	0.16600
C	3.88600	2.80900	-0.63600	C	4.35100	3.30700	-0.23500	C	-3.87300	2.83600	0.46300
C	3.59000	1.45000	-0.66300	C	3.95300	2.01800	-0.57100	C	-3.57500	1.47500	0.48600
C	4.61200	0.55300	-0.33200	C	4.84800	0.97200	-0.31900	C	-4.60400	0.57700	0.19500
C	6.52300	6.70000	0.71300	C	7.09500	6.56500	2.17200	C	-6.57400	6.70900	-0.81100

C	5.94600	7.53300	-0.24700	C	6.72200	7.62500	1.34300	C	-5.96000	7.55200	0.11800
C	5.11200	6.98200	-1.22300	C	5.98300	7.36300	0.18700	C	-5.08400	7.01200	1.06200
C	4.86000	5.61000	-1.23800	C	5.62300	6.05500	-0.13700	C	-4.82600	5.64100	1.07800
C	5.43300	4.76100	-0.27700	C	5.99100	4.97900	0.68800	C	-5.43600	4.78200	0.14900
C	6.26800	5.32900	0.69900	C	6.73200	5.25700	1.84900	C	-6.31400	5.33900	-0.79600
H	12.62200	-3.94300	0.53000	H	12.23300	-4.48600	0.49200	H	-12.31800	-4.40200	-1.46800
H	13.73400	-2.39500	2.13900	H	13.27000	-3.41300	2.49200	H	-13.87500	-2.44100	-1.66500
H	12.44300	-0.49400	3.10800	H	12.04700	-1.59300	3.68300	H	-12.96800	-0.11600	-1.39700
H	10.06600	-0.16000	2.49200	H	9.81000	-0.87400	2.89600	H	-10.60400	0.26000	-0.96000
H	10.25800	-3.58100	-0.11700	H	10.01600	-3.73200	-0.31700	H	-9.95000	-4.08100	-1.03300
H	8.09800	-3.74100	0.74800	H	7.76600	-3.80700	0.25000	H	-8.05600	-3.78200	-0.76100
H	8.71500	0.52800	0.71600	H	8.76000	0.29100	1.16400	H	-8.70900	0.57400	-0.71300
H	5.72600	-3.45700	0.10300	H	5.53200	-3.13100	-0.58900	H	-5.70600	-3.45300	-0.24500
H	7.18600	2.73900	0.24400	H	7.49000	2.72300	0.98700	H	-7.20500	2.75200	-0.28700
H	3.09400	3.52600	-0.86600	H	3.65700	4.13400	-0.40100	H	-3.07400	3.55400	0.66000
H	2.59300	1.10400	-0.93800	H	2.97400	1.83000	-1.01500	H	-2.56800	1.13400	0.72900
H	7.17000	7.12100	1.48600	H	7.66600	6.75700	3.08300	H	-7.25300	7.12200	-1.56000
H	6.14500	8.60700	-0.23600	H	7.00500	8.64800	1.59600	H	-6.16200	8.62500	0.10600
H	4.66300	7.62300	-1.98500	H	5.69200	8.18200	-0.47400	H	-4.60500	7.66000	1.79900
H	4.23000	5.18400	-2.02200	H	5.07000	5.85600	-1.05800	H	-4.16200	5.22300	1.83900
H	6.70000	4.68800	1.47100	H	7.00300	4.43600	2.51700	H	-6.77600	4.69000	-1.54400
C	2.27600	-1.43900	0.03300	C	2.32800	-0.84700	-0.55500	C	-2.26200	-1.41500	-0.12400
C	1.18200	-2.25000	-0.26500	C	1.21000	-1.47100	-1.11100	C	-1.17500	-2.22900	0.18900
C	1.32900	-3.31100	-1.16300	C	1.36800	-2.29300	-2.23300	C	-1.33600	-3.29100	1.08400
C	2.54700	-3.53800	-1.80800	C	2.61600	-2.45700	-2.83000	C	-2.56500	-3.51700	1.70900
C	3.63400	-2.71900	-1.53300	C	3.73200	-1.82000	-2.29100	C	-3.64700	-2.69800	1.41800
C	3.50100	-1.67600	-0.59700	C	3.58400	-1.03100	-1.14300	C	-3.49900	-1.65300	0.48600
S	-0.00500	-4.45000	-1.34700	S	-0.04600	-3.19300	-2.80400	S	-0.00900	-4.43500	1.28300
C	-2.55100	-3.52700	-1.82200	C	-2.43300	-1.91400	-3.27000	C	2.53400	-3.52300	1.79200
C	-1.33600	-3.30600	-1.17100	C	-1.22300	-1.96900	-2.50500	C	1.32800	-3.29500	1.12600
C	-1.19100	-2.24700	-0.26900	C	-1.16200	-1.28400	-1.25700	C	1.19800	-2.23400	0.22600
C	-2.28500	-1.43500	0.02700	C	-2.23400	-0.58100	-0.76700	C	2.29900	-1.42500	-0.05400
C	-3.50800	-1.66900	-0.60900	C	-3.44900	-0.54200	-1.54300	C	3.51300	-1.66600	0.59600
C	-3.63800	-2.70600	-1.55000	C	-3.52600	-1.24000	-2.78900	C	3.62700	-2.70600	1.53600
O	-0.00200	-5.01500	-2.70500	O	0.08300	-3.44800	-4.25400	O	-0.03200	-5.00400	2.63900
O	-0.01100	-5.33900	-0.17700	O	-0.19500	-4.35600	-1.90200	O	0.01000	-5.32200	0.11100
O	-0.00500	-1.94300	0.34300	O	0.00500	-1.25000	-0.51500	O	0.02200	-1.92400	-0.40200
H	2.16100	-0.63000	0.75500	H	2.20700	-0.22500	0.33300	H	-2.13300	-0.61100	-0.84900
H	2.61200	-4.35500	-2.52800	H	2.68200	-3.08100	-3.72300	H	-2.64200	-4.33500	2.42800
H	4.58800	-2.86100	-2.04200	H	4.71700	-1.92000	-2.75000	H	-4.60800	-2.84000	1.91300
H	-2.61500	-4.34200	-2.54500	H	-2.45400	-2.42000	-4.23700	H	2.58500	-4.34100	2.51400
H	-2.17100	-0.62900	0.75300	H	-2.12300	-0.01600	0.16000	H	2.19800	-0.61800	-0.78000
H	-4.58900	-2.84400	-2.06600	H	-4.45600	-1.21100	-3.36300	H	4.57100	-2.84900	2.06300
C	-4.68800	7.02100	0.42900	C	-8.01100	6.78400	0.11200	C	4.75200	7.02400	-0.37500
C	-5.91100	7.54000	-0.00300	C	-9.39800	6.64900	0.21800	C	5.97200	7.53400	0.07600
C	-6.88700	6.67400	-0.50100	C	-9.97900	5.38200	0.12600	C	6.93700	6.65900	0.58000
C	-6.64200	5.30300	-0.56700	C	-9.17900	4.25700	-0.06600	C	6.68400	5.28800	0.63400
C	-5.41800	4.76800	-0.13400	C	-7.78100	4.37900	-0.17900	C	5.46300	4.76300	0.18100
C	-4.44500	5.64900	0.36500	C	-7.21100	5.66200	-0.08800	C	4.50100	5.65300	-0.32400

C	-3.88000	2.82000	-0.55100	C	-5.75600	3.26900	-1.16400	C	3.91000	2.82100	0.56600
C	-5.16000	3.30600	-0.19900	C	-6.93400	3.18700	-0.38900	C	5.19600	3.30200	0.23300
C	-6.17700	2.38600	0.07900	C	-7.30000	1.94400	0.19500	C	6.21200	2.37800	-0.03900
C	-5.91100	1.01500	0.01900	C	-6.49600	0.83900	-0.00000	C	5.93800	1.00900	0.01000
C	-4.61700	0.55900	-0.31300	C	-5.31900	0.95700	-0.78700	C	4.63600	0.55700	0.32400
C	-3.59100	1.46100	-0.61700	C	-4.93500	2.16600	-1.37800	C	3.61200	1.46300	0.62100
N	-4.61400	-0.84100	-0.32100	N	-4.67400	-0.25900	-0.84900	N	4.62700	-0.84200	0.32400
C	-6.73200	-0.16300	0.22700	C	-6.54400	-0.55900	0.43900	C	6.75500	-0.17300	-0.19500
C	-8.06900	-0.34400	0.59200	C	-7.40700	-1.30300	1.21800	C	8.09500	-0.35900	-0.54400
C	-8.58000	-1.63700	0.75000	C	-7.12200	-2.67600	1.45700	C	8.60000	-1.65400	-0.70300
C	-7.71800	-2.73900	0.54500	C	-5.96600	-3.25800	0.88900	C	7.73000	-2.75200	-0.51500
C	-6.38400	-2.58600	0.18100	C	-5.08700	-2.52900	0.09600	C	6.39300	-2.59400	-0.16500
C	-5.90100	-1.28400	0.00900	C	-5.39300	-1.17700	-0.11800	C	5.91500	-1.29100	0.00700
C	-12.34600	-1.21900	0.98600	C	-10.26800	-3.98800	3.10900	C	12.37100	-1.25600	-0.89400
C	-12.69200	-2.24700	1.86600	C	-9.75700	-5.01300	3.90800	C	12.72200	-2.28200	-1.77400
C	-11.69400	-3.07700	2.38000	C	-8.38400	-5.27400	3.90600	C	11.72500	-3.10400	-2.30400
C	-10.36100	-2.88000	2.01800	C	-7.52800	-4.51900	3.10800	C	10.38900	-2.90100	-1.95700
C	-9.99900	-1.85100	1.13400	C	-8.02900	-3.48000	2.30000	C	10.02200	-1.87400	-1.07100
C	-11.01300	-1.02500	0.62300	C	-9.41300	-3.22500	2.31700	C	11.03500	-1.05600	-0.54500
H	-3.92100	7.68800	0.82800	H	-7.54900	7.77000	0.19400	H	3.99300	7.69800	-0.77900
H	-6.10200	8.61400	0.04800	H	-10.02500	7.52900	0.37200	H	6.16900	8.60700	0.03500
H	-7.84300	7.07000	-0.85000	H	-11.06200	5.26900	0.19700	H	7.89100	7.04700	0.94400
H	-7.40000	4.63500	-0.98400	H	-9.64500	3.27500	-0.16500	H	7.43300	4.61400	1.05600
H	-3.49700	5.24800	0.73200	H	-6.12600	5.77400	-0.14100	H	3.55600	5.26000	-0.70500
H	-3.09900	3.53900	-0.80700	H	-5.50200	4.22000	-1.63400	H	3.12900	3.54200	0.81800
H	-7.17200	2.73800	0.36400	H	-8.19400	1.88100	0.81900	H	7.21100	2.72700	-0.30900
H	-2.60000	1.11900	-0.91800	H	-4.03200	2.21200	-1.98600	H	2.61600	1.12400	0.90700
H	-8.70600	0.52400	0.78400	H	-8.28300	-0.84300	1.68000	H	8.73900	0.50600	-0.72500
H	-8.12100	-3.74800	0.65800	H	-5.77400	-4.31900	1.05300	H	8.12900	-3.76300	-0.62700
H	-5.74400	-3.45700	0.03800	H	-4.19700	-2.97100	-0.35500	H	5.74600	-3.46200	-0.03400
H	-13.12000	-0.57000	0.57100	H	-11.34100	-3.78600	3.09600	H	13.14300	-0.61300	-0.46600
H	-13.73500	-2.40000	2.14900	H	-10.42700	-5.60800	4.53200	H	13.76700	-2.44000	-2.04600
H	-11.95200	-3.87800	3.07500	H	-7.97600	-6.06700	4.53600	H	11.98700	-3.90300	-3.00000
H	-9.58300	-3.51600	2.44700	H	-6.45400	-4.71300	3.13400	H	9.61200	-3.53100	-2.39700
H	-10.75300	-0.23700	-0.08900	H	-9.82700	-2.44500	1.67400	H	10.77100	-0.27000	0.16700

Table S24: Coordinates of the optimized minimum in the S_0, S_1, and T_1 of 18PCD at the MN15/cc-pVDZ level.

S_0				S_1				T_1			
C	-3.30200	7.19400	-2.85000	C	1.17900	-7.62700	-1.85800	C	-3.12500	7.09200	-3.03300
C	-3.46900	6.86900	-4.19800	C	1.34600	-7.47800	-3.23700	C	-3.38100	6.75200	-4.36300
C	-3.55000	5.52600	-4.57400	C	1.69400	-6.22700	-3.75200	C	-3.56300	5.41000	-4.70500
C	-3.46400	4.52000	-3.61200	C	1.87000	-5.13800	-2.89800	C	-3.49000	4.41900	-3.72700
C	-3.29900	4.83300	-2.25300	C	1.70900	-5.27400	-1.50900	C	-3.23600	4.74700	-2.38500
C	-3.21900	6.18700	-1.88800	C	1.36100	-6.53800	-1.00500	C	-3.05400	6.10000	-2.05400
C	-2.33000	3.88500	-0.13300	C	1.07800	-3.95000	0.53600	C	-2.22300	3.78300	-0.29300

C	-3.21600	3.76000	-1.22900	C	1.90700	-4.11600	-0.60000	C	-3.16500	3.69100	-1.34300
C	-4.01000	2.61300	-1.33300	C	2.91000	-3.17400	-0.85300	C	-4.02900	2.59100	-1.38300
C	-3.91800	1.61700	-0.35600	C	3.08500	-2.09700	0.02200	C	-3.94800	1.61100	-0.39000
C	-3.01300	1.76700	0.71700	C	2.23900	-1.96200	1.14600	C	-2.98400	1.72600	0.63600
C	-2.21300	2.90400	0.84600	C	1.22400	-2.88400	1.41400	C	-2.11600	2.81700	0.70100
N	-3.11600	0.64900	1.55800	N	2.61400	-0.83200	1.86900	N	-3.11800	0.63400	1.50800
C	-4.62000	0.36300	-0.14500	C	4.02400	-0.99100	0.07400	C	-4.71500	0.40800	-0.11800
C	-5.61500	-0.31600	-0.85300	C	5.08500	-0.58100	-0.73900	C	-5.78200	-0.22300	-0.76300
C	-6.10100	-1.53800	-0.37300	C	5.81800	0.56300	-0.40400	C	-6.31700	-1.40300	-0.23100
C	-5.56500	-2.06700	0.82400	C	5.46500	1.28900	0.75900	C	-5.75700	-1.94100	0.95000
C	-4.57000	-1.41300	1.54400	C	4.41400	0.90400	1.58400	C	-4.68900	-1.33600	1.60600
C	-4.10700	-0.19300	1.04700	C	3.69800	-0.24300	1.22900	C	-4.18100	-0.15400	1.06200
C	-9.21100	-2.27000	-2.42600	C	8.87000	0.49900	-2.66600	C	-9.55600	-1.98300	-2.12700
C	-9.17600	-3.66400	-2.51100	C	9.10500	1.86100	-2.87100	C	-9.61100	-3.37700	-2.17800
C	-8.13500	-4.36200	-1.89500	C	8.26300	2.79800	-2.26700	C	-8.58900	-4.12700	-1.59200
C	-7.13800	-3.67400	-1.20300	C	7.19800	2.37800	-1.47000	C	-7.52100	-3.48700	-0.96100
C	-7.16200	-2.27300	-1.10900	C	6.95100	1.01200	-1.25300	C	-7.45400	-2.08600	-0.90100
C	-8.21600	-1.58300	-1.73100	C	7.80800	0.08100	-1.86400	C	-8.49000	-1.34400	-1.49300
H	-3.24500	8.24100	-2.54300	H	0.91600	-8.60200	-1.44100	H	-2.98700	8.13900	-2.75200
H	-3.53400	7.65600	-4.95100	H	1.20600	-8.33000	-3.90500	H	-3.43600	7.52800	-5.12900
H	-3.67000	5.25900	-5.62600	H	1.81900	-6.09400	-4.82900	H	-3.75400	5.13100	-5.74400
H	-3.49800	3.47100	-3.91700	H	2.11300	-4.15600	-3.31200	H	-3.60300	3.36900	-4.00700
H	-3.11500	6.45100	-0.83300	H	1.25700	-6.67100	0.07400	H	-2.88000	6.37700	-1.01200
H	-1.69000	4.76700	-0.07400	H	0.27200	-4.66800	0.70500	H	-1.53000	4.62700	-0.28300
H	-4.72300	2.51200	-2.15500	H	3.57600	-3.29900	-1.71000	H	-4.78700	2.51600	-2.16700
H	-1.49200	3.00100	1.65700	H	0.54700	-2.73800	2.25600	H	-1.35100	2.88600	1.47400
H	-5.99700	0.09300	-1.79200	H	5.32600	-1.13900	-1.64800	H	-6.18200	0.18900	-1.69300
H	-5.96200	-3.01100	1.20400	H	6.05600	2.16700	1.02800	H	-6.19000	-2.85200	1.36900
H	-4.16500	-1.84000	2.46300	H	4.15300	1.47000	2.47900	H	-4.26000	-1.77100	2.51100
H	-10.02400	-1.71300	-2.89600	H	9.52600	-0.24400	-3.12400	H	-10.35400	-1.38600	-2.57500
H	-9.95500	-4.20200	-3.05300	H	9.93800	2.18900	-3.49500	H	-10.44600	-3.87800	-2.67300
H	-8.09200	-5.45200	-1.96300	H	8.43100	3.86600	-2.42500	H	-8.61600	-5.21800	-1.63400
H	-6.31200	-4.22600	-0.74900	H	6.52900	3.11700	-1.02400	H	-6.71100	-4.08000	-0.53000
H	-8.26500	-0.49500	-1.64500	H	7.65000	-0.98500	-1.68400	H	-8.46800	-0.25400	-1.43300
C	-1.57500	0.32200	5.49900	C	1.24800	0.25600	5.74500	C	-1.56500	0.38300	5.48900
C	-0.72000	0.21500	4.40200	C	0.41800	0.51100	4.62900	C	-0.67600	0.18500	4.36400
C	-1.24300	0.34500	3.11000	C	0.90000	0.11300	3.34300	C	-1.23000	0.31100	3.02600
C	-2.59900	0.55200	2.87100	C	2.15400	-0.48100	3.17300	C	-2.57000	0.53200	2.80700
C	-3.44900	0.65100	3.98200	C	2.95200	-0.71700	4.28700	C	-3.42700	0.67600	3.93500
C	-2.93900	0.53600	5.27700	C	2.48500	-0.34200	5.57200	C	-2.90100	0.60700	5.25900
S	0.00400	0.02800	1.86200	S	-0.18400	0.60100	2.04900	S	-0.00900	-0.07300	1.78700
C	2.60900	-0.45700	2.88600	C	-2.60800	1.72300	2.92700	C	2.58200	-0.51400	2.83400
C	1.25300	-0.24400	3.11900	C	-1.32700	1.24500	3.20600	C	1.23300	-0.33800	3.04000
C	0.73100	-0.06900	4.40600	C	-0.86200	1.14900	4.56100	C	0.67300	-0.16800	4.37200
C	1.58700	-0.13700	5.50600	C	-1.71500	1.63300	5.57600	C	1.56200	-0.30400	5.50900
C	2.95100	-0.35700	5.29100	C	-2.96700	2.14300	5.27400	C	2.90500	-0.49000	5.29100
C	3.46000	-0.51600	4.00000	C	-3.44600	2.17900	3.92800	C	3.43900	-0.58300	3.97200
O	-0.35100	-1.20900	1.15600	O	0.43400	1.66400	1.20900	O	-0.41100	-1.31900	1.11700
O	0.35700	1.23900	1.11100	O	-0.80300	-0.50900	1.27900	O	0.37500	1.09000	0.97500

H	-1.19000	0.22000	6.51500	H	0.91400	0.54500	6.74400	H	-1.17400	0.32800	6.50600
H	-4.51400	0.81600	3.80900	H	3.92600	-1.18700	4.15200	H	-4.48800	0.85800	3.76100
H	-3.61700	0.61700	6.12800	H	3.11700	-0.53100	6.44200	H	-3.58200	0.74100	6.10000
H	1.20200	-0.00000	6.51800	H	-1.38700	1.59000	6.61800	H	1.16300	-0.22700	6.52100
H	3.63000	-0.40700	6.14300	H	-3.61000	2.51100	6.07500	H	3.58600	-0.57400	6.13900
H	4.52500	-0.68600	3.83200	H	-4.44000	2.55400	3.68500	H	4.50500	-0.73600	3.80800
C	8.13600	4.29400	-2.05300	C	-7.39200	-4.02400	-1.65500	C	8.47700	4.19600	-1.67100
C	9.17700	3.57600	-2.64500	C	-8.18100	-3.51000	-2.68700	C	9.51000	3.46200	-2.25800
C	9.21500	2.18600	-2.51200	C	-8.12700	-2.14600	-2.98600	C	9.48700	2.06700	-2.18800
C	8.22200	1.52200	-1.79200	C	-7.28900	-1.30300	-2.25800	C	8.44200	1.41400	-1.53500
C	7.16800	2.23200	-1.19300	C	-6.49400	-1.80600	-1.21400	C	7.39500	2.13900	-0.94200
C	7.14200	3.62900	-1.33600	C	-6.55800	-3.18000	-0.92300	C	7.43000	3.54100	-1.02200
C	5.57500	2.09100	0.74900	C	-5.40500	-1.11800	0.94100	C	5.72100	1.98100	0.92800
C	6.10900	1.52100	-0.43000	C	-5.60600	-0.91000	-0.43800	C	6.28100	1.44000	-0.25200
C	5.62300	0.28300	-0.86800	C	-4.93900	0.15900	-1.08300	C	5.76600	0.24200	-0.76300
C	4.63000	-0.37200	-0.13500	C	-4.10200	0.98000	-0.34500	C	4.72000	-0.40400	-0.09900
C	4.11900	0.22400	1.03800	C	-3.93100	0.74700	1.03800	C	4.18700	0.16000	1.08100
C	4.58200	1.46100	1.49300	C	-4.57000	-0.29900	1.70100	C	4.67400	1.36100	1.60400
N	3.12700	-0.59900	1.57800	N	-3.05200	1.69200	1.55500	N	3.14700	-0.64600	1.54800
C	3.93100	-1.63500	-0.30000	C	-3.24500	2.11900	-0.67000	C	3.97800	-1.62700	-0.34700
C	4.01700	-2.66200	-1.24400	C	-2.95400	2.80800	-1.82500	C	4.06800	-2.61400	-1.33300
C	3.21300	-3.79800	-1.10800	C	-2.01100	3.87700	-1.78500	C	3.22000	-3.72500	-1.27800
C	2.32500	-3.88200	-0.01000	C	-1.39400	4.22100	-0.55400	C	2.29000	-3.82600	-0.21800
C	2.21600	-2.87200	0.93900	C	-1.67300	3.55000	0.62000	C	2.17700	-2.85500	0.77100
C	3.02200	-1.74500	0.77500	C	-2.61100	2.49700	0.55400	C	3.02500	-1.74900	0.68700
C	4.58100	-6.32100	-3.59500	C	-2.29300	5.44100	-5.21800	C	4.59300	-6.18200	-3.82900
C	3.42000	-6.99100	-3.98900	C	-1.01400	5.98200	-5.38100	C	3.42500	-6.79300	-4.29200
C	2.19200	-6.61700	-3.43900	C	-0.06200	5.83300	-4.36800	C	2.19100	-6.40100	-3.76900
C	2.12500	-5.58300	-2.50600	C	-0.38600	5.15600	-3.19600	C	2.12500	-5.40700	-2.79200
C	3.28400	-4.90200	-2.10000	C	-1.67000	4.60000	-3.01900	C	3.29100	-4.78600	-2.31600
C	4.51300	-5.28900	-2.65900	C	-2.61600	4.75000	-4.05400	C	4.52500	-5.19100	-2.85000
H	8.09100	5.38000	-2.15900	H	-7.41900	-5.09000	-1.42400	H	8.47900	5.28600	-1.72900
H	9.95500	4.09600	-3.20800	H	-8.83400	-4.17100	-3.26000	H	10.32900	3.97400	-2.76800
H	10.02800	1.61400	-2.96400	H	-8.74600	-1.73500	-3.78500	H	10.29400	1.48300	-2.63600
H	8.27300	0.43800	-1.66800	H	-7.27200	-0.23300	-2.48000	H	8.44500	0.32300	-1.46000
H	6.31400	4.19500	-0.90100	H	-5.92100	-3.59400	-0.13800	H	6.61100	4.12200	-0.59100
H	5.97100	3.04800	1.09600	H	-5.93600	-1.93400	1.43300	H	6.13700	2.90700	1.33000
H	6.00400	-0.15700	-1.79300	H	-5.05800	0.30500	-2.15800	H	6.16400	-0.17200	-1.69300
H	4.17800	1.91800	2.39800	H	-4.41400	-0.46200	2.76700	H	4.24500	1.80000	2.50600
H	4.69500	-2.56900	-2.09700	H	-3.40100	2.51200	-2.77600	H	4.78400	-2.50900	-2.15200
H	1.71600	-4.78100	0.10400	H	-0.68500	5.04800	-0.53400	H	1.64700	-4.70700	-0.16600
H	1.50800	-2.94700	1.76400	H	-1.17700	3.79000	1.55800	H	1.43600	-2.94400	1.56400
H	5.54800	-6.61100	-4.01200	H	-3.04300	5.56200	-6.00100	H	5.56400	-6.48600	-4.22500
H	3.47200	-7.80000	-4.72100	H	-0.75900	6.51700	-6.29700	H	3.47700	-7.57000	-5.05800
H	1.27600	-7.12700	-3.74700	H	0.94200	6.23900	-4.49600	H	1.27100	-6.86500	-4.13100
H	1.15700	-5.27600	-2.10200	H	0.37400	5.01600	-2.42600	H	1.15400	-5.08600	-2.40900
H	5.42800	-4.78900	-2.33500	H	-3.62500	4.35400	-3.92600	H	5.44500	-4.73700	-2.47200

Table S25: Coordinates of the optimized minimum in the S_0, S_1, and T_1 of 28PCD at the MN15/cc-pVDZ level.

S_0				S_1				T_1			
C	-5.67800	-5.22300	4.92200	C	-5.29200	-5.16900	4.97000	C	-6.18200	-4.74100	5.26700
C	-7.05500	-5.38800	5.08400	C	-6.58000	-4.97100	5.47800	C	-7.57300	-4.80900	5.37600
C	-7.93000	-4.76800	4.18900	C	-7.47300	-4.13500	4.80000	C	-8.36700	-4.21700	4.39200
C	-7.43200	-3.99200	3.14200	C	-7.08000	-3.49900	3.62700	C	-7.77600	-3.56500	3.30900
C	-6.04900	-3.81600	2.96900	C	-5.78900	-3.69600	3.09600	C	-6.37800	-3.48800	3.18800
C	-5.18000	-4.44500	3.87600	C	-4.90200	-4.54600	3.78900	C	-5.59200	-4.08700	4.18600
C	-4.34400	-3.37200	1.17300	C	-4.41600	-3.63500	0.99700	C	-4.57300	-3.30500	1.44600
C	-5.51900	-2.98400	1.85800	C	-5.37400	-3.03000	1.85300	C	-5.75000	-2.79000	2.03700
C	-6.17100	-1.80700	1.47500	C	-5.91900	-1.75800	1.51200	C	-6.31000	-1.61600	1.52200
C	-5.65400	-1.03900	0.42700	C	-5.50200	-1.12900	0.36000	C	-5.70100	-0.97700	0.43700
C	-4.46900	-1.44600	-0.22300	C	-4.54300	-1.77200	-0.48400	C	-4.52000	-1.51200	-0.12100
C	-3.80600	-2.62100	0.13400	C	-3.98600	-3.02900	-0.16700	C	-3.94400	-2.68500	0.37200
N	-4.15900	-0.51900	-1.22900	N	-4.29200	-0.98200	-1.55900	N	-4.11500	-0.70200	-1.19000
C	-6.09300	0.18300	-0.22400	C	-5.80100	0.15400	-0.27400	C	-6.03500	0.20600	-0.33600
C	-7.17800	1.04300	-0.02800	C	-6.61200	1.22800	0.05200	C	-7.06600	1.14600	-0.26400
C	-7.33400	2.16400	-0.85100	C	-6.63100	2.36500	-0.79200	C	-7.11300	2.19900	-1.18600
C	-6.38200	2.40800	-1.86700	C	-5.82400	2.38000	-1.94700	C	-6.10800	2.29100	-2.17600
C	-5.29100	1.57100	-2.07900	C	-5.00600	1.30600	-2.29600	C	-5.06800	1.37100	-2.26500
C	-5.15900	0.45700	-1.24700	C	-5.01300	0.20100	-1.44600	C	-5.04500	0.32900	-1.33500
C	-10.83000	3.46800	-0.14100	C	-9.54200	4.44100	0.46800	C	-10.53500	3.78500	-0.73600
C	-10.65800	4.84500	-0.29600	C	-9.09800	5.73800	0.20100	C	-10.26400	5.12900	-1.00100
C	-9.39800	5.34500	-0.63300	C	-7.84700	5.93200	-0.39000	C	-8.96200	5.51500	-1.32800
C	-8.32200	4.47700	-0.81200	C	-7.04700	4.83800	-0.71500	C	-7.94100	4.56500	-1.38800
C	-8.48100	3.09000	-0.66100	C	-7.48100	3.52800	-0.45100	C	-8.20100	3.21000	-1.12500
C	-9.75300	2.60000	-0.32300	C	-8.74000	3.34700	0.14800	C	-9.51500	2.83600	-0.80000
H	-4.98400	-5.69600	5.62100	H	-4.58600	-5.80800	5.50200	H	-5.55000	-5.19200	6.03600
H	-7.44500	-5.99600	5.90200	H	-6.88600	-5.46400	6.40200	H	-8.03500	-5.32000	6.22300
H	-9.00900	-4.89600	4.30000	H	-8.48200	-3.98400	5.18700	H	-9.45600	-4.27000	4.46100
H	-8.12100	-3.53300	2.43000	H	-7.79400	-2.87000	3.09200	H	-8.40300	-3.12900	2.52800
H	-4.10300	-4.29700	3.77400	H	-3.88400	-4.68200	3.41800	H	-4.50300	-4.01300	4.12400
H	-3.85300	-4.30400	1.46100	H	-4.01700	-4.61400	1.26100	H	-4.15100	-4.23300	1.83900
H	-7.07000	-1.48000	2.00400	H	-6.62400	-1.27000	2.18600	H	-7.20700	-1.18900	1.97700
H	-2.88600	-2.92600	-0.36500	H	-3.22800	-3.47300	-0.80800	H	-3.02200	-3.08300	-0.05100
H	-7.88800	0.85400	0.78100	H	-7.20400	1.22300	0.97000	H	-7.81800	1.07700	0.52600
H	-6.52100	3.27300	-2.51900	H	-5.85800	3.25300	-2.60100	H	-6.16300	3.10200	-2.90500
H	-4.56300	1.77800	-2.86500	H	-4.38100	1.31900	-3.18800	H	-4.29600	1.46100	-3.03100
H	-11.81200	3.06400	0.11400	H	-10.52200	4.28000	0.92100	H	-11.55100	3.47100	-0.48800
H	-11.50100	5.52400	-0.15500	H	-9.72400	6.59500	0.45400	H	-11.06200	5.87200	-0.95300
H	-9.25000	6.42100	-0.74800	H	-7.48700	6.94300	-0.59200	H	-8.73500	6.56400	-1.52800
H	-7.33300	4.87900	-1.04700	H	-6.05700	5.00000	-1.14900	H	-6.92000	4.87900	-1.61500
H	-9.90200	1.52200	-0.22800	H	-9.10700	2.33400	0.33500	H	-9.74100	1.78300	-0.61800
C	-1.38800	-1.16000	-4.37900	C	-1.46000	-1.69100	-4.63000	C	-1.29900	-1.76700	-4.22500
C	-0.93100	-0.85100	-3.09700	C	-1.00300	-1.33300	-3.34400	C	-0.82200	-1.33600	-2.93900
C	-1.86200	-0.65100	-2.06900	C	-1.99200	-1.16000	-2.31600	C	-1.80500	-1.01100	-1.92600
C	-3.23500	-0.73100	-2.28000	C	-3.35000	-1.26600	-2.61500	C	-3.16300	-1.04800	-2.18100
C	-3.68100	-1.03200	-3.57500	C	-3.79200	-1.60300	-3.88100	C	-3.59800	-1.44800	-3.46300

C	-2.76500	-1.24200	-4.60800	C	-2.81300	-1.83300	-4.89600	C	-2.65400	-1.80700	-4.46600
S	-1.05700	-0.13200	-0.55500	S	-1.29700	-0.67300	-0.78700	S	-1.03400	-0.43400	-0.42900
C	1.75800	-0.01100	-0.67200	C	1.48200	-0.31800	-0.79500	C	1.75300	-0.24000	-0.55900
C	0.55600	-0.24800	-1.31800	C	0.31600	-0.64900	-1.48400	C	0.57600	-0.57500	-1.16400
C	0.46400	-0.64900	-2.65300	C	0.31200	-1.04400	-2.85700	C	0.49300	-1.10600	-2.52200
C	1.64600	-0.80000	-3.38400	C	1.55900	-1.07400	-3.52400	C	1.73800	-1.27600	-3.23700
C	2.86900	-0.53900	-2.76800	C	2.71700	-0.72300	-2.85600	C	2.91900	-0.95100	-2.63300
C	2.93700	-0.15000	-1.41900	C	2.68900	-0.34500	-1.48500	C	2.96300	-0.42300	-1.28300
O	-1.39700	1.26600	-0.26900	O	-1.81300	0.65200	-0.36000	O	-1.37800	0.97200	-0.17100
O	-1.16100	-1.14700	0.50200	O	-1.42600	-1.73900	0.24600	O	-1.16900	-1.41100	0.66500
H	-0.68200	-1.31800	-5.19700	H	-0.73400	-1.84500	-5.43300	H	-0.58600	-2.03500	-5.00600
H	-4.75600	-1.09800	-3.75300	H	-4.85900	-1.69100	-4.08600	H	-4.66900	-1.48600	-3.66100
H	-3.13200	-1.47700	-5.60800	H	-3.14200	-2.11100	-5.89700	H	-3.02100	-2.12200	-5.44400
H	1.61900	-1.10500	-4.43200	H	1.60500	-1.36200	-4.57700	H	1.72800	-1.63800	-4.26600
H	3.79900	-0.62200	-3.33400	H	3.67800	-0.72400	-3.37500	H	3.85800	-1.03200	-3.18200
C	6.61100	6.83200	2.52800	C	5.93500	7.14000	1.87000	C	6.15300	6.88500	2.48200
C	7.48800	6.56400	3.58100	C	6.76600	7.02200	2.98700	C	7.07600	6.69900	3.51400
C	7.78400	5.23900	3.90700	C	7.08900	5.75100	3.46700	C	7.48600	5.40600	3.84500
C	7.20800	4.19200	3.18700	C	6.58600	4.61200	2.83900	C	6.97800	4.30900	3.14900
C	6.32600	4.44800	2.12400	C	5.75300	4.71600	1.71200	C	6.05100	4.48200	2.10900
C	6.03700	5.78500	1.80700	C	5.43700	6.00000	1.24000	C	5.64600	5.78800	1.78600
C	4.38300	3.41800	0.90000	C	3.91200	3.48900	0.51000	C	4.17000	3.29000	0.93800
C	5.72000	3.33200	1.35400	C	5.22500	3.50100	1.03900	C	5.51500	3.31200	1.36800
C	6.46100	2.18300	1.05800	C	6.01500	2.35400	0.91400	C	6.33800	2.21700	1.07000
C	5.87200	1.14400	0.33100	C	5.50100	1.22100	0.27600	C	5.81400	1.12600	0.37600
C	4.53000	1.25200	-0.09600	C	4.18200	1.23700	-0.23700	C	4.45800	1.12000	-0.01700
C	3.77400	2.39700	0.17600	C	3.37500	2.37600	-0.12600	C	3.62600	2.21500	0.24100
N	4.18700	0.10200	-0.81800	N	3.90200	0.01000	-0.83000	N	4.18800	-0.06600	-0.72000
C	6.36500	-0.13300	-0.15100	C	6.04600	-0.08700	-0.03100	C	6.40000	-0.11200	-0.11100
C	7.59200	-0.79200	-0.03000	C	7.27900	-0.70000	0.21200	C	7.67700	-0.66400	-0.01400
C	7.76100	-2.05900	-0.59900	C	7.50100	-2.01300	-0.21500	C	7.93300	-1.92600	-0.56900
C	6.67300	-2.65200	-1.28000	C	6.46000	-2.70100	-0.88300	C	6.87600	-2.62400	-1.19500
C	5.44300	-2.01600	-1.41400	C	5.22400	-2.11700	-1.13600	C	5.59200	-2.09500	-1.29700
C	5.30200	-0.74300	-0.85400	C	5.02800	-0.79800	-0.71000	C	5.37100	-0.81700	-0.77300
C	11.03600	-3.37200	0.80600	C	10.74300	-3.08400	1.45100	C	11.35500	-2.93000	0.73200
C	11.51800	-4.13600	-0.26000	C	11.28800	-3.94200	0.49300	C	11.85700	-3.67700	-0.33600
C	10.77000	-4.22100	-1.43600	C	10.59300	-4.17000	-0.69700	C	11.07400	-3.85200	-1.48000
C	9.55300	-3.54800	-1.54600	C	9.36700	-3.54500	-0.92600	C	9.80200	-3.28500	-1.55400
C	9.05600	-2.77800	-0.48200	C	8.80500	-2.68100	0.02900	C	9.28600	-2.53100	-0.48700
C	9.81700	-2.70200	0.69600	C	9.51500	-2.46400	1.22200	C	10.08300	-2.36300	0.65700
H	6.37800	7.86500	2.25900	H	5.68200	8.12800	1.47900	H	5.83200	7.89200	2.21000
H	7.93800	7.38300	4.14500	H	7.15700	7.91400	3.48000	H	7.47200	7.55800	4.05900
H	8.46100	5.01700	4.73500	H	7.73000	5.64500	4.34500	H	8.19900	5.24800	4.65600
H	7.42100	3.15800	3.46700	H	6.82000	3.62200	3.23800	H	7.28200	3.29800	3.43300
H	5.37400	6.00500	0.96700	H	4.81100	6.10200	0.34900	H	4.94600	5.94400	0.96300
H	3.80100	4.30800	1.14700	H	3.29000	4.37900	0.63000	H	3.52700	4.13900	1.17800
H	7.50700	2.11100	1.36600	H	7.04300	2.35300	1.28800	H	7.39500	2.23400	1.34900
H	2.74200	2.49700	-0.16100	H	2.35700	2.38500	-0.52000	H	2.58700	2.24100	-0.08800
H	8.42200	-0.31100	0.49200	H	8.07700	-0.14500	0.71300	H	8.47900	-0.10800	0.47700

H	6.79500	-3.65800	-1.68800	H	6.62600	-3.73800	-1.18400	H	7.06400	-3.62900	-1.57900
H	4.61300	-2.51200	-1.92100	H	4.42700	-2.66900	-1.63600	H	4.78600	-2.67700	-1.74500
H	11.60700	-3.30600	1.73400	H	11.27200	-2.90500	2.39000	H	11.95400	-2.79400	1.63500
H	12.47000	-4.66100	-0.17400	H	12.24800	-4.43000	0.67200	H	12.85200	-4.12100	-0.27700
H	11.14100	-4.80800	-2.27900	H	11.01200	-4.83100	-1.45700	H	11.46000	-4.42700	-2.32400
H	8.98800	-3.59800	-2.48000	H	8.84400	-3.70600	-1.87200	H	9.20800	-3.40400	-2.46300
H	9.43300	-2.13200	1.54500	H	9.08100	-1.81800	1.99000	H	9.68700	-1.80500	1.50900
H	1.79300	0.26400	0.38400	H	1.45400	-0.04000	0.26100	H	1.77400	0.12500	0.47000

Table S26: Coordinates of the optimized minimum in the S_0, S_1, and T_1 of 38PCD at the MN15/cc-pVDZ level.

S_0				S_1				T_1			
C	-7.83700	-5.28200	3.80000	C	-7.61500	5.25200	-3.80100	C	-8.11300	-5.09800	3.85000
C	-9.15400	-5.45100	3.36400	C	-9.00400	5.11000	-3.72900	C	-9.44600	-5.18100	3.44300
C	-9.60600	-4.73600	2.25300	C	-9.56400	4.21800	-2.80800	C	-9.87200	-4.44800	2.33300
C	-8.74900	-3.86200	1.58400	C	-8.74100	3.47000	-1.97200	C	-8.97200	-3.64300	1.63500
C	-7.42400	-3.68100	2.01300	C	-7.33900	3.60800	-2.02700	C	-7.62800	-3.54900	2.03400
C	-6.98200	-4.40500	3.13200	C	-6.79000	4.51500	-2.95600	C	-7.21400	-4.29000	3.15300
C	-5.14900	-3.07200	1.12600	C	-5.21500	3.33600	-0.71800	C	-5.33600	-3.10700	1.09700
C	-6.51400	-2.74500	1.30100	C	-6.46900	2.82200	-1.13900	C	-6.67100	-2.68700	1.29400
C	-6.99200	-1.53200	0.79600	C	-6.87700	1.52500	-0.71100	C	-7.07400	-1.44900	0.78100
C	-6.11800	-0.66800	0.12700	C	-6.04600	0.78700	0.10100	C	-6.15500	-0.65300	0.09200
C	-4.75900	-1.02100	-0.01900	C	-4.79500	1.34100	0.51600	C	-4.82500	-1.09600	-0.07200
C	-4.25900	-2.22800	0.47200	C	-4.36500	2.61900	0.10100	C	-4.40100	-2.33300	0.41800
N	-4.09900	-0.00600	-0.72500	N	-4.15600	0.45100	1.32000	N	-4.11500	-0.13600	-0.80700
C	-6.29300	0.61700	-0.52700	C	-6.11100	-0.54800	0.69100	C	-6.25800	0.63200	-0.57600
C	-7.39000	1.46300	-0.71400	C	-7.03600	-1.57900	0.65100	C	-7.30100	1.54500	-0.75500
C	-7.23600	2.65400	-1.43200	C	-6.76800	-2.77900	1.35200	C	-7.08100	2.71900	-1.48600
C	-5.96200	2.98300	-1.95200	C	-5.56000	-2.90600	2.06800	C	-5.79700	2.96200	-2.02500
C	-4.85300	2.16200	-1.77300	C	-4.61600	-1.88100	2.11700	C	-4.74000	2.07300	-1.85400
C	-5.03500	0.97700	-1.05700	C	-4.91500	-0.70900	1.42500	C	-4.98800	0.90400	-1.13000
C	-10.43300	4.61600	-0.83800	C	-9.42700	-5.20000	0.15100	C	-10.14000	4.88000	-0.85800
C	-10.58600	5.28700	-2.05300	C	-9.61500	-5.99500	1.28300	C	-10.26700	5.55500	-2.07400
C	-9.64300	5.09700	-3.06500	C	-8.87100	-5.74000	2.43900	C	-9.35100	5.30400	-3.09800
C	-8.55800	4.24400	-2.86400	C	-7.94600	-4.69800	2.46000	C	-8.31800	4.38600	-2.90700
C	-8.39000	3.56500	-1.64600	C	-7.74900	-3.88800	1.32800	C	-8.17800	3.70000	-1.68900
C	-9.34500	3.76600	-0.63600	C	-8.50600	-4.15400	0.17300	C	-9.10500	3.96400	-0.66700
H	-7.47500	-5.82900	4.67300	H	-7.17200	5.93600	-4.52700	H	-7.77100	-5.65900	4.72200
H	-9.82300	-6.13600	3.88800	H	-9.64900	5.69100	-4.38900	H	-10.15000	-5.81200	3.98900
H	-10.63000	-4.86600	1.89800	H	-10.64700	4.11000	-2.74000	H	-10.91000	-4.51200	2.00000
H	-9.10100	-3.32700	0.69900	H	-9.19000	2.79700	-1.23900	H	-9.30500	-3.09500	0.75000
H	-5.96300	-4.25700	3.49600	H	-5.70700	4.61000	-3.04300	H	-6.17900	-4.20800	3.49400
H	-4.78700	-4.03100	1.50400	H	-4.91900	4.33400	-1.04200	H	-5.03200	-4.08500	1.47600
H	-8.03800	-1.25100	0.93800	H	-7.82300	1.10900	-1.06300	H	-8.09800	-1.09700	0.93600
H	-3.20500	-2.48800	0.36900	H	-3.38800	2.99600	0.39800	H	-3.37200	-2.67000	0.29900
H	-8.37200	1.18200	-0.32500	H	-7.97500	-1.46600	0.10500	H	-8.29200	1.33200	-0.34700

H	-5.84100	3.93000	-2.48300	H	-5.34400	-3.85000	2.57100	H	-5.62200	3.89600	-2.56400
H	-3.87200	2.44500	-2.15800	H	-3.67200	-1.98900	2.65200	H	-3.74800	2.29200	-2.25200
H	-11.15900	4.76400	-0.03500	H	-9.99500	-5.40000	-0.76000	H	-10.84400	5.07500	-0.04700
H	-11.43600	5.95400	-2.21000	H	-10.33700	-6.81300	1.26600	H	-11.07600	6.27300	-2.22300
H	-9.75700	5.61000	-4.02300	H	-9.01800	-6.35100	3.33100	H	-9.44500	5.82000	-4.05600
H	-7.83900	4.08000	-3.67100	H	-7.38800	-4.48800	3.37500	H	-7.62200	4.17500	-3.72200
H	-9.21600	3.26800	0.32800	H	-8.34300	-3.55700	-0.72700	H	-8.99400	3.46300	0.29700
C	-0.27700	-0.34700	-2.54200	C	-0.27500	0.81600	2.97900	C	-0.29000	-0.62300	-2.66200
C	-0.38400	-0.14500	-1.16600	C	-0.41100	0.59800	1.59100	C	-0.36500	-0.46100	-1.23000
C	-1.65400	-0.04700	-0.58300	C	-1.74500	0.54700	1.05800	C	-1.68300	-0.35100	-0.62200
C	-2.82500	-0.12300	-1.33100	C	-2.84900	0.62400	1.90500	C	-2.83400	-0.29100	-1.38300
C	-2.70400	-0.31600	-2.71600	C	-2.70900	0.82200	3.26700	C	-2.71900	-0.40200	-2.79100
C	-1.44500	-0.42500	-3.30800	C	-1.38700	0.93700	3.79700	C	-1.44300	-0.58100	-3.40600
S	-1.54400	0.33100	1.16400	S	-1.76800	0.24000	-0.66400	S	-1.55800	-0.15500	1.14200
C	1.09000	0.54800	2.17500	C	0.74600	-0.19100	-1.83500	C	1.04900	0.34200	2.07000
C	0.24000	0.32300	1.10100	C	-0.01700	0.09800	-0.70500	C	0.20200	0.00900	1.03300
C	0.70800	0.03700	-0.18500	C	0.56600	0.34600	0.57600	C	0.67900	-0.27500	-0.31900
C	2.08300	-0.01500	-0.41100	C	1.97300	0.27200	0.67400	C	2.09300	-0.22800	-0.54900
C	2.95900	0.20000	0.66300	C	2.73100	-0.00800	-0.45300	C	2.93700	0.08300	0.49200
C	2.46500	0.47700	1.94900	C	2.12900	-0.23900	-1.72000	C	2.42700	0.37300	1.82000
O	-2.05900	1.68200	1.41500	O	-2.51000	-1.00800	-0.97800	O	-2.20200	1.08500	1.59600
O	-2.00200	-0.79400	1.99100	O	-2.22900	1.43000	-1.43300	O	-1.86400	-1.41600	1.84000
H	0.70200	-0.42400	-3.01800	H	0.72500	0.87500	3.41700	H	0.68200	-0.74500	-3.14200
H	-3.61600	-0.37700	-3.31300	H	-3.58900	0.88900	3.90700	H	-3.63000	-0.37500	-3.38900
H	-1.37200	-0.57700	-4.38600	H	-1.25700	1.10500	4.86700	H	-1.39400	-0.68500	-4.49100
H	2.49100	-0.20700	-1.40500	H	2.47700	0.42300	1.63200	H	2.50200	-0.40900	-1.54400
C	9.85400	5.16900	3.05300	C	8.85300	-5.76700	-3.09200	C	9.40900	5.34800	3.29300
C	10.90500	5.25600	2.13800	C	9.98900	-5.90000	-2.29000	C	10.51800	5.48800	2.45600
C	10.84900	4.51400	0.95500	C	10.13300	-5.08600	-1.16500	C	10.58000	4.75700	1.26700
C	9.75000	3.69600	0.69100	C	9.15000	-4.15000	-0.84300	C	9.54100	3.89500	0.91800
C	8.68700	3.59800	1.60300	C	8.00400	-4.00200	-1.64200	C	8.42000	3.74300	1.75100
C	8.75700	4.34800	2.78900	C	7.87300	-4.82700	-2.77100	C	8.37200	4.48300	2.94400
C	6.21600	3.13200	1.68300	C	5.59100	-3.30100	-1.50800	C	5.97300	3.17100	1.65400
C	7.52000	2.72300	1.32200	C	6.95900	-3.00000	-1.30600	C	7.31600	2.81900	1.38500
C	7.68900	1.48700	0.68800	C	7.30800	-1.74900	-0.78800	C	7.58200	1.59500	0.75900
C	6.57800	0.67800	0.43500	C	6.30900	-0.81600	-0.49200	C	6.52900	0.73800	0.43400
C	5.28900	1.10400	0.82500	C	4.95000	-1.13900	-0.72000	C	5.20000	1.10300	0.74200
C	5.09400	2.34500	1.44200	C	4.57800	-2.39300	-1.22000	C	4.90700	2.33700	1.33400
N	4.35400	0.13400	0.44700	N	4.15100	-0.06300	-0.34800	N	4.33400	0.09200	0.31300
C	6.41700	-0.61500	-0.20500	C	6.32700	0.53000	0.04600	C	6.46600	-0.55700	-0.22100
C	7.31900	-1.52400	-0.76400	C	7.35300	1.39100	0.44900	C	7.44300	-1.41900	-0.72400
C	6.85300	-2.73500	-1.28900	C	7.04300	2.67500	0.91100	C	7.06800	-2.64800	-1.27900
C	5.46800	-3.01500	-1.23200	C	5.68700	3.07600	0.95700	C	5.69700	-2.99200	-1.30800
C	4.55000	-2.12500	-0.68200	C	4.64800	2.23800	0.56700	C	4.70600	-2.14900	-0.81700
C	5.03600	-0.91400	-0.17900	C	4.97900	0.95500	0.11500	C	5.10100	-0.92000	-0.28000
C	9.78100	-4.20700	-3.20700	C	10.25900	4.00800	2.43300	C	10.17600	-3.96900	-3.01300
C	9.59000	-5.57600	-3.01200	C	10.16300	5.37300	2.15300	C	10.03700	-5.34800	-2.83600
C	8.50200	-6.01700	-2.25500	C	9.04600	5.85500	1.46800	C	8.92600	-5.84300	-2.15100
C	7.61500	-5.09600	-1.69700	C	8.03600	4.98100	1.06600	C	7.96300	-4.96900	-1.64700

C	7.79500	-3.71600	-1.88700	C	8.11700	3.60600	1.34200	C	8.09000	-3.58100	-1.81900
C	8.89100	-3.28700	-2.65200	C	9.24600	3.13600	2.03400	C	9.21200	-3.09600	-2.51100
H	9.89100	5.73800	3.98500	H	8.73300	-6.39100	-3.97900	H	9.35400	5.90800	4.22800
H	11.76300	5.89700	2.34400	H	10.75800	-6.63400	-2.54100	H	11.33100	6.16400	2.72800
H	11.66000	4.58100	0.22800	H	11.01300	-5.18700	-0.52500	H	11.43800	4.86700	0.60100
H	9.70000	3.14300	-0.25000	H	9.25600	-3.53700	0.05500	H	9.58300	3.34900	-0.02700
H	7.95200	4.26500	3.52300	H	7.00200	-4.70900	-3.42000	H	7.52000	4.35700	3.61700
H	6.08200	4.11400	2.14200	H	5.32200	-4.29300	-1.87700	H	5.76500	4.14700	2.09800
H	8.69000	1.14500	0.41100	H	8.36000	-1.49000	-0.64000	H	8.61300	1.30200	0.54600
H	4.09800	2.69900	1.71000	H	3.52900	-2.65100	-1.36800	H	3.87800	2.65200	1.51800
H	8.39000	-1.30500	-0.76200	H	8.39600	1.07400	0.37200	H	8.50000	-1.15000	-0.65300
H	5.10500	-3.95500	-1.65300	H	5.44800	4.07200	1.33600	H	5.40500	-3.94600	-1.75400
H	3.48900	-2.37500	-0.64500	H	3.60900	2.56800	0.61400	H	3.65700	-2.44600	-0.85100
H	10.62300	-3.85100	-3.80500	H	11.12300	3.62000	2.97500	H	11.03700	-3.57100	-3.55500
H	10.28500	-6.29700	-3.44800	H	10.95500	6.05700	2.46700	H	10.79100	-6.03200	-3.22900
H	8.34800	-7.08500	-2.08800	H	8.96400	6.91900	1.23500	H	8.81200	-6.91900	-1.99800
H	6.78300	-5.44900	-1.08200	H	7.17900	5.36300	0.50500	H	7.11200	-5.36300	-1.08700
H	9.03100	-2.21800	-2.83200	H	9.31400	2.07400	2.28200	H	9.31300	-2.02000	-2.67900
H	0.69100	0.75800	3.16900	H	0.25800	-0.36400	-2.79700	H	0.65400	0.55300	3.06500
H	3.17200	0.61800	2.76800	H	2.76500	-0.43600	-2.58400	H	3.13900	0.56300	2.62200

Table S27: Coordinates of the optimized minimum in the S_0, S_1, and T_1 of 36PCD at the MN15/cc-pVDZ level.

S_0				S_1				T_1			
C	-3.27000	6.88800	-0.62000	C	-4.58700	6.99300	-0.45500	C	-3.74900	7.00000	-0.29700
C	-4.53800	7.47000	-0.56500	C	-5.90500	7.34900	-0.15600	C	-5.04700	7.51500	-0.32700
C	-5.67000	6.65200	-0.56400	C	-6.87200	6.35300	0.01000	C	-6.13100	6.64000	-0.42200
C	-5.53400	5.26500	-0.61700	C	-6.52200	5.01100	-0.11800	C	-5.91900	5.26300	-0.48800
C	-4.26500	4.66600	-0.67000	C	-5.20000	4.63800	-0.42500	C	-4.61900	4.73100	-0.45700
C	-3.13500	5.50000	-0.67100	C	-4.23800	5.65200	-0.59300	C	-3.53800	5.62200	-0.36100
C	-3.12000	2.59300	-1.52100	C	-3.82400	2.82100	-1.47900	C	-3.30100	2.74300	-1.25300
C	-4.12300	3.18800	-0.72100	C	-4.82900	3.21600	-0.56700	C	-4.39300	3.26500	-0.52400
C	-4.97900	2.36100	0.01400	C	-5.48000	2.22800	0.21800	C	-5.25600	2.37600	0.12800
C	-4.82500	0.97300	-0.04400	C	-5.11200	0.90300	0.08700	C	-5.01800	1.00100	0.05900
C	-3.80200	0.40800	-0.83700	C	-4.09300	0.54100	-0.83400	C	-3.90600	0.50900	-0.66100
C	-2.94800	1.21500	-1.59500	C	-3.44200	1.49300	-1.63100	C	-3.04300	1.37800	-1.33600
N	-3.86800	-0.98700	-0.73900	N	-3.90000	-0.82300	-0.79100	N	-3.90400	-0.89100	-0.59700
C	-5.53600	-0.13000	0.57400	C	-5.54500	-0.34500	0.71700	C	-5.71600	-0.15400	0.59600
C	-6.60900	-0.19500	1.46700	C	-6.48200	-0.66800	1.67900	C	-6.85500	-0.29900	1.39100
C	-7.06900	-1.43800	1.91400	C	-6.65300	-2.02700	2.06000	C	-7.28000	-1.57800	1.77100
C	-6.42600	-2.60900	1.45400	C	-5.84800	-3.02400	1.45700	C	-6.52700	-2.69900	1.35600
C	-5.35600	-2.57200	0.56500	C	-4.89200	-2.72100	0.49700	C	-5.38600	-2.58000	0.56900
C	-4.92200	-1.31900	0.11900	C	-4.75800	-1.37200	0.12800	C	-5.00000	-1.29500	0.17200
C	-9.41400	-0.66300	4.79800	C	-8.93500	-1.85100	5.07700	C	-9.95200	-1.02900	4.41500
C	-10.35700	-1.68700	4.68300	C	-9.59100	-3.08300	5.00200	C	-10.80800	-2.10800	4.18000
C	-10.22400	-2.63200	3.66300	C	-9.28600	-3.96900	3.96400	C	-10.50900	-3.01300	3.15900

C	-9.15900	-2.55200	2.76500	C	-8.32900	-3.62900	3.01200	C	-9.36500	-2.83900	2.38000
C	-8.20500	-1.52700	2.86900	C	-7.66300	-2.38900	3.07200	C	-8.49600	-1.76000	2.60500
C	-8.34900	-0.58600	3.90100	C	-7.98500	-1.50500	4.12000	C	-8.80800	-0.85800	3.63600
H	-2.37700	7.51700	-0.61300	H	-3.82500	7.76400	-0.57400	H	-2.89400	7.67400	-0.21500
H	-4.64300	8.55600	-0.52500	H	-6.17800	8.40000	-0.05100	H	-5.21200	8.59300	-0.27700
H	-6.66700	7.09700	-0.53100	H	-7.90500	6.62400	0.23200	H	-7.15000	7.03200	-0.45500
H	-6.42500	4.63300	-0.64500	H	-7.29100	4.24200	-0.01600	H	-6.77100	4.58600	-0.59100
H	-2.13900	5.05200	-0.68500	H	-3.20000	5.38300	-0.79800	H	-2.52100	5.22600	-0.30900
H	-2.47900	3.23900	-2.12400	H	-3.35400	3.58000	-2.10500	H	-2.65200	3.43600	-1.79300
H	-5.75400	2.80000	0.64800	H	-6.24400	2.52500	0.94000	H	-6.10200	2.75700	0.70600
H	-2.18300	0.78400	-2.24400	H	-2.67600	1.19000	-2.34500	H	-2.20500	1.00500	-1.92600
H	-7.09900	0.72400	1.80100	H	-7.11200	0.10400	2.12400	H	-7.42500	0.58200	1.69900
H	-6.76200	-3.57600	1.83400	H	-5.95900	-4.05800	1.78600	H	-6.83300	-3.69100	1.69500
H	-4.86300	-3.49200	0.25000	H	-4.25900	-3.48400	0.04600	H	-4.80300	-3.46100	0.29700
H	-9.50300	0.07500	5.59700	H	-9.15900	-1.16000	5.89100	H	-10.17100	-0.32000	5.21600
H	-11.19000	-1.75000	5.38500	H	-10.33800	-3.35200	5.75000	H	-11.70300	-2.24300	4.79100
H	-10.95900	-3.43300	3.55900	H	-9.80200	-4.92700	3.89300	H	-11.17500	-3.85500	2.96200
H	-9.07800	-3.27900	1.95300	H	-8.11800	-4.31600	2.19000	H	-9.15300	-3.53600	1.56500
H	-7.59900	0.20100	4.01600	H	-7.45700	-0.55200	4.20500	H	-8.12800	-0.02800	3.84400
C	-1.60600	-1.72500	-1.24600	C	-1.61700	-1.37400	-1.41300	C	-1.62700	-1.54100	-1.11700
C	-0.75100	-2.63000	-1.87200	C	-0.70300	-2.09800	-2.20700	C	-0.72200	-2.41100	-1.79900
C	-1.29600	-3.70100	-2.59000	C	-1.26100	-3.01400	-3.17100	C	-1.29000	-3.50100	-2.58600
C	-2.66500	-3.89700	-2.71300	C	-2.61900	-3.20000	-3.34600	C	-2.65200	-3.71700	-2.68500
C	-3.51800	-2.97800	-2.10000	C	-3.52400	-2.48500	-2.55000	C	-3.51600	-2.84700	-2.01500
C	-2.99300	-1.90100	-1.36600	C	-2.97300	-1.57400	-1.59900	C	-2.98400	-1.75400	-1.22400
S	-0.01900	-4.73500	-3.28700	S	0.01500	-3.84300	-4.07300	S	-0.02900	-4.45500	-3.38300
C	2.63200	-3.92300	-2.70100	C	2.64400	-3.15200	-3.35300	C	2.59900	-3.75700	-2.65900
C	1.26500	-3.71300	-2.58400	C	1.27700	-2.98800	-3.17300	C	1.23800	-3.51900	-2.57500
C	0.72800	-2.63700	-1.87000	C	0.72600	-2.08600	-2.21700	C	0.68000	-2.42000	-1.79500
C	1.58900	-1.73800	-1.24400	C	1.63300	-1.34500	-1.42400	C	1.59200	-1.55900	-1.11000
C	2.97400	-1.92700	-1.36000	C	2.99800	-1.50200	-1.61400	C	2.94600	-1.79200	-1.20500
C	3.49200	-3.01000	-2.08800	C	3.52500	-2.40000	-2.57500	C	3.46900	-2.89800	-1.98300
O	-0.02700	-6.06100	-2.65500	O	0.02500	-5.29300	-3.78400	O	-0.04100	-5.85200	-2.92200
O	-0.01400	-4.63400	-4.75200	O	0.00600	-3.45400	-5.49900	O	-0.02100	-4.19700	-4.83200
H	-1.22000	-0.89500	-0.65100	H	-1.27000	-0.67200	-0.65000	H	-1.25300	-0.72700	-0.49300
H	-4.60100	-3.07400	-2.19500	H	-4.60200	-2.57100	-2.67500	H	-4.59700	-2.94300	-2.11000
H	1.20900	-0.90200	-0.65300	H	1.27900	-0.65300	-0.65600	H	1.22500	-0.73500	-0.49500
C	10.17300	-2.68900	3.70800	C	10.05300	-3.43500	3.31200	C	10.42000	-3.11100	3.24300
C	10.31300	-1.73700	4.72100	C	10.26600	-2.64900	4.44700	C	10.73300	-2.19300	4.24800
C	9.38100	-0.70200	4.82400	C	9.41100	-1.57500	4.70500	C	9.89700	-1.09400	4.46000
C	8.32000	-0.62100	3.92300	C	8.35400	-1.29300	3.83900	C	8.75800	-0.91600	3.67400
C	8.16900	-1.57000	2.89800	C	8.13000	-2.07300	2.69300	C	8.43300	-1.83100	2.65900
C	9.11300	-2.60500	2.80600	C	8.99900	-3.14900	2.44500	C	9.28300	-2.93100	2.45700
C	6.38300	-2.64500	1.48700	C	6.28700	-2.81400	1.14700	C	6.45000	-2.75600	1.42000
C	7.03800	-1.47600	1.93900	C	7.01000	-1.76700	1.76700	C	7.22400	-1.64100	1.81700
C	6.59400	-0.23200	1.47700	C	6.65100	-0.44300	1.49300	C	6.82600	-0.36100	1.41100
C	5.52500	-0.16500	0.58000	C	5.59800	-0.17600	0.61200	C	5.69300	-0.20900	0.60800
C	4.90000	-1.35000	0.13400	C	4.90200	-1.24400	-0.00200	C	4.95600	-1.34400	0.20300
C	5.31700	-2.60400	0.59400	C	5.23600	-2.57700	0.26900	C	5.31500	-2.62900	0.62600

N	3.85400	-1.01600	-0.73400	N	3.90200	-0.73300	-0.82200	N	3.87300	-0.93400	-0.58000
C	4.82700	0.94100	-0.05000	C	4.98300	1.04600	0.13100	C	5.02000	0.94800	0.04500
C	4.99900	2.32800	-0.01100	C	5.23200	2.40300	0.35700	C	5.29000	2.31800	0.07900
C	4.16400	3.15500	-0.77000	C	4.46200	3.37100	-0.29700	C	4.45900	3.20900	-0.61100
C	3.16500	2.56100	-1.57500	C	3.44200	2.95000	-1.18300	C	3.36200	2.69300	-1.33800
C	2.97200	1.18400	-1.63000	C	3.17000	1.60800	-1.42100	C	3.07000	1.33300	-1.38300
C	3.80600	0.37700	-0.84900	C	3.94700	0.65500	-0.75100	C	3.90500	0.46300	-0.67500
C	4.80800	6.68000	0.48500	C	5.30600	6.66100	1.40700	C	5.45000	6.66900	0.59700
C	4.64600	7.43200	-0.68100	C	5.20100	7.56700	0.34900	C	5.24900	7.44000	-0.55100
C	4.32500	6.78500	-1.87600	C	4.85200	7.09900	-0.92000	C	4.78800	6.82700	-1.71800
C	4.16900	5.39900	-1.90600	C	4.61200	5.74100	-1.12900	C	4.53100	5.45600	-1.73700
C	4.32700	4.63100	-0.74100	C	4.71300	4.81800	-0.07400	C	4.72800	4.67000	-0.59000
C	4.64800	5.29400	0.45500	C	5.06200	5.30300	1.19800	C	5.19100	5.29900	0.57700
H	10.90000	-3.49800	3.61400	H	10.72000	-4.27300	3.09400	H	11.07100	-3.96900	3.06300
H	11.14300	-1.80300	5.42700	H	11.09200	-2.87200	5.12500	H	11.62400	-2.33300	4.86400
H	9.47500	0.04200	5.61700	H	9.56100	-0.95800	5.59400	H	10.12700	-0.37500	5.24900
H	7.57800	0.17500	4.02800	H	7.67200	-0.46900	4.06500	H	8.09400	-0.07000	3.86500
H	9.02600	-3.33800	2.00000	H	8.85800	-3.75200	1.54500	H	9.06000	-3.63800	1.65400
H	6.70600	-3.61200	1.87800	H	6.54900	-3.84600	1.39000	H	6.73600	-3.74700	1.77900
H	7.09400	0.68300	1.80300	H	7.20800	0.38100	1.94600	H	7.41300	0.51300	1.70300
H	4.81400	-3.52200	0.28500	H	4.68200	-3.39800	-0.18800	H	4.71500	-3.50300	0.36700
H	5.80300	2.76300	0.58700	H	6.04900	2.70800	1.01700	H	6.16700	2.69100	0.61500
H	2.50700	3.21000	-2.15800	H	2.83400	3.70900	-1.67900	H	2.70700	3.39100	-1.86500
H	2.19700	0.75600	-2.26600	H	2.37900	1.30200	-2.10700	H	2.21900	0.96600	-1.95700
H	5.05000	7.17600	1.42700	H	5.57200	7.01400	2.40600	H	5.80300	7.14000	1.51700
H	4.76900	8.51600	-0.65700	H	5.39100	8.62900	0.51200	H	5.45100	8.51200	-0.53600
H	4.20500	7.36200	-2.79600	H	4.77700	7.79600	-1.75800	H	4.63600	7.41700	-2.62300
H	3.94600	4.89800	-2.85100	H	4.37100	5.38000	-2.13100	H	4.19800	4.97900	-2.66200
H	4.74800	4.71600	1.37700	H	5.12000	4.60500	2.03600	H	5.32300	4.70800	1.48700
H	3.02100	-4.76900	-3.27100	H	3.01700	-3.85200	-4.10300	H	2.98100	-4.58700	-3.25600
H	4.57400	-3.11800	-2.17700	H	4.60500	-2.47700	-2.70200	H	4.54900	-3.01300	-2.06600
H	-3.06000	-4.73800	-3.28600	H	-2.97900	-3.90100	-4.10300	H	-3.04100	-4.53800	-3.28900
